# Variable selection-combined causal mediation analysis for continuous treatments with application to large-dimensional biomedical data

**DOI:** 10.1371/journal.pcbi.1014436

**Published:** 2026-06-24

**Authors:** Yajing Zhou, Kecheng Wei, Yahang Liu, Zhaoyang Li, Chen Huang, Guoyou Qin, Yongfu Yu

**Affiliations:** 1 Department of Biostatistics, NHC Key Laboratory for Health Technology Assessment, Key Laboratory of Public Health Safety of Ministry of Education, School of Public Health, Fudan University, Shanghai China; 2 Shanghai Institute of Infectious Disease and Biosecurity, Fudan University, Shanghai, China; 3 Shanghai Key Laboratory of Gene Editing and Cell Therapy for Rare Diseases, Fudan University, Shanghai, China; UniversitatsSpital Zurich, SWITZERLAND

## Abstract

Substantial progress has been made in the area of causal inference utilizing large-scale data, among which the estimation of causal mediation effects has attracted a lot of attention. However, existing large-dimensional causal inference primarily focuses on total effects or typical causal mediation effects under binary variable settings, placing less emphasis on large-scale covariate selection with continuous treatment and mediator. To address this, we propose a weighted semiparametric estimation framework that integrates the generalized outcome-adaptive LASSO method into generalized propensity score modeling to achieve estimation of causal mediation effects under continuous variable settings. Simulation results show that our proposed method outperforms other regularization-based methods in selection accuracy and estimation efficiency, which is achieved by incorporating outcome-related key variables and excluding noise covariates. From the perspective of achieving a stable balance between efficiency and bias, as well as high-dimensional information filtering, our method may serve as a compelling alternative that balances estimation efficiency with model interpretability and inferential robustness. We further conduct a real-world application based on the UK Biobank database, quantifying the causal mediation effects of apolipoprotein B levels within the association between potential diabetes risk and cancer incidence using large-scale healthcare and medical data.

## Author summary

Disease development and progress are well recognized to be influenced by multiple factors, and exploring the causal mediation effects of the mediator in the exposure-outcome association can help reveal the etiological mechanisms. Due to the widespread application of large-scale biology and health data, it is challenging to precisely select all important variables based on prior knowledge to obtain accurate estimates. In this study, we propose a generalized outcome-adaptive LASSO (GOAL)-combined weighted semiparametric approach to estimate the natural direct and indirect effects of continuous treatment and mediator in large-scale covariate settings. Our method extends previous work by allowing for accurate causal mediation estimates for continuous treatment and mediator with large-dimensional covariates, and also improves estimation efficiency by precisely incorporating outcome-related variables. We apply the proposed method to investigate the mediating role of apolipoprotein B in the association between potential diabetes risk and cancer incidence under extensive candidate covariates from biomedical and healthcare data.

## 1 Introduction

Beyond the total causal effect of a treatment on an outcome, which has been of primary interest in a large portion of existing investigations, the causal mechanisms through which the treatment functions on the outcome have also attracted considerable attention. In the biomedical field, the onset of disease often involves elaborate internal protein-dependent metabolic processes. Therefore, clinicians and healthcare practitioners may seek to disentangle unexplored pathways linked to particular candidate biomarkers from these interwoven connections, considering them as promising indirect targets for novel therapeutic strategies. Causal mediation analysis can decompose the total causal effect of a treatment on an outcome into the natural direct effect (NDE) and the natural indirect effect (NIE) mediated through an intermediate variable called the mediator [1,2]. Nevertheless, constrained by the lack of randomization, real-world practical biomedical research often builds causal inference on observational studies that are subjected to high levels of confounding, leading to biased causal estimates.

Among the current approaches for causal mediation analyses, propensity score (PS) methods are broadly used to reduce the influence of confounding bias and clarify causality [3–5]. The PS is defined as the probability of being treated conditional on the covariates, which can be estimated by fitting a logistic model in a common setting of binary treatment [6]. Given that continuous treatment density is also of great concern in many empirical problems [7,8], there has been a growing trend of studies targeting the causal mediation effects of a continuous treatment [5,9,10]. For a continuous treatment, using traditional PS methods by dichotomizing the continuous variable will violate the consistency assumption and cause causality distortion [11,12]. The generalized propensity score (GPS) was therefore proposed to solve this problem [13]. The GPS is measured as the conditional density of treatment given the covariates, which can be derived by fitting a model of treatment on covariates and specifying the distribution of the error term [13–15]. When employing the PS model to estimate the treatment effects, its high sensitivity to the covariates included needs to be taken into account. Inadequate adjustment for confounders can introduce bias and overcorrection for instrumental variables that only predict the treatment can elicit variance inflation [16,17], while the incorporation of outcome information by including prognostic variables may improve efficiency [18]. Therefore, determining the variables included in the PS model imposes an important influence on both the bias and efficiency of the estimation.

With the widespread availability of large-dimensional data, such as large-scale healthcare and medical information, omics data, etc., it becomes increasingly challenging to precisely prespecify and determine all the practically and statistically important variables by prior knowledge. Thereby, appropriate variable selection methods play a crucial role in identifying critical covariates and improving the estimation of causal effects in the context of extensive candidate covariates. A sequence of studies has explored various penalization-based variable selection strategies based on PS methods within the causal inference framework [19–23]. Tibshirani proposed the least absolute shrinkage and selection operator (LASSO) method that enables variable selection in the PS model with a large number of predictors by shrinking the coefficients of those less important predictors to zero [19]. Zou then extended the conventional LASSO to the Adaptive LASSO (AdaLASSO) method by applying the adaptive weights constructed by the maximum likelihood estimates to the penalty, facilitating a more accurate selection of predictor variables and allowing for multicollinearity [20]. Shortreed further developed the outcome-adaptive LASSO (OAL) method that can improve estimation efficiency by incorporating outcome information into the adaptive weights, ultimately selecting a covariate set with all the confounders and prognostic variables [21]. Considering the limited application of the OAL method to binary treatments, Gao et al. introduced the generalized outcome-adaptive LASSO (GOAL) method, which emphasized variable selection in the GPS models for continuous treatment [22].

Beyond these penalization-based PS approaches, recent advances in modern machine learning have also introduced alternative strategies for causal mediation analysis in high-dimensional settings [24–30]. These methods leverage flexible machine learning techniques to accommodate high-dimensional covariates and nonlinear relationships, reducing reliance on parametric assumptions and enhancing estimation under complex confounding structures. For instance, Yang et al.[26] proposed a partially linear mediation-based double machine learning method that employs orthogonal estimating equations to mitigate bias arising from regularized nuisance estimation in high-dimensional settings. While such approaches improve robustness against model misspecification and regularization bias, they may lack the capability to actively identify important variables from high-dimensional covariate space, potentially leading to reduced efficiency and limited interpretability of the covariate structure.

Therefore, despite these important developments, variable selection-based approaches for large-dimensional causal mediation analysis remain of substantial methodological and practical importance, as they offer the dual advantage of covariate selection and efficient effect estimation. Nevertheless, the integration of structured covariate selection into causal mediation analysis is still underexplored. Existing efforts integrating covariate selection procedures into causal inference have predominantly focused on the average total effects of treatments on outcomes, and there is only one study by Ye et al.[23] extending the approach to the causal mediation framework. Given that the work of Ye et al. has targeted primarily dichotomous treatment and mediator, we therefore proposed a method employing the appropriate variable selection procedure to causal mediation analyses tailored to continuous treatment and mediator to bridge the gap.

In this paper, we extend the work of Ye et al.[23] and develop a variable selection-embedded approach to estimate the causal mediation effects for continuous treatment and mediator, which integrates the GOAL method proposed by Gao et al.[22] to the semiparametric estimation method based on GPS weighting proposed by Huber et al [5]. Our method enables the identification of causal mediation effects in a large-scale covariate setting for continuous treatment and mediator. Also, we gain estimation efficiency by incorporating outcome information into the GPS model using GOAL-based variable selection. Simulation results suggest that our method outperforms other regularization method-integrated estimation in most settings, generally exhibiting higher estimation efficiency and a more accurate variable selection performance by including outcome-related covariates and excluding instrumental variables and spurious variables.

The remainder of this article is structured as follows. In Section 2, we demonstrate the estimation results of our simulation study and real data application. Section 3 provides discussions and conclusions of this study. In Section 4, we first review the GPS-weighted semiparametric estimation approach of causal mediation effects and illustrate the extension of the GOAL method to the causal mediation framework. Then, we introduce the simulation study of our proposed method with other competing methods in different scenarios, and the empirical analysis based on large-scale individual healthcare and biomedical data from the UK Biobank database.

## 2 Results

### 2.1 Simulation results

**Fig 1:**
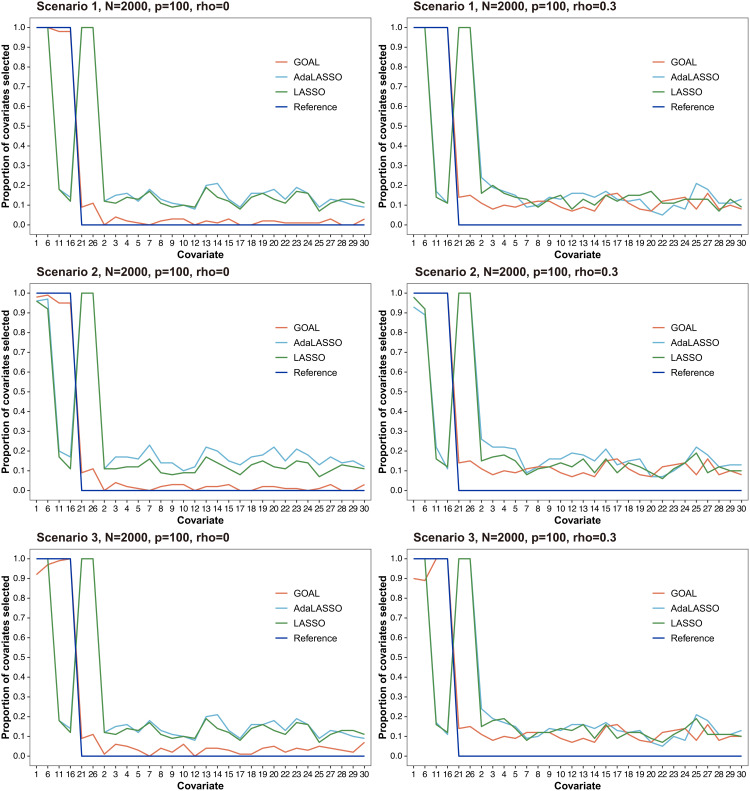
Proportion of the top 30 covariates selected using GOAL, AdaLASSO, and LASSO-based methods in the treatment generalized propensity score model given covariates under Scenarios 1–3 with (n, p) = (2000, 100). **Notes:** The horizontal axis represents the index of the simulated covariates, and the vertical axis denotes the proportion of covariates repeatedly selected across 100 simulations. Abbreviations: LASSO, the least absolute shrinkage and selection operator; GOAL, generalized outcome-adaptive LASSO; AdaLASSO, adaptive LASSO.

**Fig 2:**
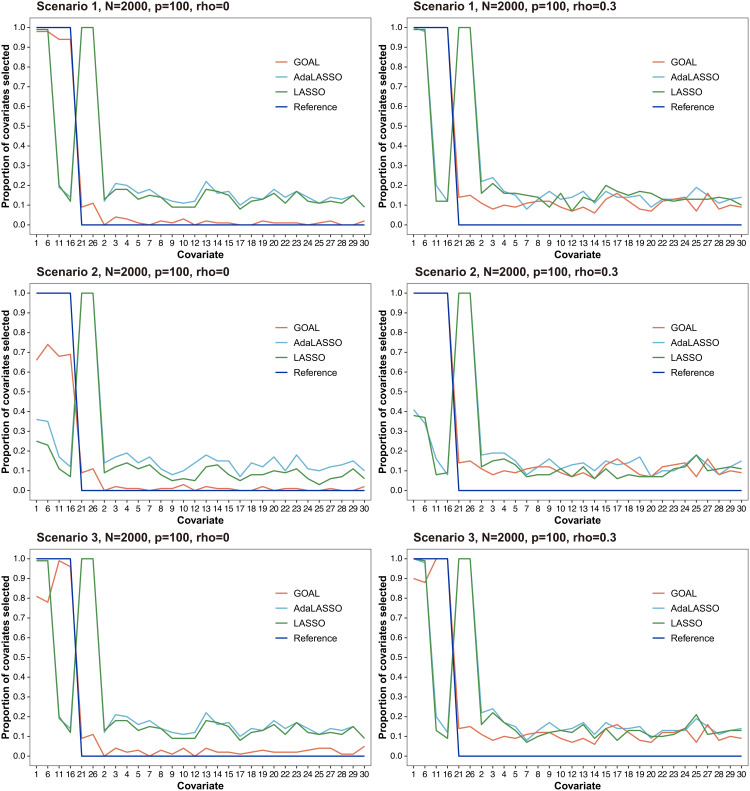
Proportion of the top 30 covariates selected using GOAL, AdaLASSO, and LASSO-based methods in the treatment generalized propensity score model given both covariates and the mediator under Scenarios 1–3 with (n, p) = (2000, 100). **Notes:** The horizontal axis represents the index of the simulated covariates, and the vertical axis denotes the proportion of covariates repeatedly selected across 100 simulations. Abbreviations: LASSO, the least absolute shrinkage and selection operator; GOAL, generalized outcome-adaptive LASSO; AdaLASSO, adaptive LASSO.

**Fig 3:**
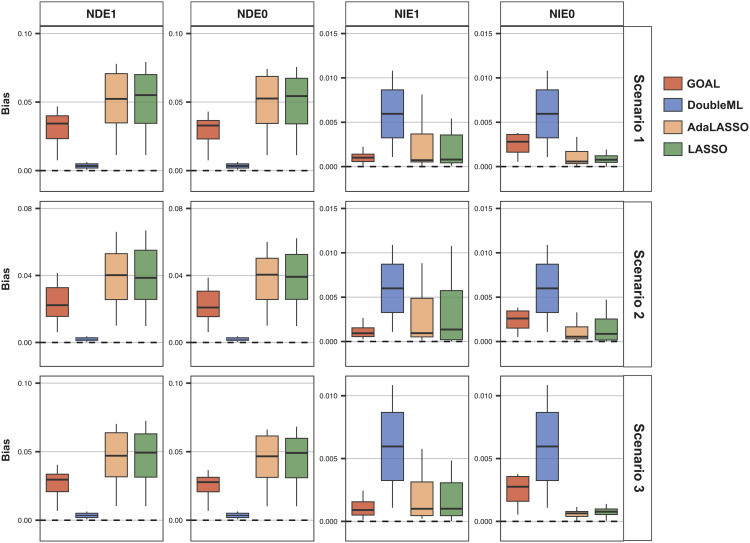
Comparison of absolute bias in causal mediation effect estimation across different scenarios with a sample size of 2000. **Note:** The figure illustrates the distribution of absolute bias (calculated by the absolute value of the difference between the estimates and the true values across the exposure range) for the proposed GOAL-based method and competing approaches. The dotted line indicates the state with zero absolute bias. The results are based on the setting of sample size *n* = 2000 and covariate correlation (*ρ*) of 0. The NDE1 and NDE0 separately represent natural direct effects (NDE) under treatment and non-treatment, and the NIE1 and NIE0 separately represent natural indirect effects (NIE) under treatment and non-treatment. Abbreviation: LASSO, the least absolute shrinkage and selection operator; GOAL, generalized outcome-adaptive LASSO; AdaLASSO, adaptive LASSO; DoubleML, double machine learning.

**Fig 4:**
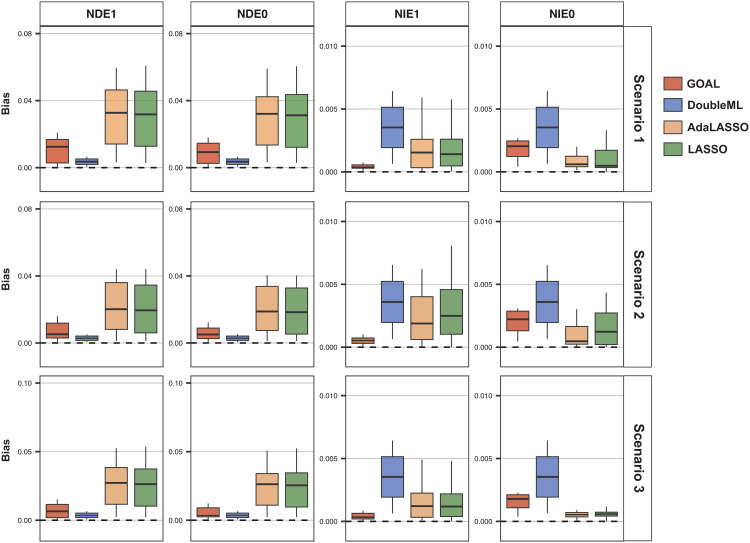
Comparison of absolute bias in causal mediation effect estimation across different scenarios with a sample size of 5000. **Note:** The figure illustrates the distribution of absolute bias (calculated by the absolute value of the difference between the estimates and the true values across the exposure range) for the proposed GOAL-based method and competing approaches. The dotted line indicates the state with zero absolute bias. The results are based on the setting of sample size *n* = 5000 and covariate correlation (*ρ*) of 0. The NDE1 and NDE0 separately represent natural direct effects (NDE) under treatment and non-treatment, and the NIE1 and NIE0 separately represent natural indirect effects (NIE) under treatment and non-treatment. Abbreviation: LASSO, the least absolute shrinkage and selection operator; GOAL, generalized outcome-adaptive LASSO; AdaLASSO, adaptive LASSO; DoubleML, double machine learning.

The detailed description of the simulation study is shown in the Method section. The variable selection performances of different regularization methods, showing the proportion of the first 30 covariates selected for two GPS models in 100 replications, are visualized in Figs 1–2 and Figs A-B in S2 Appendix. The direct and indirect mediation effects estimated by the proposed method and other comparing methods based on 100 replications are presented in Tables 1–3 and Table 3 Tables A-C in S1 Appendix, comprehensively showing the estimation performance under different settings, with respect to the association strengths of covariate with outcome or treatment, the kernel procedures by standard or undersmoothing bandwidths, covariate correlation of *ρ* = 0 or 0.3, and sample sizes and covariate dimensions (*n*, *p*) = (2000, 100) or (5000, 200). The estimation performance under typical scenarios is further visualized to comprehensively illustrate the estimation accuracy and stability across the exposure spectrum by different methods (Figs 3–4).

**Table 1:** Mediation effects estimated by weighting with a parametric generalized propensity score based on different covariate sets under Scenario 1 (SoSt) using an undersmoothing kernel bandwidth.

Sample Size	Methods	Correlation (rho)	${\hat{\theta }}_{a,a\text{'}}\left(a\right)$				${\hat{\theta }}_{a,a\text{'}}\left(a\text{'}\right)$				${\hat{\delta }}_{a,a\text{'}}\left(a\right)$				${\hat{\delta }}_{a,a\text{'}}\left(a\text{'}\right)$			
**Bias (%)**	**SD**	**RMSE**	**CP (%)**	**Bias (%)**	**SD**	**RMSE**	**CP (%)**	**Bias (%)**	**SD**	**RMSE**	**CP (%)**	**Bias (%)**	**SD**	**RMSE**	**CP (%)**
***n* = 2000**	GOAL	0	6.33	0.085	0.098	90.3	6.09	0.085	0.098	90.6	3.15	0.011	0.011	96.1	5.46	0.011	0.009	98.0
		0.3	6.83	0.097	0.107	93.2	6.89	0.097	0.106	93.1	3.59	0.012	0.012	95.3	4.37	0.011	0.012	92.8
	AdaLASSO	0	9.88	0.094	0.111	90.0	9.73	0.093	0.110	89.9	3.79	0.013	0.013	96.2	2.24	0.012	0.011	98.3
		0.3	13.26	0.104	0.127	90.0	13.84	0.104	0.129	88.8	13.29	0.014	0.015	93.7	19.68	0.013	0.016	86.9
	LASSO	0	10.01	0.094	0.112	63.8	9.84	0.093	0.111	64.1	3.72	0.013	0.012	96.1	2.35	0.012	0.011	97.1
		0.3	14.39	0.102	0.130	42.5	14.76	0.102	0.132	40.8	13.26	0.013	0.013	91.4	17.36	0.012	0.015	37.6
	DoubleML	0	0.62	0.013	0.012	97.0	0.62	0.013	0.012	97.0	12.01	0.006	0.008	83.0	12.01	0.006	0.008	83.0
		0.3	1.15	0.013	0.014	93.0	1.15	0.013	0.014	93.0	11.78	0.006	0.008	87.0	11.78	0.006	0.008	87.0
	Benchmark (True)	0	9.10	0.092	0.111	92.2	8.97	0.092	0.110	92.4	3.37	0.012	0.012	88.7	2.16	0.011	0.010	86.4
		0.3	12.03	0.102	0.130	83.1	12.38	0.102	0.132	87.8	8.01	0.013	0.013	29.8	11.86	0.012	0.015	19.3
	Benchmark (Outcome)	0	5.26	0.085	0.096	90.6	5.03	0.085	0.096	91.2	3.22	0.011	0.010	95.7	5.54	0.011	0.009	98.0
		0.3	5.09	0.095	0.107	91.6	5.01	0.095	0.107	92.3	2.76	0.012	0.012	94.2	2.91	0.011	0.011	98.8
	Benchmark (True+Outcome)	0	9.10	0.092	0.110	92.1	8.97	0.092	0.110	92.6	3.25	0.012	0.011	89.2	2.10	0.011	0.010	87.8
		0.3	11.87	0.102	0.129	86.3	12.22	0.102	0.131	90.7	7.92	0.013	0.013	48.8	11.86	0.012	0.015	34.9
	Benchmark (Full)	0	9.00	0.093	0.111	89.7	8.85	0.093	0.111	90.2	3.28	0.012	0.012	96.9	2.12	0.011	0.010	99.4
		0.3	11.65	0.103	0.129	87.8	11.98	0.102	0.130	86.5	8.31	0.013	0.014	92.4	11.99	0.012	0.015	93.5
***n* = 5000**	GOAL	0	1.90	0.063	0.063	94.2	1.68	0.063	0.063	94.2	1.38	0.008	0.008	94.0	3.98	0.007	0.008	94.0
		0.3	5.88	0.073	0.079	91.9	5.93	0.073	0.080	91.3	3.50	0.009	0.009	93.3	4.13	0.008	0.009	89.9
	AdaLASSO	0	4.99	0.070	0.075	93.0	5.46	0.070	0.074	93.3	5.25	0.009	0.009	95.6	3.10	0.009	0.008	98.0
		0.3	4.89	0.077	0.104	88.2	5.30	0.077	0.107	86.8	5.14	0.010	0.011	89.2	2.99	0.009	0.014	83.3
	LASSO	0	11.10	0.070	0.075	93.0	10.57	0.069	0.074	93.3	11.08	0.009	0.009	94.9	11.39	0.008	0.008	96.6
		0.3	11.62	0.077	0.104	86.0	11.22	0.076	0.107	84.8	11.79	0.009	0.011	90.2	9.76	0.009	0.013	83.6
	DoubleML	0	1.40	0.008	0.009	94.0	1.40	0.008	0.009	94.0	8.21	0.003	0.005	85.0	8.21	0.003	0.005	85.0
		0.3	1.34	0.008	0.009	91.0	1.34	0.008	0.009	91.0	9.11	0.003	0.005	82.0	9.11	0.003	0.005	82.0
	Benchmark (True)	0	4.58	0.069	0.075	94.2	4.42	0.069	0.075	94.7	2.17	0.008	0.008	85.7	1.85	0.008	0.008	82.7
		0.3	9.57	0.076	0.104	75.4	10.05	0.076	0.108	82.8	6.35	0.009	0.010	11.8	11.62	0.009	0.013	3.2
	Benchmark (Outcome)	0	1.37	0.063	0.063	94.2	1.17	0.063	0.062	94.2	1.67	0.008	0.008	94.0	4.60	0.007	0.007	94.0
		0.3	3.45	0.071	0.078	91.3	3.49	0.071	0.079	91.1	1.58	0.009	0.009	94.0	2.04	0.008	0.009	92.2
	Benchmark (True+Outcome)	0	4.65	0.069	0.075	94.2	4.50	0.069	0.075	94.7	2.23	0.008	0.008	85.3	1.82	0.008	0.008	81.4
		0.3	9.61	0.076	0.104	83.0	10.08	0.076	0.108	88.5	6.46	0.009	0.010	28.7	11.66	0.009	0.013	12.0
	Benchmark (Full)	0	4.63	0.069	0.075	92.7	4.47	0.069	0.074	92.5	2.29	0.008	0.008	95.2	1.76	0.008	0.008	97.1
		0.3	9.57	0.076	0.104	86.7	10.04	0.076	0.108	85.1	6.64	0.009	0.010	92.1	11.89	0.009	0.013	83.0

**Note:** SoSt indicates the scenario with both strong outcome and treatment (Scenario 1). ${\hat{\theta }}_{a,a\text{'}}(a)$, ${\hat{\theta }}_{a,a\text{'}}(a\text{'})$ separately represent direct effects under treatment and non-treatment, and ${\hat{\delta }}_{a,a\text{'}}(a)$, ${\hat{\delta }}_{a,a\text{'}}(a\text{'})$ separately represent indirect effects under treatment and non-treatment. “Bias (%)”, “SD”, “RMSE”, and “CP” respectively report the average relative absolute bias, standard deviation, root mean squared error, and coverage probability of the effects across all treatment values *a* ∈ {-1, -0.9,  ..., -0.1} ∪ {0.1,  ..., 0.9, 1} and *a’* = 0. Results of the GOAL, Adaptive LASSO, and LASSO methods are all based on a gamma convergence of 2. The undersmoothing kernel bandwidth is set to half of the semiparametric bandwidth, that is, (*C*·*n*^-0.25^)/2 with *C* = 2.34. To further compare the results of the non-regularization competing method, i.e., the double machine learning method proposed by Yang et al. (2025), we include its estimates in this table for better readability; however, this method does not involve a kernel procedure or kernel bandwidth.

Abbreviations: LASSO, the least absolute shrinkage and selection operator; GOAL, generalized outcome-adaptive LASSO; AdaLASSO, adaptive LASSO; DoubleML, double machine learning.

**Table 2:** Mediation effects estimated by weighting with a parametric generalized propensity score based on different covariate sets under Scenario 2 (SoWt) using an undersmoothing kernel bandwidth.

Sample Size	Methods	Correlation (rho)	${\hat{\theta }}_{a,a\text{'}}\left(a\right)$				${\hat{\theta }}_{a,a\text{'}}\left(a\text{'}\right)$				${\hat{\delta }}_{a,a\text{'}}\left(a\right)$				${\hat{\delta }}_{a,a\text{'}}\left(a\text{'}\right)$			
			Bias (%)	SD	RMSE	CP (%)	Bias (%)	SD	RMSE	CP (%)	Bias (%)	SD	RMSE	CP (%)	Bias (%)	SD	RMSE	CP (%)
***n* = 2000**	GOAL	0	4.82	0.084	0.093	91.8	4.60	0.084	0.094	92.1	3.15	0.011	0.011	96.3	5.12	0.011	0.009	96.9
		0.3	7.97	0.094	0.106	91.8	8.17	0.094	0.107	91.5	1.58	0.012	0.012	96.0	3.32	0.011	0.011	95.0
	AdaLASSO	0	7.84	0.093	0.107	90.3	7.64	0.093	0.107	90.2	4.27	0.014	0.013	96.2	1.86	0.013	0.012	96.8
		0.3	14.35	0.102	0.124	89.0	15.07	0.101	0.127	87.8	12.71	0.014	0.015	93.7	20.71	0.014	0.016	89.0
	LASSO	0	7.83	0.093	0.107	90.6	7.62	0.093	0.106	90.5	4.61	0.013	0.013	96.0	2.23	0.013	0.012	96.3
		0.3	15.70	0.101	0.134	87.5	16.34	0.101	0.137	85.5	7.61	0.014	0.013	96.0	14.77	0.013	0.015	91.0
	DoubleML	0	0.37	0.013	0.012	97.0	0.37	0.013	0.012	97.0	12.11	0.006	0.008	82.0	12.11	0.006	0.008	82.0
		0.3	1.13	0.013	0.014	92.0	1.13	0.013	0.014	92.0	11.88	0.006	0.008	87.0	11.88	0.006	0.008	87.0
	Benchmark (True)	0	2.86	0.091	0.105	91.1	3.53	0.091	0.105	91.9	30.43	0.012	0.011	92.1	37.86	0.011	0.010	89.4
		0.3	12.17	0.100	0.126	88.1	7.91	0.099	0.129	92.5	165.04	0.012	0.013	33.8	212.36	0.012	0.014	19.8
	Benchmark (Outcome)	0	4.49	0.084	0.091	92.4	4.27	0.084	0.092	92.4	3.40	0.011	0.010	96.6	5.65	0.011	0.009	95.7
		0.3	6.32	0.092	0.098	93.1	6.27	0.092	0.098	92.6	2.00	0.012	0.012	95.6	1.74	0.011	0.010	96.8
	Benchmark (True+Outcome)	0	2.87	0.091	0.105	91.0	3.53	0.091	0.105	91.8	30.76	0.012	0.011	92.4	38.20	0.011	0.010	89.5
		0.3	8.31	0.100	0.125	90.8	4.02	0.100	0.128	93.8	121.35	0.012	0.013	54.1	169.32	0.012	0.014	34.8
	Benchmark (Full)	0	7.87	0.092	0.105	90.0	7.79	0.092	0.105	90.1	3.42	0.012	0.011	97.3	2.01	0.011	0.010	99.1
		0.3	13.60	0.101	0.125	86.9	14.28	0.100	0.128	86.3	10.09	0.013	0.013	95.0	17.56	0.012	0.014	92.7
***n* = 5000**	GOAL	0	1.28	0.063	0.063	94.8	1.08	0.063	0.063	94.4	1.59	0.008	0.008	93.6	4.37	0.008	0.008	93.2
		0.3	7.51	0.070	0.078	91.8	7.78	0.070	0.079	90.7	2.19	0.009	0.009	93.9	3.49	0.008	0.008	92.1
	AdaLASSO	0	3.69	0.069	0.074	93.8	3.44	0.069	0.073	93.4	4.02	0.009	0.009	94.9	1.68	0.009	0.009	95.9
		0.3	12.48	0.076	0.102	87.2	13.24	0.075	0.108	85.4	11.13	0.010	0.011	90.4	19.60	0.010	0.015	79.3
	LASSO	0	3.51	0.069	0.074	93.6	3.28	0.069	0.073	94.0	4.99	0.009	0.009	94.5	2.49	0.009	0.008	95.1
		0.3	13.41	0.075	0.105	85.6	14.20	0.075	0.109	82.1	6.35	0.009	0.010	93.3	15.13	0.009	0.013	84.1
	DoubleML	0	1.34	0.008	0.009	94.0	1.34	0.008	0.009	94.0	8.32	0.003	0.005	85.0	8.32	0.003	0.005	85.0
		0.3	1.35	0.008	0.009	91.0	1.35	0.008	0.009	91.0	9.17	0.003	0.005	82.0	9.17	0.003	0.005	82.0
	Benchmark (True)	0	1.36	0.068	0.074	94.4	1.09	0.068	0.073	94.6	22.72	0.008	0.008	91.5	23.43	0.008	0.008	93.2
		0.3	13.92	0.074	0.102	81.5	8.97	0.074	0.106	90.4	158.10	0.009	0.010	16.0	213.17	0.008	0.012	3.0
	Benchmark (Outcome)	0	1.30	0.063	0.063	94.2	1.11	0.063	0.063	93.9	1.86	0.008	0.008	93.2	4.67	0.007	0.008	93.8
		0.3	6.97	0.068	0.074	91.6	6.84	0.069	0.074	91.5	2.12	0.008	0.009	94.8	2.72	0.008	0.007	89.9
	Benchmark (True+Outcome)	0	1.36	0.068	0.074	94.4	1.08	0.068	0.073	94.8	23.37	0.008	0.008	90.6	24.11	0.008	0.008	93.4
		0.3	9.72	0.074	0.103	87.4	4.93	0.074	0.107	93.3	115.04	0.009	0.010	35.4	168.24	0.008	0.012	13.4
	Benchmark (Full)	0	4.04	0.069	0.073	93.8	3.87	0.069	0.072	93.8	2.40	0.008	0.008	95.3	1.86	0.008	0.008	95.8
		0.3	14.02	0.075	0.103	84.0	14.67	0.074	0.107	82.8	6.26	0.009	0.010	94.7	13.41	0.008	0.012	85.2

**Note:** SoWt indicates the scenario with strong outcome and weak treatment (Scenario 2). ${\hat{\theta }}_{a,a\text{'}}(a)$, ${\hat{\theta }}_{a,a\text{'}}(a\text{'})$ separately represent direct effects under treatment and non-treatment, and ${\hat{\delta }}_{a,a\text{'}}(a)$, ${\hat{\delta }}_{a,a\text{'}}(a\text{'})$ separately represent indirect effects under treatment and non-treatment. “Bias (%)”, “SD”, “RMSE”, and “CP” respectively report the average relative absolute bias, standard deviation, root mean squared error, and coverage probability of the effects across all treatment values *a* ∈ {-1, -0.9,  ..., -0.1} ∪ {0.1,  ..., 0.9, 1} and *a’* = 0. Results of the GOAL, Adaptive LASSO, and LASSO methods are all based on a gamma convergence of 2. The undersmoothing kernel bandwidth is set to half of the semiparametric bandwidth, that is, (*C*·*n*^-0.25^)/2 with *C* = 2.34. To further compare the results of the non-regularization competing method, i.e., the double machine learning method proposed by Yang et al. (2025), we include its estimates in this table for better readability; however, this method does not involve a kernel procedure or kernel bandwidth.

Abbreviations: LASSO, the least absolute shrinkage and selection operator; GOAL, generalized outcome-adaptive LASSO; AdaLASSO, adaptive LASSO; DoubleML, double machine learning.

**Table 3:** Mediation effects estimated by weighting with a parametric generalized propensity score based on different covariate sets under Scenario 3 (WoSt) using an undersmoothing kernel bandwidth.

Sample Size	Methods	Correlation (rho)	${\hat{\theta }}_{a,a\text{'}}\left(a\right)$				${\hat{\theta }}_{a,a\text{'}}\left(a\text{'}\right)$				${\hat{\delta }}_{a,a\text{'}}\left(a\right)$				${\hat{\delta }}_{a,a\text{'}}\left(a\text{'}\right)$			
			Bias (%)	SD	RMSE	CP (%)	Bias (%)	SD	RMSE	CP (%)	Bias (%)	SD	RMSE	CP (%)	Bias (%)	SD	RMSE	CP (%)
***n* = 2000**	GOAL	0	5.52	0.083	0.094	90.9	5.29	0.083	0.094	91.2	3.10	0.011	0.010	95.8	5.39	0.011	0.009	96.1
		0.3	5.67	0.093	0.101	93.5	5.66	0.093	0.100	93.5	2.57	0.012	0.012	95.8	3.38	0.011	0.011	94.1
	AdaLASSO	0	8.93	0.092	0.107	90.3	8.74	0.092	0.106	90.9	3.70	0.013	0.012	96.7	2.01	0.012	0.011	97.8
		0.3	11.85	0.100	0.119	91.3	12.27	0.100	0.120	90.3	11.14	0.014	0.014	94.2	15.84	0.013	0.015	89.7
	LASSO	0	9.06	0.092	0.107	90.3	8.85	0.092	0.107	90.4	3.63	0.013	0.012	96.2	2.25	0.012	0.011	97.6
		0.3	13.04	0.100	0.124	90.1	13.27	0.099	0.125	89.5	11.03	0.013	0.013	94.8	13.58	0.013	0.014	91.0
	DoubleML	0	0.64	0.013	0.013	95.0	0.64	0.013	0.013	95.0	12.05	0.006	0.008	83.0	12.05	0.006	0.008	83.0
		0.3	1.16	0.013	0.014	92.0	1.16	0.013	0.014	92.0	11.75	0.006	0.008	87.0	11.75	0.006	0.008	87.0
	Benchmark (True)	0	2.82	0.091	0.106	92.0	3.67	0.090	0.106	92.2	33.38	0.012	0.011	91.2	43.29	0.011	0.010	91.4
		0.3	13.41	0.098	0.121	85.7	11.11	0.098	0.122	89.9	150.12	0.012	0.013	43.7	175.69	0.012	0.013	36.9
	Benchmark (Outcome)	0	5.26	0.083	0.093	90.9	5.03	0.083	0.093	91.5	3.22	0.011	0.010	95.8	5.54	0.010	0.009	98.1
		0.3	4.87	0.091	0.101	92.5	4.85	0.091	0.101	92.9	2.30	0.012	0.012	94.6	2.22	0.011	0.010	98.7
	Benchmark (True+Outcome)	0	2.81	0.090	0.106	92.2	3.67	0.090	0.106	92.1	33.68	0.012	0.011	91.5	43.65	0.011	0.010	91.2
		0.3	9.58	0.098	0.120	88.4	7.16	0.098	0.121	91.7	107.77	0.012	0.013	62.7	134.64	0.012	0.013	49.7
	Benchmark (Full)	0	9.09	0.091	0.107	90.0	8.93	0.091	0.106	90.7	3.20	0.012	0.011	96.9	2.24	0.011	0.010	99.2
		0.3	11.05	0.099	0.120	88.1	11.40	0.099	0.121	87.8	11.35	0.012	0.013	93.1	15.30	0.012	0.014	95.7
***n* = 5000**	GOAL	0	1.33	0.062	0.061	94.7	1.13	0.062	0.061	94.7	1.28	0.008	0.008	93.1	3.49	0.007	0.008	94.0
		0.3	4.95	0.070	0.074	92.2	4.95	0.070	0.074	91.8	2.88	0.009	0.009	93.6	3.39	0.008	0.008	91.4
	AdaLASSO	0	4.52	0.069	0.072	93.5	4.25	0.068	0.071	93.4	2.93	0.009	0.008	96.2	1.78	0.009	0.008	97.9
		0.3	9.30	0.074	0.095	89.2	9.66	0.074	0.098	88.1	9.04	0.009	0.010	91.5	13.08	0.009	0.012	88.5
	LASSO	0	4.36	0.068	0.072	93.3	4.13	0.068	0.071	93.4	2.75	0.009	0.008	95.3	1.68	0.008	0.008	96.2
		0.3	9.95	0.074	0.096	88.1	10.38	0.074	0.098	87.0	7.82	0.009	0.010	91.1	12.56	0.009	0.012	88.3
	DoubleML	0	1.40	0.008	0.009	94.0	1.40	0.008	0.009	94.0	8.22	0.003	0.005	85.0	8.22	0.003	0.005	85.0
		0.3	1.35	0.008	0.009	91.0	1.35	0.008	0.009	91.0	9.12	0.003	0.005	82.0	9.12	0.003	0.005	82.0
	Benchmark (True)	0	1.65	0.068	0.072	94.4	0.96	0.067	0.071	94.5	26.10	0.008	0.008	90.0	33.20	0.008	0.007	90.4
		0.3	14.64	0.073	0.094	81.4	11.71	0.073	0.096	87.0	141.13	0.009	0.010	21.3	173.62	0.008	0.011	11.9
	Benchmark (Outcome)	0	1.37	0.062	0.061	94.0	1.17	0.062	0.061	93.9	1.67	0.007	0.008	94.1	4.60	0.007	0.007	93.2
		0.3	5.00	0.068	0.071	92.7	4.93	0.068	0.071	92.7	1.91	0.008	0.009	94.0	2.85	0.008	0.008	93.0
	Benchmark (True+Outcome)	0	1.65	0.068	0.072	94.5	0.96	0.067	0.071	94.5	26.75	0.008	0.008	89.6	33.87	0.008	0.007	89.5
		0.3	10.52	0.073	0.094	87.2	7.67	0.073	0.096	91.0	99.55	0.009	0.010	44.8	131.16	0.008	0.011	27.1
	Benchmark (Full)	0	4.65	0.068	0.072	92.8	4.48	0.068	0.071	93.0	2.39	0.008	0.008	95.6	1.79	0.008	0.008	97.1
		0.3	10.96	0.073	0.093	88.6	11.40	0.073	0.096	87.4	7.05	0.009	0.010	92.9	12.03	0.008	0.011	87.6

**Note:** WoSt indicates the scenario with weak outcome and strong treatment (Scenario 3). ${\hat{\theta }}_{a,a\text{'}}(a)$, ${\hat{\theta }}_{a,a\text{'}}(a\text{'})$ separately represent direct effects under treatment and non-treatment, and ${\hat{\delta }}_{a,a\text{'}}(a)$, ${\hat{\delta }}_{a,a\text{'}}(a\text{'})$ separately represent indirect effects under treatment and non-treatment. “Bias (%)”, “SD”, “RMSE”, and “CP” respectively report the average relative absolute bias, standard deviation, root mean squared error, and coverage probability of the effects across all treatment values *a* ∈ {-1, -0.9,  ..., -0.1} ∪ {0.1,  ..., 0.9, 1} and *a’* = 0. Results of the GOAL, Adaptive LASSO, and LASSO methods are all based on a gamma convergence of 2. The undersmoothing kernel bandwidth is set to half of the semiparametric bandwidth, that is, (*C*·*n*^-0.25^)/2 with *C* = 2.34. To further compare the results of the non-regularization competing method, i.e., the double machine learning method proposed by Yang et al. (2025), we include its estimates in this table for better readability; however, this method does not involve a kernel procedure or kernel bandwidth.

Abbreviations: LASSO, the least absolute shrinkage and selection operator; GOAL, generalized outcome-adaptive LASSO; AdaLASSO, adaptive LASSO; DoubleML, double machine learning.

As shown in Figs 1–2 and Figs A-B in S2 Appendix, the GOAL method excels in selecting the prognostic variables (*X*_*11*_ and *X*_*16*_) with a proportion approaching 100% in most cases, while the AdaLASSO and LASSO methods, in contrast, have a low proportion. The GOAL method also outperforms other methods in selecting the true confounders (*X*_*1*_ and *X*_*6*_) in most settings, especially when the confounders are more strongly associated with the outcome than the treatment. Moreover, AdaLASSO and LASSO methods always choose instrumental variables *X*_*21*_ and *X*_*26*_ in the GPS models of treatment and have a higher possibility of including spurious variables, while the GOAL method instead presents a lower percentage of including instrumental variables and spurious variables, especially when there is no correlation between covariates (ρ = 0).

In the effect estimation stage, the bandwidth diagnostic results for different candidate bandwidths suggest that the *h*_*ROT*_ bandwidth fails to achieve localized smoothing due to its excessively large effective window half-width, which approaches the entire exposure span, consequently precluding its utilization in the simulations. Meanwhile, both *h*_*wp*_ and *h*_*wp us*_ maintain sufficient local effective information and favorable covariate balance and weight stability, characterized by non-extreme effective sample size (ESS), maximum standardized mean difference (SMD) all below 0.25, and maximum weight coefficient of variation (CV) all lower than 1 (Table D in S1 Appendix). Therefore, both bandwidths are adopted in the simulations, with trade-offs made based on the estimation results.

The results in Tables 1–3 and Tables A-C in S1 Appendix separately report the average of the relative absolute bias (Bias%), standard deviation (SD), root mean squared error (RMSE), and coverage probability (CP) for each causal mediation effect over the range of *a* ∈ {-1.0, -0.9,  ..., -0.1, 0.1,  ..., 0.9, 1.0}. The Stability check of variance estimates based on bootstrap resampling indicates that the estimates remain consistent across different levels of replication, supporting the robustness and reliability of our bootstrap-based inference (Table E in S1 Appendix). Due to no interaction effect considered between treatment and mediator, the estimation of causal mediation effects shows an overall consistency under treatment and non-treatment for either NDE or NIE. Notably, we observe slight differences between the estimation bias of NIE under treatment and non-treatment. These discrepancies may be related to random fluctuations arising from different estimation mechanisms and algorithms in NIE estimators under treatment and non-treatment, which have also been reported in previous related literature [5,9]. Moreover, the results of the robustness test on non-linearity and interaction show that the GOAL-based estimator yields similar bias and SD across all the scenarios under data generated by interactive and nonlinear outcome models compared to the original simplified model (Table F in S1 Appendix). This suggests that the proposed method may be relatively robust to moderate violations of the linearity and no-interaction assumptions.

It is notable that estimation based on undersmoothing kernel bandwidth generally maintains a CP of above 90% and closely distributed around the 95% level, while the standard bandwidth cases suffer from unstable coverage of true values due to relatively large bias and narrow confidence intervals. Its superiority is particularly highlighted in covariate correlation settings (*ρ* = 0.3), and remains consistent across different sample sizes and multiple scenarios compared to the standard bandwidth case. The advantage of undersmoothing bandwidth may stem from its strictly faster rate of bias vanishing, which effectively prioritizes bias reduction over variance minimization [31], making this bandwidth option a more preferable strategy to derive more stable and valid inferences. We further examine the comprehensive results of CP performance across the entire exposure spectrum. The results reveal that the minimum coverage probability of the *h*_*wp us*_ bandwidth safely avoids severe under-coverage, and its CP distributions across the exposure range maintain relatively stable around the average with low variation (Table G in S1 Appendix). These findings demonstrate the stability and consistency of our inference framework under appropriate bandwidth specification across the exposure evaluation range, and also support the reliability of the mean-based CP metrics.

From Tables 1–3 and Tables A-C in S1 Appendix, we can observe that both NDE and NIE estimates of the GOAL-based estimators outperform other regularization method-based methods under most scenarios, yielding the smaller relative absolute bias, SD, and RMSE. This result is in line with common perspectives in causal inference literature that incorporating outcome information by including prognostic variables in the propensity score model can improve the efficiency of the estimation [18], while additionally adding non-outcome related variables may increase SD and RMSE [32]. It also aligns with the results of the referenced outcome benchmark GPS models that only include confounders and prognostic variables. In NIE estimation, although the SD and RMSE of our proposed GOAL-based method are generally smaller or close to those of other competing methods, its relative absolute bias remains consistently lower across different scenarios. By contrast, other regularization-based methods experience significantly inflated estimation bias and lower CP of true values with correlated covariates (*ρ* = 0.3) or with a smaller sample size. Similar results can also be observed in the benchmark GPS models that include instrumental variables and exclude outcome-related variables. Notably, the double machine learning method generally exhibits superior performance characterized by higher efficiency and robust estimation in NDE estimation compared to our proposed method. Nevertheless, its reduction in variance is offset by pronounced inflation in estimation bias for NIE estimation, leading to suboptimal coverage of the true values and a substantial decline in inferential validity. Whereas, our method remains consistent in such cases, demonstrating an overall comparative advantage in estimating both NDE and NIE effects, achieving robust estimation without substantially compromising variance. Furthermore, as the sample size increases, our method closely resembles the machine learning approach and similarly demonstrates comparable advantages. Therefore, when compared to the double machine learning method, our method offers a relatively more stable efficiency-bias balance, while also enabling informative covariate identification and noise filtering, thereby enhancing model interpretability and facilitating deeper insights into the underlying mechanisms.

The estimates generally present significantly lower relative bias, SD, and RMSE in the condition of independent covariates than in covariate-correlated settings in most settings for all the regularization-based methods. Both the bias and variance are observed to be significantly reduced when the sample size increases from 2,000–5,000, especially for the NDE estimates. Moreover, as the correlation strength between covariates and the outcome decreases and the correlation with the treatment enhances, i.e., from Scenario 1–3, the proposed method exhibits a relatively increased bias. However, the bias remains smaller than that of competing methods, whilst maintaining high estimation efficiency and valid inference.

The results of the robustness test regarding the method sparsity demonstrate that our proposed method maintains high selection accuracy approaching 100% and yields stable estimations as the proportion of true signals increases (Table H in S1 Appendix and Fig C in S2 Appendix). It justifies the consistency and reliability of our framework in scenarios characterized by weaker sparsity or denser signal structures. Furthermore, the sensitivity analysis exploring a continuous spectrum of covariate-to-sample size (*p*/*n*) ratios shows that the proposed method maintains overall robust performance across low-to-moderate ratios (≤ 0.6). As the ratio escalates to 0.9, a noticeable decline in estimation efficiency emerges, primarily characterized by moderately fluctuating increases in SD and RMSE (Table I in S1 Appendix). This precision loss may stem from the inclusion of spurious noise variables induced by selection degradation in heavily congested covariate space. Nevertheless, by virtue of the sparse true model structure and the identifiability of the initial ordinary least squares regression in adaptive penalty weights construction, no severe breakdown is observed within the *p* < *n* regime. This result suggests that the proposed approach is capable of accommodating moderately large covariate spaces, while also reflecting the potential impact of higher dimensionality on estimation accuracy. In addition, our method demonstrates consistent estimation performance both in scenarios with normally distributed errors and with binary outcomes, generally exhibiting low estimation bias and valid statistical inference (Tables J-K in S1 Appendix), which highlights the robustness of our approach to distributional assumptions and different outcome settings. Despite the misspecified GPS models of treatment *A* given *X* and given (*X*, *M*), the GOAL-based method, in most cases, manifests a better property in balancing bias and efficiency compared to other methods. Its superiority appears more distinct in undersmoothing bandwidth-based estimation, which implies that misspecification of the GPS models may not entail important biases under a sufficiently small kernel bandwidth, as interpreted in the prior work of Huber et al. [5].

To sum up, the simulation results illustrate the superiority of the proposed GOAL-based method in accurately identifying outcome-related covariates and excluding instrumental variables and spurious variables, and in obtaining accurate and valid estimation while improving estimation efficiency. The results imply that a more promising performance of the estimation can be obtained under an appropriate bandwidth strategy based on sample sizes with several thousand observations, which is easily met in practical biomedical studies.

### 2.2 Real data application results

The detailed information of the real-world analysis that explores the mediating role of apolipoprotein (ApoB) levels in the association between Finnish Diabetes Risk Score (FINDRISC)-represented potential diabetes risk and overall cancer incidence is presented in the Method Section and Tables L-O in S1 Appendix.

Our proposed GOAL-based method selects 45 key covariates in both the treatment GPS model given covariates, and the model given the combination of covariates and the mediator. In comparison, the AdaLASSO and LASSO-based methods select 52 and 92 variables, respectively, across both of the aforementioned GPS models. The factors identified by the GOAL method cover a wide range of biologically plausible risk factors, including the demographic and socioeconomic characteristics (e.g., sex, education attainment, income level, etc.), lifestyle, dietary and behavior patterns (e.g., sedentary behavior, tea and coffee intake, etc.), genetic risk factors (standard polygenic risk score for breast cancer, cardiovascular disease, and type 1 diabetes, etc.), mental health problems and stress-related events (physical and psychological abuse in the childhood, etc.), and biochemical and metabolic measures (systolic blood pressure, alanine aminotransferase, triglycerides, etc.). Notably, many of the identified covariates are in concordance with findings from prior research [33–44]. For instance, previous research indicates that certain genes associated with breast cancer risk also participate in diabetes-related symptoms, lipid metabolism-related molecules, and the occurrence of multiple other cancers [45–47]. It provides consistent evidence for our selected variable of genetic risk of breast cancer in the diabetes-cancer pathway. Also, the number of treatments/medications taken is supported by established evidence, especially implying that polypharmacy can both have an impact on diabetes risk and cancer incidence [48,49]. Moreover, some identified urinary-related indicators (e.g., sodium, potassium, and creatinine in urine) represent key physiological states such as blood pressure related to dietary habits, muscle mass, and renal function, and their confounding effects have also been consistently reported in prior findings [50–55]. This consistent evidence confirms the biological plausibility of our variable selection results.

The bandwidth diagnostic checks reveal that the effective window half-widths of the *h*_*wp*_ and *h*_*wp us*_ bandwidths are smaller than the minimum exposure spacing of 1, failing to secure adequate local empirical support for valid estimation (Table P in S1 Appendix). On the other hand, the 1.5*h*_*ROT*_ and 2*h*_*ROT*_ specifications are similarly excluded, as their excessively large effective half-widths approach or even surpass the full exposure spectrum, fundamentally defeating the purpose of localized smoothing. Consequently, diagnostic results suggest that the ideal bandwidth approaches or falls within the range between 0.75*h*_*ROT*_ and 1.25*h*_*ROT*_. Supplementary evaluations across this spectrum further demonstrate generally consistent estimation performance across the exposure levels (Figs D-E in S2 Appendix). Therefore, we adopt the *h*_*ROT*_ bandwidth as a representative specification to obtain robust estimation. As shown in Table 4, our proposed GOAL method achieves relatively high estimation efficiency with lower SD for both direct and indirect effects estimation compared to other regularization-based methods. Also, compared to traditional models including all available covariates, which are more likely to introduce noise from irrelevant factors, our method selection-integrated approach enhances the reliability and precision of clinical inferences by focusing on the most relevant biological predictors. The variability plots (Figs F-G in S2 Appendix) illustrate that the GOAL method applied to our real data application has a solid capacity to identify important and high-frequency variables with a selection proportion ≥ 60% in 100 bootstrap resampling. This result demonstrates the stability and reliability of the variable selection results of the GOAL method in the current empirical analysis.

**Table 4:** The average standard deviations of the estimation based on different variable selection procedures in the real data analysis.

Method	Average Standard Deviation			
	NDE (treatment)	NDE (non-treatment)	NIE (treatment)	NIE (non-treatment)
GOAL	0.013	0.013	0.013	2.33E-03
AdaLASSO	0.017	0.017	0.017	2.57E-03
LASSO	0.018	0.018	0.017	2.60E-03
Full	0.018	0.018	0.018	2.20E-03

**Note**: The average standard deviations are calculated by averaging the standard deviation of the effects across the treatment values *a* ∈ {3, 4,  ..., 13} versus *a’* = 2. NDE (treatment or non-treatment) (same as that for NIE) separately indicates the natural direct effect estimated under *A = a* or the natural direct effect estimated under *A = a’*.

Abbreviations: NDE, natural direct effect; NIE, natural indirect effect; GOAL, general outcome-adaptive LASSO; AdaLASSO, adaptive LASSO.

**Fig 5:**
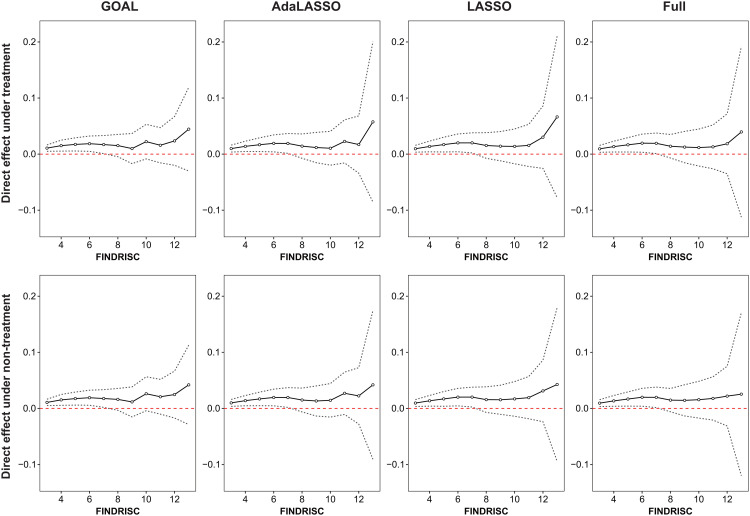
Direct effects ${\hat{\theta }}_{a,2}\left(a\right)$ and ${\hat{\theta }}_{a,2}\left(2\right)$ estimation using the GOAL-based method and the competing methods. **Notes:** The treatment (i.e., FINDRISC) value *a* ∈ {3, 4,  ..., 13}. The horizontal axis represents the continuous treatment value in increments of 2, and the vertical axis denotes the magnitude of the natural direct effect (NDE) under treatment or non-treatment. The solid black line with hollow dots depicts the point estimates of the NDE at varying treatment levels, and the grey dashed lines surrounding the solid line represent the 95% pointwise confidence intervals derived from 500 bootstrap resampling. The horizontal dashed line at zero represents no causal effect observed on the risk difference scale through the direct pathway between the treatment and the outcome. Estimated values above zero suggest a risk effect, while values below zero indicate a protective effect. Abbreviations: LASSO, the least absolute shrinkage and selection operator; GOAL, generalized outcome-adaptive LASSO; AdaLASSO, adaptive LASSO; FINDRISC, Finnish Diabetes Risk Score.

**Fig 6:**
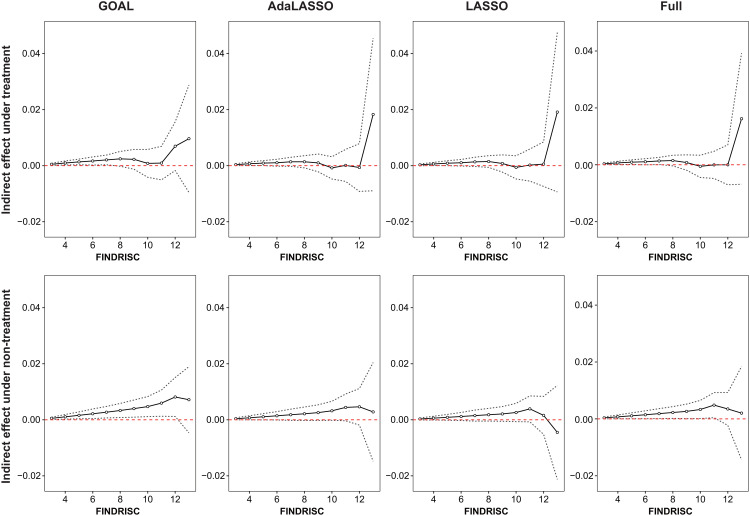
Indirect effects ${\hat{\delta }}_{a,2}\left(a\right)$ and ${\hat{\delta }}_{a,2}\left(2\right)$ estimation using the GOAL-based method and the competing methods. **Notes:** The treatment (i.e., FINDRISC) value *a* ∈ {3, 4,  ..., 13}. The horizontal axis represents the continuous treatment value in increments of 2, and the vertical axis denotes the magnitude of the natural indirect effect (NIE) under treatment or non-treatment. The solid black line with hollow dots depicts the point estimates of the NIE at varying treatment levels, and the grey dashed lines surrounding the solid line represent the 95% pointwise confidence intervals derived from 500 bootstrap resampling. The horizontal dashed line at zero represents no causal effect observed on the risk difference scale through the indirect pathway between the treatment and the outcome. Estimated values above zero suggest a risk effect, while values below zero indicate a protective effect. Abbreviations: LASSO, the least absolute shrinkage and selection operator; GOAL, generalized outcome-adaptive LASSO; AdaLASSO, adaptive LASSO; FINDRISC, Finnish Diabetes Risk Score.

Figs 5 and 6 illustrate the dose-response causal mediation effects of varying levels of potential diabetes risk (from risk score of 3–13) on the overall cancer incidence through the ApoB pathway under different identified factors in both treatment and nontreatment groups. From Fig 5, we can find that the NDE estimates by the GOAL method-based approach show an overall upward trend as the treatment value increases, revealing that the direct effect of diabetes risk on cancer incidence markedly intensifies as the former risk level rises. Also, it exhibits a significant sign in the early stages of the diabetes risk window. This result aligns with previous epidemiological findings that patients with moderate- and high-diabetes risk might suffer an increased risk of overall cancer incidence compared to those with low risk or healthy populations [56,57]. Furthermore, the superiority of the GOAL-based method gets more pronounced at higher diabetes risk levels, where the sample size is smaller, demonstrating its inferential stability and reliability across the entire exposure spectrum.

**Fig 6** presents the indirect effect mediated by the level of ApoB. Similar to the estimation of NDE, our method provides more precise effect estimates than alternative models, especially at extreme risk values. The findings indicate that the indirect impact of ApoB-related biological pathways in the diabetes-cancer link intensifies as diabetes risk rises. This supports the biological plausibility of ApoB as a key mediator, consistent with the established evidence that dysregulation of lipid metabolism involving ApoB may partly link potential diabetes risks to oncogenic processes [58–60]. It also provides a novel perspective on clinical management that targeting ApoB-related lipid metabolism pathways could serve as an effective preventive strategy to prevent cancer development in high-risk diabetic populations.

In the sensitivity analysis to examine the stability of the model through 100 bootstrap resampling, we find that the average outcome incidence is 14.9% (14.0%, 15.8%) and the average SDs for the GOAL-based estimates of causal mediation effects are also similar to those of the original data (Table Q in S1 Appendix). These results indicate a stable outcome distribution under bootstrapping and support the estimation performance consistency and model stability of our method. Additionally, the estimation results of the sensitivity analysis adopting different bandwidths show that the proposed GOAL-based method consistently produces the smallest average SD among other alternative methods, demonstrating the highest estimation efficiency (Table R in S1 Appendix). Furthermore, the estimated direct and indirect effects remained stable across these bandwidth settings, with no substantial changes observed. (Figs H-I in S2 Appendix) These results suggest that our kernel-based estimation procedure is relatively robust to moderate bandwidth variation.

In summary, the mediation analysis results indicate that our proposed GOAL-based method is able to identify risk factors with evidence-based biological plausibility from large-dimensional covariates with higher efficiency of estimation. Moreover, our investigation provides a novel perspective that the elevated underlying diabetes risk may not only have a direct effect on the increase of the risk of overall cancer incidence, but also exerts a significant indirect effect through ApoB-related metabolic pathways. Clinical strategies and health management based on ApoB-related metabolic pathways may be considered as indirect intervention targets between potential diabetes risk and cancer occurrence in the future. Further exploration into more potential mediating pathways and more complicated modeling forms can be undertaken in the future to provide a comprehensive understanding of the mechanisms involved.

## 3 Discussion

In this study, we propose a method that integrates the GOAL method and GPS-weighted semiparametric estimation to identify the natural direct and indirect effects. The proposed method provides a possible solution to the ‘curse of dimensionality’ in estimating causal mediation effects for continuous treatment and mediators under large-scale covariate spaces that exceed the scope of conventional methods and typical straightforward practice. Also, our method achieves estimation efficiency by precisely incorporating outcome information. We further apply the proposed method to a practical study with large-scale biomedical data and illustrate its superiority in variable selection and estimation efficiency.

From the simulation studies, we find that the proposed method generally yields more efficient estimates with relatively smaller SD and RMSE compared to other regularization-based methods. It is realized by precisely including outcome-related covariates and excluding instrumental variables and spurious variables in the propensity score models. The superiority of our method becomes more evident in settings when adopting the undersmoothing kernel bandwidth, demonstrating a significantly lower bias and higher coverage probability of true values. As the correlation between covariates reduces and the sample size increases, the performance of the proposed method generally shows a profound increase, especially for NDE. Comparisons with the double machine learning method also demonstrate the overall superiority in estimation and the robust performance of our method across various scenarios.

In the practical data analysis, we apply the proposed method to investigate the ApoB-related causal pathway between FINDRISC-represented potential diabetes risk and overall cancer incidence with large-scale biomedical data. We select a subset of variables from the large covariate space with realistic interpretability for the propensity score models. Based on the selected variable subset, the results show that FINDRISC is both significantly directly associated with cancer occurrence and indirectly associated via the ApoB-mediated pathways over a part of the treatment value range. These results are aligned with previous findings [56,57] and are supported by established biological mechanisms of the underlying function of ApoB-related lipid metabolism in the link between diabetes and cancer [58–60]. These findings provide novel clues based on characteristics of underlying risk populations for clinical intervention and population-wide prevention strategies of cancer. Potential clinical interventions based on ApoB may include using ApoB as a novel biomarker to screen tumorigenesis [61], measuring the ApoB to ApoA-I ratio to predict cancer progression and prognosis [62], utilizing ApoB to construct a delivery carrier for epigenetic drugs to achieve local ablation treatment for cancers, etc. [63] Also, population-wide hazard control and prevention strategies related to ApoB levels may involve reducing smoking and alcohol consumption, implementing weight management, adopting a healthy diet, and so on [64,65]. In addition to its application in the investigation of clinical mechanisms under intricate factors, the proposed method can also be extended to other large-dimensional settings, such as diverse omics data and medical image data.

The article demonstrates the proposed methodology in the case of continuous mediators, but it can also be naturally extended to binary mediators. Our method is generally introduced under a simplified linear and non-interactive outcome model, and we further provide a robustness test under assumption violations in consideration of common practical applications. Nonetheless, further research is needed to explore more complex model structures or broader data types in the estimation framework to accommodate intricate biological mechanisms. Although the influence of misspecified GPS models may not entail important bias under a small kernel bandwidth, further applications of robust methods in the propensity scores to address the challenge of model misspecification might be a promising direction for future investigation. Also, since our proposed estimator is sensitive to different kernel bandwidth specifications, we implement a sequence of diagnostic steps to examine their performance in terms of local information support, covariate balance, and weight robustness. Nevertheless, due to the lack of definitive decision-making boundaries within these diagnostics, further theoretical development and practical exploration targeted at refining bandwidth specification remain highly warranted. Given that our estimation method involves dual steps of variable selection and effect estimation, statistical inference based on the property of the complicated estimator poses great challenges [66,67]. Therefore, we adopt the bootstrap method for inference. Although bootstrapping regularization methods (such as LASSO) may be subject to finite-sample bias, we evaluate the validity and robustness of the inference by calculating the coverage probability of true values, and our method demonstrates favorable inference capabilities under an appropriate bandwidth strategy. Nevertheless, future development of a direct, computationally efficient inference theory remains an important question and would be a worthwhile endeavor. Additionally, a comparison of our method with a non-regularization machine learning benchmark method shows that, although the competing method offers higher estimation efficiency, it narrowly constrained confidence intervals may lead to an increase in estimation bias. Therefore, our approach may serve as a more preferable option when considering the trade-off between efficiency and bias, as well as for the purposes of filtering high-dimensional information and enhancing model interpretability. Still, other modern machine learning techniques, Bayesian mediation, and causal forests remain to be explored [24,25,27–30,68]. Furthermore, learning directed acyclic graphs (DAGs) methods represent another important branch of techniques for identifying causal structures in large-dimensional settings [69–72], and their integration with causal inference frameworks may offer promising directions for mediation analysis. Further attempts and expanded applications based on broader advanced methodologies are also worth pursuing in the future. While our approach is currently applicable to scenarios where covariate dimensionality is strictly smaller than the sample size (*p* < *n*), further methodological modifications and empirical implementations for high-dimensional settings where *p* exceeds *n* warrant future investigation. It is also worth noting that the continuous exposure used in our empirical analysis is a composite score variable, and the quasi-discrete nature of the exposure may limit the elaboration of the estimation performance of our proposed method for fully continuous variables. Therefore, further attempts can be made to explore applications involving smoother continuous treatments, such as biochemical measurements or environmental exposures, to better leverage the flexibility of the proposed framework. Further, due to the higher participation tendency of healthier and more socioeconomically advantaged participants, the UK Biobank cohort is not fully representative of the general population. As such, the generalizability of our findings may be limited, and future studies based on other populations or external validation cohorts are warranted to assess robustness and transportability.

## 4 Method

### 4.1 Ethics statement

The real-world data used in empirical analysis were obtained from the UK Biobank, which has received ethical approval from the North West Multi-Centre Research Ethics Committee to function as a Research Tissue Bank (RTB). During the recruitment stage, written informed consent has been obtained from all participants. The RTB approval allows researchers to operate under this approval with no need for further ethical approval, other than exceptions such as re-contact applications. The approval was granted initially in 2011 and is renewed on a five-yearly cycle, with the most recent renewal approved in 2021 (https://www.ukbiobank.ac.uk/wp-content/uploads/2025/01/Ethics-approval-renewal-2021.pdf).

### 4.2 Notation and reviews of the semiparametric estimation approach

Our goal is to disentangle the average treatment effect (ATE) of a continuous treatment variable *A* on an outcome variable *Y* into a direct effect and an indirect effect through a mediator *M*, which can be either discrete or continuous (hereafter, we use the continuous mediator to elaborate the methodology). Suppose the observed data (*A*_*i*_, *M*_*i*_, *Y*_*i*_, *X*_*i*_), *i* = 1, 2,  ..., *n*, are identically distributed and independent samples drawn from a common joint distribution *f(A, M, Y, X)*, where $X_{i}\in R^{p}$ represent the *p*-dimensional pre-treatment covariates. For generic random variables *A* and *M*, A and M denote the continuous support of *A* and *M*, respectively*.* For the definition of causal mediation effects, we apply the potential outcome framework [73] in the context of mediation analysis as described in previous literature [74–76]. Under treatment values $a,\;a^{\prime }\in A$, *M*(*a*) is defined as a function of the treatment, denoting the potential mediator state that would have been observed when the treatment level is set to be *a*; and *Y*(*a*, *M*(*a*’)) is defined as a function of the treatment and the potential mediator, denoting the potential outcome that would have been observed with the treatment set to *a* and the mediator set to the value it would attain under treatment *a’*. The mean potential outcomes are then defined as μ(a,a)=E[Y(a,M(a))] and $\mu \left(a,a^{\prime }\right)=E\left[Y\left(a,M\left(a^{\prime }\right)\right)\right]$ with *a ≠ a’*. μ(a, a) has also been stated as the average dose-response function for a continuous treatment in the literature [13,14].

Under this notation, the ATE of treatment value *a* versus *a’*, denoted by ${△}_{a,a^{\prime }}$, can be written as


$$\begin{array}{c} {△}_{a,a^{\prime }}=\mu (a,a)-\mu \left(a^{\prime },a^{\prime }\right),\;for\;a\neq a^{\prime }\text{.} \end{array}$$ (1)

The ATE can be interpreted as the total average effects of *A* on *Y* from both the direct pathway and the indirect pathway via potential mediators *M*(*a*) and *M*(*a’*). The average natural direct effect (NDE) is defined as the average outcome difference between *a* and *a’* when keeping the potential mediator fixed at the level of *M*(*a*) or *M*(*a’*):


$$\begin{array}{c} \theta_{a,a^{\prime }}(a)=\mu (a,a)-\mu \left(a^{\prime },a\right),\;\;\theta_{a,a^{\prime }}\left(a^{\prime }\right)=\mu \left(a,a^{\prime }\right)-\mu \left(a^{\prime },a^{\prime }\right),\;\;for\;\;a\neq a^{\prime }\text{.} \end{array}$$ (2)

Analogously, the average natural indirect effect (NIE) is the average outcome difference between *M*(*a*) and *M*(*a’*) when keeping the treatment fixed at *a* or *a’*:


$$\begin{array}{c} \delta_{a,\;a^{\prime }}(a)=\;\mu (a,\;a)-\mu \left(a,\;a^{\prime }\right),\;\delta_{a,\;a^{\prime }}\left(a^{\prime }\right)=\mu \left(a^{\prime },a\right)-\mu \left(a^{\prime },a^{\prime }\right),\;for\;a\neq a^{\prime }\text{.} \end{array}$$ (3)

By simply performing addition or subtraction between Equations 2 and 3, it can be readily drawn that ATE equals the sum of NDE and NIE, i.e., $\begin{array}{c} {△}_{a,a^{\prime }}=\theta_{a,a^{\prime }}(a)+\delta_{a,\;a^{\prime }}\left(a^{\prime }\right)=\theta_{a,a^{\prime }}\left(a^{\prime }\right)+\delta_{a,\;a^{\prime }}(a) \end{array}$. In fact, $\theta_{a,a^{\prime }}(a)$ and $\delta_{a,\;a^{\prime }}(a)$ can be respectively different from $\theta_{a,a^{\prime }}\left(a^{\prime }\right)$ and $\delta_{a,\;a^{\prime }}\left(a^{\prime }\right)$ if the NDE and NIE are heterogeneous in *A* and *M*, which indicates the presence of interactions between *A* and *M*. The identification of the direct and indirect mediation effects is based on the assumptions of consistency, positivity, and conditional independence (Assumptions 1–3, Text A in S3 Appendix).

In this paper, we will follow the GPS-weighted semiparametric estimation approach proposed by Huber et al. to estimate the causal mediation effects for continuous treatment and mediator [5]. This method is built upon the weights constructed by the inverse of two versions of the GPSs, separately corresponding to the conditional treatment densities given observed covariates and given covariates and the mediator. Due to the probability of a specific value *a* being equal to zero for the continuous treatment *A*, we use kernel density instead of indicator function 1(*A = a*) (adopted in the binary treatment context) [4] to modify the weighting function of the estimator, which is defined as ω(A;a, h)≡K((A−a)/h)/h, where *K* is a symmetric second-order kernel function and *h* is a bandwidth. The weighting function depends on the distance between *A* and the reference value *a* and a nonnegative tuning parameter *h*, where larger discrepancies between *A* and *a* gain less weight as *h* approaches zero.

Let $f_{A}(a|X)$ and $f_{A}(a|M,\;X)$ be the GPS models separately corresponding to $lim_{h\to \text{0}}E[\omega (A;a^{\prime },\;h)|X]$ and $lim_{h\to \text{0}}E[\omega (A;a^{\prime },\;h)|M,X]$. Under the assumption that $f_{A}(a|M,\;X)$ and E [Y|A=a, M, X] are continuous in *a*, the inverse-GPS weighted mean potential outcomes μ(a,a) and $\mu \left(a,\;a^{\prime }\right)$ with *a ≠ a’* can be written as


$$\begin{array}{c} \mu (a,a)=\underset{h\to \text{0}}{\text{lim}}E\left[\frac{Y\cdot \omega (A;a,h)}{f_{A}(a|X)}\right] \end{array}$$ (5)

$$\begin{array}{c} \mu \left(a,a^{\prime }\right)=\underset{h\to \text{0}}{\text{lim}}E\left[\frac{Y\cdot \omega (A;a,h)}{f_{A}(a|M,X)}\cdot \frac{f_{A}(a^{\prime }|M,X)}{f_{A}(a^{\prime }|X)}\right] \end{array}$$ (6)

as *h* goes to zero.

For an s-dimensional vector *u* = *u* (*u*_(1)_,  ..., *u*_(s)_)*’*, let $K_{h}(u)=\prod_{l=\text{1}}^{s}k(u_{\left(l\right)}/h)/h$ be a product kernel with a generic kernel function k and bandwidth *h* for estimating the mean potential outcomes. To parametrically specify the GPS models $f_{A}(a|X)$ and $f_{A}(a|M,\;X)$ in the first step of the weighted semiparametric estimation of μ(a,a) and $\mu \left(a,a^{\prime }\right)$, we also invoked Assumption 4 (Text A in S3 Appendix) for parametric generalized propensity scores.

The sufficient conditions for Assumption 4 have been illustrated by Huber et al.[5] The estimator ${\hat{\gamma }}_{x}$ of the GPS model $f_{A}\left(a|x;\;\gamma_{x}\right)$, $\gamma_{x}\;\in \;\Gamma_{x}\;\subseteq R^{S_{x}}$, satisfies ${sup}_{x\in X}\left|f_{A}\left(a|x;\;{\hat{\gamma }}_{x}\right)-f_{A}\left(a|x;\;\gamma_{x\text{0}}\right)\right|=O_{p}(n^{-\text{1}/\text{2}})$, where $\gamma_{x\text{0}}\in \;\Gamma_{x}$ such that $f_{A}\left(a|x\right)=f_{A}\left(a|x;\;\gamma_{x\text{0}}\right)$ for all x∈X. Similarly, the estimator ${\hat{\gamma }}_{mx}$ of the GPS model $f_{A}\left(a|m,x;\;\gamma_{mx}\right)$, $\gamma_{mx}\;\in \;\Gamma_{mx}\;\subseteq R^{S_{x}m}$, satisfies ${sup}_{m\in M,\;x\in X}\left|f_{A}\left(a|m,x;\;{\hat{\gamma }}_{mx}\right)-f_{A}\left(a|m,x;\;\gamma_{mx\text{0}}\right)\right|=O_{p}(n^{-\text{1}/\text{2}})$, where $\gamma_{mx\text{0}}\in \;\Gamma_{mx}$ such that $f_{A}\left(a|m,x\right)=f_{A}\left(a|m,x;\;\gamma_{mx\text{0}}\right)$ for all m∈M and x∈X. The estimator ${\hat{\gamma }}_{x}$ and ${\hat{\gamma }}_{mx}$ are the root-n consistent estimators for $\gamma_{\text{0}x}$ and $\gamma_{mx\text{0}}$ (typically based on maximum likelihood), respectively. Let $f_{X}(x)$, $f_{AX}(a,x)$, $f_{MX}(m,x)$ be the marginal density function and $f_{AMX}(a,m,x)$ be the joint density function. Then, $f_{A}\left(a|x\right)=f_{AX}(a,x)/f_{X}(x)$ and $f_{A}\left(a|m,x\right)=f_{AMX}(a,m,x)/f_{MX}(m,x)$ can be consistently estimated by $f_{A}\left(a|x;\;\hat{\gamma }\right)=f_{AX}(a,x;\;\hat{\gamma })/f_{X}(x;\;\hat{\gamma })$ and $f_{A}\left(a|m,x;\;\hat{\gamma }\right)=f_{AMX}(a,m,x;\;\hat{\gamma })/f_{MX}(m,x;\;\hat{\gamma })$, respectively. Then, semiparametric estimators for μ(a,a) and $\mu \left(a,a^{\prime }\right)$ can be written as


$$\begin{array}{c} \hat{\mu }(a,a)=\sum_{i=\text{1}}^{n}\frac{Y_{i}K_{h}(A_{i}-a)}{\hat{f_{A}}(a|X_{i};\;{\hat{\gamma }}_{x})}/\sum_{i=\text{1}}^{n}\frac{K_{h}(A_{i}-a)}{\hat{f_{A}}(a|X_{i};\;{\hat{\gamma }}_{x})}\text{,} \end{array}$$

$$\begin{array}{c} \hat{\mu }\left(a,a^{\prime }\right)=\sum_{i=\text{1}}^{n}\frac{Y_{i}K_{h}(A_{i}-a)}{\hat{f_{A}}(a|M_{i},\;X_{i};\;{\hat{\gamma }}_{mx})}\cdot \frac{\hat{f_{A}}(a^{\prime }|M_{i},\;X_{i};\;{\hat{\gamma }}_{mx})}{\hat{f_{A}}(a^{\prime }|\;X_{i};\;{\hat{\gamma }}_{x})}/\sum_{i=\text{1}}^{n}\frac{K_{h}(A_{i}-a)}{\hat{f_{A}}(a|M_{i},\;X_{i};\;{\hat{\gamma }}_{mx})}\cdot \frac{\hat{f_{A}}(a^{\prime }|M_{i},\;X_{i};\;{\hat{\gamma }}_{mx})}{\hat{f_{A}}(a^{\prime }|\;X_{i};\;{\hat{\gamma }}_{x})} \end{array}$$ (7)

Assumption 5 invokes several regularity conditions required for the consistency and asymptotic normality of the proposed estimator (Text A in S3 Appendix).

We employ the bootstrap method to non-parametrically estimate variance [77], which is achieved by replacing the random sample $\{(Y_{i},M_{i},D_{i},X_{i}){\}}_{i=\text{1},\ldots ,n}$ with the bootstrap sample ${\left\{\left(\overset{*}{\underset{i}{Y}},\overset{*}{\underset{i}{M}},\overset{*}{\underset{i}{D}},\overset{*}{\underset{i}{X}}\right)\right\}}_{i=\text{1},\ldots ,n}$ and substituting the population distribution with the empirical distribution [5]. The detailed explanation is provided in the next subsection ‘Generalized outcome-adaptive LASSO for mediation analyses’.

The algorithmic overview of the semi-parametric GPS-weighted mediation effect estimation is presented as **Algorithm 1**:

**Algorithm 1** Semi-parametric GPS-weighted mediation effect estimation

1. Input: The outcome value *y*, treatment value *a*, mediator value *m*, and the specified covariates ***x***_***ax***_ and ***x***_***mx***_ for two GPS models;

2. Fit assumed working models of treatment GPS $A\;|\;X_{ax}\;\sim \;N\left(\mu \left(X_{ax}\right),\;\sigma^{\text{2}}\right)$, where $\mu \left(X_{ax}\right)\;=\;{x_{ax}}^{⊤}\;\gamma_{x},$ and $A\;|\;X_{mx}\;\sim \;N\left(\mu \left(X_{mx}\right),\;\sigma^{\text{2}}\right)$, where $\mu \left(X_{mx}\right)\;=\;{x_{mx}}^{⊤}\;\gamma_{mx}$, and obtained the treatment density $f_{A}\left(a|x;\;\gamma_{x}\right)$ and $f_{A}\left(a|m,x;\;\gamma_{mx}\right)$;

3. For each individual *i*, compute kernel weights $K_{h}(A_{i}-a)$ using an Epanechnikov kernel centered at *a* or *a’* with bandwidth *h*;

4. Compute the kernel-based inverse-GPS weighted $\hat{\mu }(a,a)$ and $\hat{\mu }\left(a,a^{\prime }\right)$ (Equation 7);

5. Calculate the causal mediation effects $\theta_{a,a^{\prime }}(a),\;\theta_{a,a^{\prime }}\left(a^{\prime }\right),\;\delta_{a,\;a^{\prime }}(a),\;\delta_{a,\;a^{\prime }}\left(a^{\prime }\right)$;

6. Use the bootstrap method to obtain the estimated standard error (SE) and p-value;

7. Output: $\theta_{a,a^{\prime }}(a),\;\theta_{a,a^{\prime }}\left(a^{\prime }\right),\;\delta_{a,\;a^{\prime }}(a),\;\delta_{a,\;a^{\prime }}\left(a^{\prime }\right)$ estimates, SE and p-value.

### 4.3 Generalized outcome-adaptive LASSO for mediation analyses

To precisely specify the covariate sets for the GPS models $f_{A}\left(a|x;\;{\hat{\gamma }}_{x}\right)$ and $f_{A}\left(a|m,x;\;{\hat{\gamma }}_{mx}\right)$ of the weighted semiparametric estimation for continuous treatment, we utilized the GOAL method [22] to select important variables to eliminate the confounding effect and improve estimation efficiency, which shrinks the coefficients of the outcome-unrelated covariates to zero.

Let *X*_*C*_ denote confounders that have an impact on treatment, outcome, and mediator. Let *X*_*P*_ denote prognostic covariates that only predict the outcome and are unrelated to the mediator and treatment. Let *X*_*I*_ denote instrumental covariates that only predict the treatment but have no association with the mediator and outcome. Let *X*_*S*_ denote spurious covariates that are unrelated to treatment, outcome, or mediator. To avoid the confounding bias and increase the statistical efficiency, we shall include all *X*_*C*_ and *X*_*P*_ and exclude *X*_*I*_ and *X*_*S*_ in the GPS models.

The corresponding GOAL estimates ${\hat{\gamma }}_{x}$ and ${\hat{\gamma }}_{mx}$ are defined as


$$\begin{array}{c} {\hat{\gamma }}_{x}=arg\underset{\gamma_{x}}{\text{min}}\;\;||A-\sum_{j=\text{1}}^{p}X_{j}\;{{\gamma_{x}}_{j}||}^{\text{2}}+\lambda_{x}\sum_{j=\text{1}}^{p}{\hat{\omega }}_{j}\left|{\gamma_{x}}_{j}\right|\; \end{array}$$ (8)

and


$$\begin{array}{c} {\hat{\gamma }}_{mx}=arg\underset{\gamma_{mx}}{\text{min}}\;\;\;||A-\sum_{j=\text{1}}^{p}X_{j}{\gamma_{mx}}_{j}-\zeta M\;{||}^{\text{2}}+\lambda_{mx}\sum_{j=\text{1}}^{p}{\hat{\omega }}_{j}\left|{\gamma_{mx}}_{j}\right| \end{array}$$ (9)

where *λ*_*x*_ and *λ*_*mx*_ (uniformly denoted by *λ*) separately represent the nonnegative tuning parameters in the GOAL estimates for two GPS models, ${\hat{\omega }}_{j}={\left|{\tilde{\beta }}_{j}\right|}^{-\tau }$ with the power parameter *τ* > 1, ${\tilde{\beta }}_{j}$ is the unpenalized coefficient of the *j*th covariate in the ‘full’ generalized linear outcome regression model and can be estimated by ordinary least squares: $\left(\tilde{\alpha },\tilde{\beta },\;\tilde{\eta }\right)=arg\underset{\alpha ,\;\beta ,\eta }{\text{min}}\;{\left\|Y-\alpha A-\eta M-\sum_{j=\text{1}}^{p}X_{j}\beta_{j}\right\|}^{\text{2}}$. ${\left||\cdot |\right|}^{\text{2}}$ represents the computation of the squared sum. Under the conditions that λ/$\sqrt{n}\to \text{0}$ and $\lambda \cdot n^{\tau /\text{2}-\text{1}}\to \infty$ for *τ* > 1, GOAL imposes heavier weights on covariates that are less associated with the outcome given treatment and mediator (i.e., *X*_*I*_ and *X*_*S*_), such that these covariates tend to be excluded in the GPS models [21]. Notably, for binary outcomes, the unpenalized coefficients can be obtained via maximum likelihood estimation under a logistic regression model. The corresponding algorithmic details are provided in Text B in S3 Appendix.

Given the complexity of the final multi-stage effect estimator involving large-scale variable selection, statistical inference based on asymptotic linear properties (e.g., influence function-based inference) may become invalid [66,67,78]. We therefore adopt the nonparametric bootstrap method to approximate the sampling distribution and estimate variance.

We summarize the above estimation procedure in Algorithm 2:

**Algorithm 2** Generalized outcome-adaptive LASSO estimation

1. Input: The sample size *n*, the target data with (*Y*, *A*, *M*, ***X***), and the pre-specified set of tuning parameter *λ*;

2. Fit the full generalized linear outcome model g(*Y*)~*A* + *M* + ***X***, and obtain the coefficients ***β*** of the covariates ***X***;

3. Choose the optimized *λ*_*x*_ and *λ*_*mx*_ that minimize the deviance with 10-fold cross-validation for two GPS models;

4. Obtain the LASSO estimates ${\hat{\gamma }}_{x}$ and ${\hat{\gamma }}_{mx}$ (Equations 8 and 9) using coefficients ***β***-weighted penalty under optimized *λ*_*x*_ and *λ*_*mx*_.

5. Select covariates with non-zero coefficients in two GPS models for sequential estimation.

6. Output: optimized *λ*_*x*_ and *λ*_*mx*_ and selected covariates for two GPS models.

### 4.4 Simulation setup

We conduct a simulation study to estimate the finite sample properties of the proposed method. To ensure ${\theta_{a,a^{\prime }}(a)=\theta }_{a,a^{\prime }}\left(a^{\prime }\right)$ and $\delta_{a,\;a^{\prime }}(a)=\;\delta_{a,\;a^{\prime }}\left(a^{\prime }\right)$ hold in Equations 2 and 3, we assume that there is no interaction between the treatment and mediator and that the associations between outcome and treatment, mediator, or covariates are linear. Referring to Huber et al.[5], we adopt the Gaussian assumption in the algorithm as a quasi-likelihood framework to parametrically estimate two treatment GPS models conditioned on covariates and/or the mediator. To define the NDE and NIE, we set *a’* = 0 and let *a* take a series of grid points between (and including) -1 and 1 with a step size of 0.1 but excluding 0, defined by $a\in \;G_{r}:=\{-\text{1}.\text{0},\;-\text{0}.\text{9},\;\ldots ,\;-\text{0}.\text{1},\;\text{0}.\text{1},\;\ldots ,\;\text{0}.\text{9},\;\text{1}.\text{0}\}$, where $G_{r}$ is a set of the grid points. Considering the optimal efficiency in symmetric second-order kernels [79,80] and the requirements for valid semiparametric inference, we utilize second-order Epanechnikov kernels and adopt an undersmoothing bandwidth strategy that can efficiently suppress asymptotic bias and ensure the root-*n* consistency and inferential validity of our estimator. Specifically, we compare the estimation under two bandwidth *h* settings: a small bandwidth (*h*_*wp*_) *C*·*n*^-0.25^ with *C* = 2.34, and a further undersmoothing version (*h*_*wp us*_) set at half of *h*_*wp*_ bandwidth, following the empirical recommendations of Huber et al [5]. In addition, we supplement relevant theories regarding bandwidth specifications in Text C in S3 Appendix, and conduct a series of diagnostics and examinations on three candidate bandwidths: *h*_*wp*_, *h*_*wp us*_, and the rule-of-thumb (ROT) bandwidth *h*_*ROT*_ that is set to *C*·sd(*A*)·*n*^-1/5^.

We repeat 100 times of simulations for each setting and consider two combinations (*n*, *p*) of sample size (*n*) and covariate dimension (*p*): (2000, 100) and (5000, 200). Within each simulation run, the standard error of the proposed estimator is estimated using a non-parametric bootstrap procedure based on 500 replicates. Average relative absolute bias (Bias%), standard error (SD), and root mean squared error (RMSE) are measured to evaluate the performance of the estimators across all the treatment values $a\in G_{r}$. Specifically, the average relative absolute bias was calculated as $|{G_{r}|}^{-\text{1}}\sum_{a\in G_{r}}\left|(\frac{\text{1}}{N_{sim}}\sum_{i=\text{1}}^{N_{sim}}{\hat{\mu }}_{i}(a))-\mu (a)|/\mu (a\right)$, and the average RMSE = $|{G_{r}|}^{-\text{1}}\sum_{a\in G_{r}}\sqrt{\frac{\text{1}}{N_{sim}}\sum_{i=\text{1}}^{N_{sim}}{\left\{{\hat{\mu }}_{i}(a)-\mu (a)\right\}}^{\text{2}}}$, where $\left|G_{r}\right|$ denotes the number of elements in the set $G_{r}$, ${\hat{\mu }}_{i}(a)$ denotes the estimate of the function $\hat{\mu }(a)$ for $a\in G_{r}$ of the *i*th simulation, μ(a) is the true effect at level a, and $N_{sim}$ denotes the number of simulation iterations. Also, as the bootstrap approach does not account for the impact of systematic estimation bias, we therefore evaluate the coverage probability of the bootstrap confidence intervals to assess the inference validity. To further validate the stability of the bootstrap estimates of variance in our simulations, we additionally conduct a sensitivity analysis with a representative simulated dataset using the bootstrap replicates of 200 and 1000 for comparison.

Of all covariates, *X*_*1*_ and *X*_*6*_ are true confounders (*X*_*C*_), *X*_*11*_ and *X*_*16*_ are prognostic variables (*X*_*P*_), *X*_*21*_ and *X*_*26*_ are instrumental variables (*X*_*I*_), and all the others are deemed as spurious covariates (*X*_*S*_). We denote the covariance of covariates as $\sum_{ij}=\rho \;(i\neq j)$ and $\sum_{ij}=\text{1}\;(i=j)$ and generate pre-treatment covariates ***X*** from a uniform distribution (-1, 1) with two covariate correlation patterns, where *ρ* = 0 indicates an independent covariate pattern, and *ρ* = 0.3 indicates all the covariates are correlated with a coefficient of 0.3. Treatment *A* is a linear function of the observed variables *X* and an unobserved *W*, mediator *M* is a linear function of the *A*, *X* and an unobserved *V*, and outcome *Y* is a linear function of *A*, *M*, *X,* and an unobserved *U*. The unobserved variables *U*, *W*, *V* are independent of each other and all follow uniform distributions (-2, 2). Here, we focus our simulation on continuous-outcome settings, which allows for leveraging richer information compared to binary cases and providing a more rigorous assessment of method performance. However, it is worth noting that the variable selection mechanism of our proposed method consistently operates under the general generalized linear model framework (including both linear and logistic models) [20].

Then the data-generating processes for *A*, *M*, and *Y* are as follows:


$$\begin{array}{c} A=\sum_{j=\text{1}}^{p}\gamma_{j}X_{j}+W,\;W\;\sim \;uniform\;(-\text{2},\;\text{2}) \end{array}$$

$$\begin{array}{c} M=\text{0}.\text{3}A+\text{0}.\text{3}(X_{\text{1}}+X_{\text{6}})+V,\;V\;\sim \;uniform\;(-\text{2},\;\text{2}) \end{array}$$

$$\begin{array}{c} Y=A+\text{0}.\text{3}M+\sum_{j=\text{1}}^{p}\beta_{j}X_{j}+U,\;U\;\sim \;uniform\;(-\text{2},\;\text{2})\text{.} \end{array}$$

In our simulation design, the direct effects are defined as ${\theta_{a,a^{\prime }}(a)=\theta }_{a,a^{\prime }}\left(a^{\prime }\right)=\text{1}(a-a^{\prime })=a-a^{\prime }$, and the indirect effects are defined as $\delta_{a,\;a^{\prime }}(a)=\;\delta_{a,\;a^{\prime }}\left(a^{\prime }\right)=\text{0}.\text{0}\text{9}(a-a^{\prime })$.

Three scenarios are then framed to assess the estimation performance of different strengths of confounder-outcome and confounder-treatment association: 1) confounders are strongly associated with both outcome and treatment (SoSt); 2) confounders are more strongly associated with outcome than with treatment (SoWt); 3) confounders are more strongly associated with treatment than with outcome (WoSt).

**Scenario 1 (SoSt)**

In the case that *X*_*C*_ (*X*_*1*_ and *X*_*6*_) are strongly associated with both Y and A, the coefficients of X in the aforementioned function of *A* and *Y*, that is, *γ* and *β*, are as follows:


$$\begin{array}{c} \gamma =(0.3,\underset{\text{four}}{\underbrace{0,\ldots ,0}},0.3,\underset{\text{fourteen}}{\underbrace{0,\ldots ,0}},1,\underset{\text{four}}{\underbrace{0,\ldots ,0}},1,0,0,\ldots ,0) \end{array}$$

$$\begin{array}{c} \beta =(0.3,\underset{\text{four}}{\underbrace{0,\cdots ,0}},0.3,\underset{\text{four}}{\underbrace{0,\cdots ,0}},0.3,\underset{\text{four}}{\underbrace{0,\cdots ,0}},0.3,0,0,\cdots ,0) \end{array}$$

**Scenario 2 (SoWt)**

In Scenario 2, *X*_*C*_ are more strongly associated with *Y* than with *A*. The coefficients *γ* and *β* are


$$\begin{array}{c} \gamma =(0.15,\underset{\text{four}}{\underbrace{0,\cdots ,0}},0.15,\underset{\text{fourteen}}{\underbrace{0,,\cdots ,0}},1,\underset{\text{four}}{\underbrace{0,,\cdots ,0}},1,0,0,,\cdots ,0) \end{array}$$

$$\begin{array}{c} \beta =(0.3,\underset{\text{four}}{\underbrace{0,\cdots ,0}},0.3,\underset{\text{four}}{\underbrace{0,\cdots ,0}},0.3,\underset{\text{four}}{\underbrace{0,\cdots ,0}},0.3,0,0,\cdots ,0) \end{array}$$

**Scenario 3 (WoSt)**

$$\begin{array}{c} \gamma =(0.3,\underset{\text{four}}{\underbrace{0,\ldots ,0}},0.3,\underset{\text{fourteen}}{\underbrace{0,\ldots ,0}},1,\underset{\text{four}}{\underbrace{0,\ldots ,0}},1,0,0,\ldots ,0) \end{array}$$

$$\begin{array}{c} \beta =(0.15,\underset{\text{four}}{\underbrace{0,\cdots ,0}},0.15,\underset{\text{four}}{\underbrace{0,\cdots ,0}},0.3,\underset{\text{four}}{\underbrace{0,\cdots ,0}},0.3,0,0,\cdots ,0) \end{array}$$

The consistency of variable selection can be influenced by two nuisance parameters *λ* and *τ*. In each scenario, we consider *λ* from a set of eight values, the same as those used by Shortreed et al.[21]: {$n^{-\text{1}\text{0}},\;n^{-\text{5}},\;n^{-\text{1}},\;n^{-\text{0}.\text{7}\text{5}},\;n^{-\text{0}.\text{5}},\;n^{-\text{0}.\text{2}\text{5}},\;n^{\text{0}.\text{2}\text{5}},\;n^{\text{0}.\text{4}\text{9}}$} and determine the optimal *λ* by minimizing deviance with 10-fold cross-validation. Also, following the recommendation by Gao et al.[22], we set $\lambda \cdot n^{\tau /\text{2}-\text{1}}=n^{\text{2}}$, where the power parameter of *n* (i.e., the value of 2) is called the convergence factor.

To examine the sensitivity of the proposed method to the distributional assumption of the error terms, we introduce an additional simulation scenario in which the error terms for the exposure, mediator, and outcome models were generated from normal distributions (Text D in S3 Appendix). In addition, we perform a robustness check to examine the estimation performance of the proposed GOAL-based method under interactive and nonlinear outcome model settings. In this sensitivity analysis, we separately assess the estimation performance of our method under an outcome generation model incorporated with a treatment–mediator interaction term and under a nonlinear outcome model with the interaction term and the quadratic terms in both the treatment and the mediator (Text E in S3 Appendix). Moreover, to validate the consistency of our method in different outcome settings, we repeat our simulations under a binary outcome scenario (Text B in S3 Appendix). Also, to evaluate the robustness of our proposed GOAL-based method regarding the sparsity structure, we conduct a sensitivity analysis by incrementally increasing the number of true active signals in the data generation process (Text F in S3 Appendix). We additionally evaluate the robustness of our method using increased covariate-to-sample size ratios (*p*/*n*) from 0.1 to 0.9 to investigate its performance under varying dimensionality levels (Text G in S3 Appendix).

Further, we compare the performance of the proposed GOAL-integrated estimator with the full-covariate GPS-weighted semiparametric estimator and other regularization method-based methods, including AdaLASSO and LASSO. For AdaLASSO, we fit two linear regression models corresponding to two GPS models in our semiparametric estimators: one model regresses *A* on *X*, and the other model regresses *A* on *M* and *X*. The regression coefficients are then used to compute the corresponding penalty weights for two GPS models. The estimates of AdaLASSO and LASSO are presented in Text H in S3 Appendix. The tuning parameter *λ* is also chosen by minimizing deviance with 10-fold cross-validation. Additionally, to compare the performance of our method with other non-regularization approaches, such as modern machine learning methods, we follow the work of Yang et al. [26] to implement a double machine learning framework-based mediation analysis under high-dimensional confounding. Specifically, this method utilizes a partially linear mediation structure to isolate causal parameters and employs the machine learning algorithms (here we adopt the LASSO algorithm in our replication) to estimate nuisance parameters within the high-dimensional covariate space. Building on this framework, the mediation effects are subsequently estimated using traditional structural equation modeling, where the indirect effect is derived from the product of coefficients. Notably, under a linear model specification without exposure-mediator interaction or nonlinear effects, the direct and indirect effect estimates yielded from this approach are approximately comparable to the causal mediation effects defined in our potential outcome-based framework. Furthermore, we fit four benchmark models based on different subsets of covariates for comparison:

(i) Benchmark model (true): GPS models are fitted using *X*_*C*_ and *X*_*I*_, indicating “true” treatment models.

(ii) Benchmark model (outcome): GPS models are fitted using *X*_*C*_ and *X*_*P*_, which involve outcome information; the target model of our proposed method.

(iii) Benchmark model (true + outcome): GPS models are fitted using *X*_*C*_, *X*_*I*_, and *X*_*P*_.

(iv) Benchmark model (full): GPS models are fitted using *X*_*C*_, *X*_*I*_, *X*_*P*_, and 10 randomly selected *X*_*S*_, representing the natural GPS models without variable selection. Of note, we select 10 spurious covariates considering both the computational efficiency and the sufficiency of representativeness of the influence of spurious covariate variables.

### 4.5 Real data application

The established link between diabetes risk and cancer incidence [81–83] is increasingly attributed to metabolic dysregulation, particularly dyslipidemia [58–60]. Apolipoprotein B (ApoB), an essential structural and functional component of lipoprotein particles [84–86], has emerged as a key mechanistic link. Specifically, abnormal changes in ApoB levels have been widely suggested as potential downstream consequences of diabetic conditions [87–91]. In turn, abnormal ApoB-related disturbances are associated with carcinogenesis through mechanisms, including transduction, oxidative stress, and tumor proliferation [61,62,92–97]. These findings position ApoB as an underlying plausible mediator in the diabetes-cancer pathway. Furthermore, as large-scale databases with a vast quantity of variables become prevalent, traditional observational studies struggle to fully account for potential confounders based on established knowledge, making it difficult to deliver causal estimates. Therefore, we apply our method to investigate the mediating effect of ApoB levels on the association between the Finnish Diabetes Risk Score (FINDRISC)-represented potential diabetes risk and the overall incidence of 24 site-specific cancers with large-scale biomedical data obtained from the UK Biobank.

UK Biobank is a large population-based cohort study conducted in England from 2006 to 2010, comprising approximately 500,000 participants aged 40–69 years [98]. Based on the UK Biobank, the treatment variable *A* is defined as a continuous composite indicator of FINDRISC constructed by age, body mass index (BMI), waist circumference, activity, vegetable and fruit intake, history of hypertension medication, history of hyperglycemia, and family history of diabetes, ranging from 0 to 25 [99,100] (Table L in S1 Appendix). The outcome variable *Y* is a binary indicator of overall cancer occurrence defined by the first record of hospitalization for cancer, cancer registry, or self-reported data according to the International Classification of Diseases (ICD)-9, ICD-10, and self-reported codes (Table M in S1 Appendix). The mediator variable *M* is defined as a continuous variable of the ApoB levels. We include a total of 98 variables in the covariate space, covering a wide range of demographic characteristics, socioeconomic status, lifestyle and environmental factors, genetic risk indicators, physical and mental health-related factors and measures, and blood biochemical and metabolic biomarkers (Tables N-O in S1 Appendix). The data on FINDRISC and ApoB are collected or measured at baseline, and the information on cancer occurrence is obtained by linking data from the cancer and death registry. By integrating the GOAL method into the GPS models of the semiparametric estimation, we expect to select important variables from the large covariate space.

**Fig 7:**
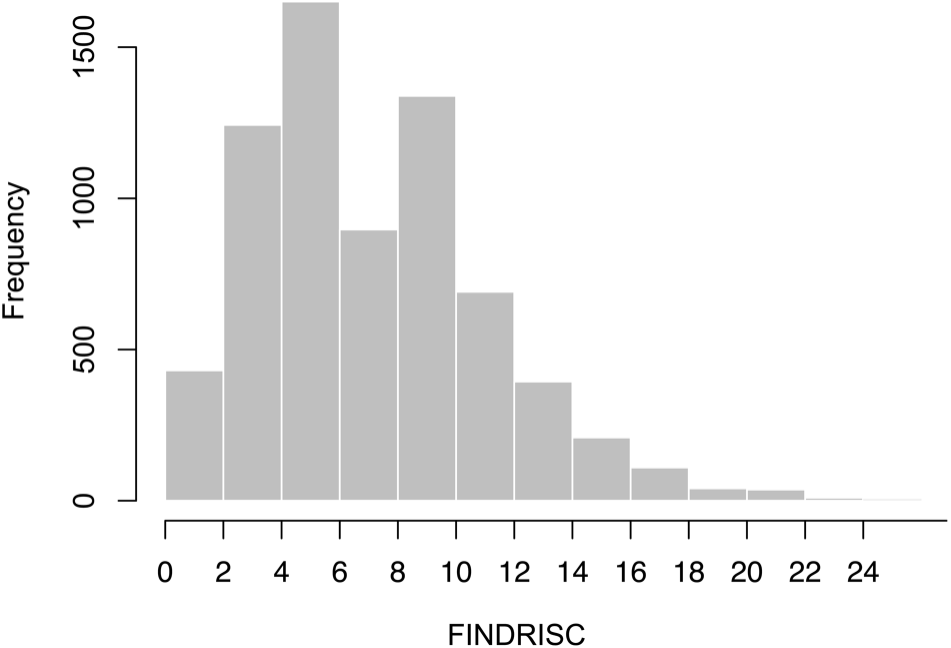
Density histogram of FINDRISC (treatment). **Notes:** The horizontal axis displays the treatment values, i.e., Finnish Diabetes Risk Score (FINDRISC), in increments of 2, and the vertical axis indicates the number of participants within each treatment interval. Abbreviations: FINDRISC, the Finnish Diabetes Risk Score.

We sequentially exclude the pregnant individuals, withdrawals, participants with missing information on FINDRISC, ApoB and cancer, and participants who reported cancer occurrence at baseline. These procedures restrict the analysis to individuals with a positive conditional treatment density and ensure the temporal sequence of the treatment and mediator preceding the outcome. We implement a complete case analysis where participants who had missingness on any of the 98 covariates are excluded. Ultimately, the analysis sample contains 7,054 participants, of which 1,057 developed cancer (*Y* = 1) and the remaining 5,997 did not (*Y* = 0), with a cancer incidence of 14.98%. The density distribution of FINDRISC is presented in Fig 7. The mean value of FINDRISC (*A*) of the 7,054 observations is 7.67, and the median value is 7. And the mean and median values of the level of ApoB (*M*) are 0.99 g/L and 0.98 g/L, respectively, with the density distribution plot in Fig J in S2 Appendix.

We examine the key assumptions underlying the application of this method to real-world data (Text I in S3 Appendix). We conduct an exploratory data analysis by using the test.TMint function in the R ‘mediation’ package [101] to preliminarily examine the interaction effect between *A* and *M*. The results all show no significant sign of an interaction effect between *A* and *M* (*P* = 0.416), implying the plausibility of omitting the interaction term in the subsequent analyses. In addition, we fit an outcome model with an *A*-*M* interaction term and the quadratic terms of *A* and *M* to pre-test the linearity of the outcome model*.* The results also indicate non-significant effects for the interaction term and quadratic terms (P(AM)=0.162, $P(A^{\text{2}})=\text{0}.\text{217}$ and $P(M^{\text{2}})=\text{0}.\text{899}$), providing supportive evidence for the appropriateness of the model linearity. Furthermore, we fit a generalized additive outcome model with *A* and the penalized spline term of the *M* using the R ‘mgcv’ package [102,103] to extensively capture the potential nonlinear effect of the mediator. The effective degrees of freedom (EDF) for the smooth term are close to 1 and not statistically significant (*P* = 0.324), suggesting no evidence of nonlinearity between the mediator and the outcome.

The mediation effects are evaluated using the GOAL-embedded GPS-weighted semiparametric estimation approach, where the weight for each observation is constructed by parametric GPSs of treatment density conditioned on the selected covariates or the covariates and the mediator (Equation 7). Also, analyses based on the GPS models with full covariates are performed for comparison. The convergence factor takes the value of 2 in all the variable selection procedures. Under the condition of positive treatment intensity, we estimate the direct and indirect effects under both treatment and non-treatment status in the treatment range of 3–13 in a step of 1 versus the reference value of 2, that is, ${\hat{\theta }}_{a,a^{\prime }}(a)$, ${\hat{\theta }}_{a,a^{\prime }}\left(a^{\prime }\right)$, ${\hat{\delta }}_{a,a^{\prime }}(a)$ and ${\hat{\delta }}_{a,a^{\prime }}\left(a^{\prime }\right)$ for each of *a* ∈ {3, 4,  ..., 13} and *a’* = 2. We employ the second-order Epanechnikov kernels and utilize the rule-of-thumb bandwidth *h* for kernel methods with *h* = *C*·sd(*A*)·*n*^-1/5^, where sd(*A*) is the standard deviation of *A* and *C* = 2.34. It is more pragmatic and widely adopted in the empirical studies with an unknown data-generating process to balance bias and variance, which was also consistently applied and described in Huber et al [5]. We also provide a set of diagnostic checks for a series of candidates covering simulation bandwidths and a set of ROT-based candidates that follows the conventional practice in kernel-based sensitivity analyses: $\{h_{wp},\;h_{wp\;us},\;\text{0}.\text{7}\text{5}h_{ROT},\;h_{ROT},\;\text{1}.\text{2}\text{5}h_{ROT},\text{1}.\text{5}h_{ROT},\;\text{2}h_{ROT}\}$ (Table P, Figs D-E, and Text C in S1–S3 Appendices). Further, to examine the sensitivity of kernel-based estimation to bandwidth specification, we repeat the estimation using a range of bandwidth constants *C* = 1.7, 2.0, 2.5 under the rule-of-thumb formula *h* = *C*·sd(*A*)·*n*^-1/5^, where *C* = 2.34 is compared as a reference (Table R in S1 Appendix and Figs H-I in S2 Appendix).

We derive the variance estimation by bootstrapping 500 times and measure the average standard deviation of the effects across all treatment values. We also conduct a sensitivity analysis with 100 bootstrap resamples for the GOAL-based estimation to visualize and examine the stability of variable selection under bootstrapping and regularization (Table Q in S1 Appendix and Figs F-G in S2 Appendix).

## Supporting information

S1 Appendix Tables A to R. All supplementary tables in the study, including mediation effect estimation across different bandwidth options and complex scenarios, bandwidth diagnostics results, robustness checks for bootstrap inference, model sparsity and dimensionality, as well as real-world data information.
(DOCX)

S2 Appendix Figs A to J. All supplementary figures in the study, including variable selection results for different sample sizes and model sparsity, effect estimation trajectories under different bandwidth options, and variable selection practices in real-world applications.
(DOCX)

S3 Appendix Texts A to I. All supplementary text in the study, including key assumptions of the method and their validation, simulation setups and algorithms for additional or complex scenarios, and theories and diagnostics related to bandwidth specification.
(DOCX)

## References

[1] Pearl J. Direct and indirect effects. Probabilistic and causal inference: the works of Judea Pearl. 2022. p. 373–92.

[2] Robins JM, Greenland S. Identifiability and exchangeability for direct and indirect effects. Epidemiology. 1992;3(2):143–55. doi: 10.1097/00001648-199203000-00013 1576220

[3] Hsu Y-C, Huber M, Lai T-C. Nonparametric estimation of natural direct and indirect effects based on inverse probability weighting. J Econometr Methods. 2019;8(1):20170016. doi: 10.1515/jem-2017-0016

[4] Huber M. Identifying causal mechanisms (primarily) based on inverse probability weighting. J Appl Econ. 2014;29(6):920–43. doi: 10.1002/jae.2341

[5] Huber M, Hsu Y, Lee Y, Lettry L. Direct and indirect effects of continuous treatments based on generalized propensity score weighting. J Appl Econometr. 2020;35(7):814–40. doi: 10.1002/jae.2765

[6] Rosenbaum PR, Rubin DB. The central role of the propensity score in observational studies for causal effects. Biometrika. 1983;70(1):41–55. doi: 10.1093/biomet/70.1.41

[7] Zhang Q, Xiao S, Jiao X, Shen Y. The triglyceride-glucose index is a predictor for cardiovascular and all-cause mortality in CVD patients with diabetes or pre-diabetes: evidence from NHANES 2001-2018. Cardiovasc Diabetol. 2023;22(1):279. doi: 10.1186/s12933-023-02030-z 37848879 PMC10583314

[8] Zhou J, Meng X, Deng L, Liu N. Non-linear associations between metabolic syndrome and four typical heavy metals: Data from NHANES 2011-2018. Chemosphere. 2022;291(Pt 2):132953. doi: 10.1016/j.chemosphere.2021.132953 34800500

[9] Huang L, Huang W, Linton O, Zhang Z. Nonparametric estimation of mediation effects with a general treatment. Econ Rev. 2024;43(2–4):215–37. doi: 10.1080/07474938.2024.2314092

[10] Singh R, Xu L, Gretton A. Sequential Kernel Embedding for Mediated and Time-Varying Dose Response Curves. arXiv preprint arXiv:211103950. 2021.

[11] MacCallum RC, Zhang S, Preacher KJ, Rucker DD. On the practice of dichotomization of quantitative variables. Psychol Methods. 2002;7(1):19–40. doi: 10.1037/1082-989x.7.1.19 11928888

[12] Fong C, Hazlett C, Imai K. Covariate balancing propensity score for a continuous treatment: Application to the efficacy of political advertisements. Ann Appl Stat. 2018;12(1):156–77.

[13] Hirano K, Imbens GW. The propensity score with continuous treatments. Applied Bayesian modeling and causal inference from incomplete‐data perspectives. 2004. p. 73–84.

[14] Imai K, Van Dyk DA. Causal inference with general treatment regimes: Generalizing the propensity score. J Am Stat Assoc. 2004;99(467):854–66.

[15] Imbens G. The role of the propensity score in estimating dose-response functions. Biometrika. 2000;87(3):706–10. doi: 10.1093/biomet/87.3.706

[16] Wilson A, Reich BJ. Confounder selection via penalized credible regions. Biometrics. 2014;70(4):852–61. doi: 10.1111/biom.12203 25123966

[17] Ertefaie A, Asgharian M, Stephens DA. Variable Selection in Causal Inference using a Simultaneous Penalization Method. J Causal Inference. 2017;6(1). doi: 10.1515/jci-2017-0010

[18] Brookhart MA, Schneeweiss S, Rothman KJ, Glynn RJ, Avorn J, Stürmer T. Variable selection for propensity score models. Am J Epidemiol. 2006;163(12):1149–56. doi: 10.1093/aje/kwj149 16624967 PMC1513192

[19] Tibshirani R. Regression Shrinkage and Selection Via the Lasso. J R Stat Soc Ser B Stat Methodol. 1996;58(1):267–88. doi: 10.1111/j.2517-6161.1996.tb02080.x

[20] Zou H. The Adaptive Lasso and Its Oracle Properties. J Am Stat Assoc. 2006;101(476):1418–29. doi: 10.1198/016214506000000735

[21] Shortreed SM, Ertefaie A. Outcome-adaptive lasso: Variable selection for causal inference. Biometrics. 2017;73(4):1111–22. doi: 10.1111/biom.12679 28273693 PMC5591052

[22] Gao Q, Zhang Y, Liang J, Sun H, Wang T. High-dimensional generalized propensity score with application to omics data. Brief Bioinform. 2021;22(6):bbab331. doi: 10.1093/bib/bbab331 34410351

[23] Ye Z, Zhu Y, Coffman DL. Variable selection for causal mediation analysis using LASSO-based methods. Stat Methods Med Res. 2021;30(6):1413–27. doi: 10.1177/0962280221997505 33755518 PMC8189011

[24] Farbmacher H, Huber M, Lafférs L, Langen H, Spindler M. Causal mediation analysis with double machine learning. Econ J. 2022;25(2):277–300. doi: 10.1093/ectj/utac003

[25] Devick KL, Bobb JF, Mazumdar M, Claus Henn B, Bellinger DC, Christiani DC, et al. Bayesian kernel machine regression-causal mediation analysis. Stat Med. 2022;41(5):860–76. doi: 10.1002/sim.9255 34993981 PMC9150437

[26] Yang J, Shao Y, Liu J, Wang L. Double machine learning for partially linear mediation models with high-dimensional confounders. Neurocomputing. 2025;614:128766. doi: 10.1016/j.neucom.2024.128766

[27] Díaz I, Hejazi NS. Causal Mediation Analysis for Stochastic Interventions. J R Stat Soc Ser B Stat Methodol. 2020;82(3):661–83. doi: 10.1111/rssb.12362

[28] Zheng W, van der Laan MJ. Targeted maximum likelihood estimation of natural direct effects. Int J Biostat. 2012;8(1). doi: 10.2202/1557-4679.1361 22499725 PMC6055937

[29] Liu R, Williams NT, Rudolph KE, Díaz I. General targeted machine learning for modern causal mediation analysis2024 August 01, 2024:[arXiv:2408.14620 p.]. Available from: https://ui.adsabs.harvard.edu/abs/2024arXiv240814620L

[30] Kennedy EH. Nonparametric Causal Effects Based on Incremental Propensity Score Interventions. J Am Stat Assoc. 2019;114(526):645–56. doi: 10.1080/01621459.2017.1422737

[31] Ullah A, Pagan A. Nonparametric econometrics. Cambridge: Cambridge University Press; 1999.

[32] Rubin DB. Estimating causal effects from large data sets using propensity scores. Ann Intern Med. 1997;127(8 Pt 2):757–63. doi: 10.7326/0003-4819-127-8_part_2-199710151-00064 9382394

[33] Zhou Y, Kivimäki M, Yan LL, Carrillo-Larco RM, Zhang Y, Cheng Y, et al. Associations between socioeconomic inequalities and progression to psychological and cognitive multimorbidities after onset of a physical condition: a multicohort study. EClinicalMedicine. 2024;74:102739. doi: 10.1016/j.eclinm.2024.102739 39157288 PMC11327438

[34] Wang D, Dai X, Mishra SR, Lim CCW, Carrillo-Larco RM, Gakidou E, et al. Association between socioeconomic status and health behaviour change before and after non-communicable disease diagnoses: a multicohort study. Lancet Public Health. 2022;7(8):e670–82. doi: 10.1016/S2468-2667(22)00157-8 35907418

[35] Li Y, Schoufour J, Wang DD, Dhana K, Pan A, Liu X, et al. Healthy lifestyle and life expectancy free of cancer, cardiovascular disease, and type 2 diabetes: prospective cohort study. BMJ. 2020;368:l6669. doi: 10.1136/bmj.l6669 31915124 PMC7190036

[36] Patterson R, McNamara E, Tainio M, de Sá TH, Smith AD, Sharp SJ, et al. Sedentary behaviour and risk of all-cause, cardiovascular and cancer mortality, and incident type 2 diabetes: a systematic review and dose response meta-analysis. Eur J Epidemiol. 2018;33(9):811–29. doi: 10.1007/s10654-018-0380-1 29589226 PMC6133005

[37] van Dieren S, Uiterwaal CSPM, van der Schouw YT, van der A DL, Boer JMA, Spijkerman A, et al. Coffee and tea consumption and risk of type 2 diabetes. Diabetologia. 2009;52(12):2561–9. doi: 10.1007/s00125-009-1516-3 19727658

[38] Sharma S, Tapper WJ, Collins A, Hamady ZZR. Predicting Pancreatic Cancer in the UK Biobank Cohort Using Polygenic Risk Scores and Diabetes Mellitus. Gastroenterology. 2022;162(6):1665-1674.e2. doi: 10.1053/j.gastro.2022.01.016 35065983

[39] Mars N, Koskela JT, Ripatti P, Kiiskinen TTJ, Havulinna AS, Lindbohm JV, et al. Polygenic and clinical risk scores and their impact on age at onset and prediction of cardiometabolic diseases and common cancers. Nat Med. 2020;26(4):549–57. doi: 10.1038/s41591-020-0800-0 32273609

[40] Avogaro A. Diabetes and obesity: the role of stress in the development of cancer. Endocrine. 2024;86(1):48–57. doi: 10.1007/s12020-024-03886-1 38831236 PMC11445296

[41] Hughes K, Bellis MA, Hardcastle KA, Sethi D, Butchart A, Mikton C, et al. The effect of multiple adverse childhood experiences on health: a systematic review and meta-analysis. Lancet Public Health. 2017;2(8):e356–66. doi: 10.1016/S2468-2667(17)30118-4 29253477

[42] Asslih S, Damri O, Agam G. Neuroinflammation as a Common Denominator of Complex Diseases (Cancer, Diabetes Type 2, and Neuropsychiatric Disorders). Int J Mol Sci. 2021;22(11):6138. doi: 10.3390/ijms22116138 34200240 PMC8201050

[43] Thornton SN. Angiotensin, the hypovolaemia hormone, aggravates hypertension, obesity, diabetes and cancer. J Intern Med. 2009;265(5):616–7. doi: 10.1111/j.1365-2796.2008.02037.x 19019186

[44] Yang X, So WY, Ma RCW, Ko GTC, Kong APS, Zhao H, et al. Low LDL cholesterol, albuminuria, and statins for the risk of cancer in type 2 diabetes: the Hong Kong diabetes registry. Diabetes Care. 2009;32(10):1826–32. doi: 10.2337/dc09-0725 19592629 PMC2752941

[45] Hamdy NM, Mosaad YO, Elshimy R, Hady AA, Lin Q, Jastaniah Z, et al. Unraveling the genetic links between obesity or insulin resistance and breast cancer through the impact of CD295 and ITLN1 SNPs with DNA damage in a case-controlled study with bioinformatics analysis. Front Med (Lausanne). 2025;12:1703759. doi: 10.3389/fmed.2025.1703759 41221514 PMC12598017

[46] Samuels E, Parks J, Chu J, McDonald T, Spinelli J, Murphy RA, et al. Metabolites Associated with Polygenic Risk of Breast Cancer. Metabolites. 2024;14(6):295. doi: 10.3390/metabo14060295 38921430 PMC11205321

[47] Kuchenbaecker KB, Hopper JL, Barnes DR, Phillips K-A, Mooij TM, Roos-Blom M-J, et al. Risks of Breast, Ovarian, and Contralateral Breast Cancer for BRCA1 and BRCA2 Mutation Carriers. JAMA. 2017;317(23):2402–16. doi: 10.1001/jama.2017.7112 28632866

[48] Nestsiarovich A, Kerner B, Mazurie AJ, Cannon DC, Hurwitz NG, Zhu Y, et al. Diabetes mellitus risk for 102 drugs and drug combinations used in patients with bipolar disorder. Psychoneuroendocrinology. 2020;112:104511. doi: 10.1016/j.psyneuen.2019.104511 31744781

[49] Elhussein L, Williams RD, Man WY, Burn E, Delmestri A, Strauss VY, et al. Longitudinal trajectories of polypharmacy in older people, and their association with the risk of mortality: a joint latent class model analysis of real-world data from the UK and the Netherlands. Age Ageing. 2025;54(8):afaf233. doi: 10.1093/ageing/afaf233 40833208 PMC12365978

[50] Son JW, Lee SS, Kim SR, Yoo SJ, Cha BY, Son HY, et al. Low muscle mass and risk of type 2 diabetes in middle-aged and older adults: findings from the KoGES. Diabetologia. 2017;60(5):865–72. doi: 10.1007/s00125-016-4196-9 28102434

[51] Xu Z, Luo X, Diao W, Tang X, Zhang Y, Wang J, et al. Contributing effects of sarcopenia on cancer occurrence: novel evidence based on NHANES 1999-2020 and two-sample mendelian randomization study. Oncologist. 2025;30(11):oyaf369. doi: 10.1093/oncolo/oyaf369 41206069 PMC12628308

[52] Shen W, Cai L, Wang B, Li J, Sun Y, Chen Y, et al. Associations of a proinflammatory diet, habitual salt intake, and the onset of type 2 diabetes: A prospective cohort study from the UK Biobank. Diabetes Obes Metab. 2024;26(6):2119–27. doi: 10.1111/dom.15517 38409502

[53] Tang S, Xu J, Wan P, Jin S, Zhang Y, Xun L, et al. Recent advances in the role of high-salt diet in anti- and pro-cancer progression. Front Immunol. 2025;16:1542157. doi: 10.3389/fimmu.2025.1542157 39944693 PMC11814453

[54] Nichols GA, Amitay EL, Chatterjee S, Steubl D. The Bidirectional Association of Chronic Kidney Disease, Type 2 Diabetes, Atherosclerotic Cardiovascular Disease, and Heart Failure: The Cardio-Renal-Metabolic Syndrome. Metab Syndr Relat Disord. 2023;21(5):261–6. doi: 10.1089/met.2023.0006 37130317

[55] Mok Y, Surapaneni A, Sang Y, Coresh J, Grams ME, Matsushita K, et al. Chronic kidney disease and incident cancer risk: an individual participant data meta-analysis. Br J Cancer. 2025;133(10):1535–43. doi: 10.1038/s41416-025-03140-z 40914744 PMC12603274

[56] Peng Y, Wang P, Gong J, Liu F, Qiao Y, Si C, et al. Association between the Finnish Diabetes Risk Score and cancer in middle-aged and older adults: Involvement of inflammation. Metabolism. 2023;144:155586. doi: 10.1016/j.metabol.2023.155586 37164309

[57] Chang W-C, Hsieh T-C, Hsu W-L, Chang F-L, Tsai H-R, He M-S. Diabetes and further risk of cancer: a nationwide population-based study. BMC Med. 2024;22(1):214. doi: 10.1186/s12916-024-03430-y 38807177 PMC11134680

[58] Kim DS, Scherer PE. Obesity, diabetes, and increased cancer progression. Diabetes Metab J. 2021;45(6):799–812. 34847640 10.4093/dmj.2021.0077 PMC8640143

[59] Lega IC, Lipscombe LL. Review: Diabetes, Obesity, and Cancer-Pathophysiology and Clinical Implications. Endocr Rev. 2020;41(1):bnz014. doi: 10.1210/endrev/bnz014 31722374

[60] Wang X, Ding S. The biological and pharmacological connections between diabetes and various types of cancer. Pathol Res Pract. 2021;227:153641. doi: 10.1016/j.prp.2021.153641 34619575

[61] Zhang Y, Zheng L. Apolipoprotein: prospective biomarkers in digestive tract cancer. Transl Cancer Res. 2020;9(5):3712–20. doi: 10.21037/tcr-19-2106 35117733 PMC8799137

[62] Mazidi M, Katsiki N, Mikhailidis DP, Radenkovic D, Pella D, Banach M. Apolipoprotein B/Apolipoprotein A-I Ratio Is a Better Predictor of Cancer Mortality Compared with C-Reactive Protein: Results from Two Multi-Ethnic US Populations. J Clin Med. 2020;9(1). 10.3390/jcm9010170 PMC7019626 31936330

[63] Yang C-L, Chao Y-J, Wang H-C, Hou Y-C, Chen CG, Chang C-C, et al. Local ablation of gastric cancer by reconstituted apolipoprotein B lipoparticles carrying epigenetic drugs. Nanomedicine. 2021;37:102450. doi: 10.1016/j.nano.2021.102450 34332115

[64] Parlesak A, Eckoldt J, Winkler K, Bode CJ, Schäfer C. Intercorrelations of lipoprotein subfractions and their covariation with lifestyle factors in healthy men. J Clin Biochem Nutr. 2014;54(3):174–80. doi: 10.3164/jcbn.13-78 24895480 PMC4042151

[65] Frondelius K, Borg M, Ericson U, Borné Y, Melander O, Sonestedt E. Lifestyle and dietary determinants of serum apolipoprotein A1 and apolipoprotein B concentrations: cross-sectional analyses within a Swedish cohort of 24,984 individuals. Nutrients. 2017;9(3). 10.3390/nu9030211 PMC5372874 28264492

[66] Efron B. Estimation and accuracy after model selection. J Am Stat Assoc. 2014;109(507):991–1007. 25346558 10.1080/01621459.2013.823775 PMC4207812

[67] Leeb H, Pötscher BM. Model selection and inference: facts and fiction. Econom Theory. 2005;21(1):21–59. doi: 10.1017/s0266466605050036

[68] Alhamzawi R, Ali HTM. The Bayesian adaptive lasso regression. Math Biosci. 2018;303:75–82. doi: 10.1016/j.mbs.2018.06.004 29920251

[69] Bühlmann P, Van De Geer S. Statistics for high-dimensional data: methods, theory and applications. Springer Science & Business Media; 2011.

[70] Loh PL, Bühlmann P. High-dimensional learning of linear causal networks via inverse covariance estimation. J Mach Learn Res. 2014;15(140):3065–105.

[71] Ramsey J, Glymour M, Sanchez-Romero R, Glymour C. A million variables and more: the Fast Greedy Equivalence Search algorithm for learning high-dimensional graphical causal models, with an application to functional magnetic resonance images. Int J Data Sci Anal. 2017;3(2):121–9. doi: 10.1007/s41060-016-0032-z 28393106 PMC5380925

[72] Maathuis MH, Kalisch M, Bühlmann P. Estimating high-dimensional intervention effects from observational data. 2009.

[73] Rubin DB. Estimating causal effects of treatments in randomized and nonrandomized studies. J Educ Psychol. 1974;66(5):688–701. doi: 10.1037/h0037350

[74] Rubin DB. Direct and Indirect Causal Effects via Potential Outcomes*. Scandinavian J Statistics. 2004;31(2):161–70. doi: 10.1111/j.1467-9469.2004.02-123.x

[75] Have TRT, Joffe MM, Lynch KG, Brown GK, Maisto SA, Beck AT. Causal mediation analyses with rank preserving models. Biometrics. 2007;63(3):926–34. doi: 10.1111/j.1541-0420.2007.00766.x 17825022

[76] Albert JM. Mediation analysis via potential outcomes models. Stat Med. 2008;27(8):1282–304. doi: 10.1002/sim.3016 17691077

[77] Effron B, Tibshirani RJ. An introduction to the bootstrap. New York: Chapman & Hall; 1993.

[78] Chernozhukov V, Chetverikov D, Demirer M, Duflo E, Hansen C, Newey W, et al. Double/debiased machine learning for treatment and causal parameters. 2024.

[79] Silverman BW. Density Estimation for Statistics and Data Analysis. Density Estimation For Statistics And Data Analysis; 1986.

[80] Wand MP, Jones MC. Kernel Smoothing. 1994.

[81] Satija A, Spiegelman D, Giovannucci E, Hu FB. Type 2 diabetes and risk of cancer. BMJ. 2015;350:g7707. doi: 10.1136/bmj.g7707 25555822

[82] Giovannucci E, Harlan DM, Archer MC, Bergenstal RM, Gapstur SM, Habel LA, et al. Diabetes and cancer: a consensus report. Diabetes Care. 2010;33(7):1674–85. doi: 10.2337/dc10-0666 20587728 PMC2890380

[83] Shi Y, Hu FB. The global implications of diabetes and cancer. Lancet. 2014;383(9933):1947–8. doi: 10.1016/S0140-6736(14)60886-2 24910221

[84] Wolska A, Lloyd-Jones DM, Remaley AT. Measure apolipoprotein B if we believe what we say about precision medicine. Circulation. 2025;151(5):257–9. 10.1161/CIRCULATIONAHA.123.067559 PMC11801237 39899636

[85] Borén J, Packard CJ, Binder CJ. Apolipoprotein B-containing lipoproteins in atherogenesis. Nat Rev Cardiol. 2025;22(6):399–413. doi: 10.1038/s41569-024-01111-0 39743565

[86] Borén J, Chapman MJ, Krauss RM, Packard CJ, Bentzon JF, Binder CJ, et al. Low-density lipoproteins cause atherosclerotic cardiovascular disease: pathophysiological, genetic, and therapeutic insights: a consensus statement from the European Atherosclerosis Society Consensus Panel. Eur Heart J. 2020;41(24):2313–30. doi: 10.1093/eurheartj/ehz962 32052833 PMC7308544

[87] Duvillard L, Pont F, Florentin E, Galland-Jos C, Gambert P, Vergès B. Metabolic abnormalities of apolipoprotein B-containing lipoproteins in non-insulin-dependent diabetes: a stable isotope kinetic study. Eur J Clin Invest. 2000;30(8):685–94. doi: 10.1046/j.1365-2362.2000.00755.x 10964160

[88] Zheng S, Han T, Xu H, Zhou H, Ren X, Wu P, et al. Associations of apolipoprotein B/apolipoprotein A-I ratio with pre-diabetes and diabetes risks: a cross-sectional study in Chinese adults. BMJ Open. 2017;7(1):e014038. doi: 10.1136/bmjopen-2016-014038 28110289 PMC5253599

[89] Gao L, Zhang Y, Wang X, Dong H. Association of apolipoproteins A1 and B with type 2 diabetes and fasting blood glucose: a cross-sectional study. BMC Endocr Disord. 2021;21(1):59. doi: 10.1186/s12902-021-00726-5 33794863 PMC8017773

[90] Ginsberg HN. Insulin resistance and cardiovascular disease. J Clin Invest. 2000;106(4):453–8. doi: 10.1172/JCI10762 10953019 PMC380256

[91] Erkelens DW. Diabetic dyslipidaemia. Eur Heart J. 1998;19 Suppl H:H27–40. 9717062

[92] Liu X, Yu H, Yan G, Xu B, Sun M, Feng M. Causal relationships between coffee intake, apolipoprotein B and gastric, colorectal, and esophageal cancers: univariable and multivariable Mendelian randomization. Eur J Nutr. 2024;63(2):469–83. doi: 10.1007/s00394-023-03281-y 38040849

[93] Li C, Yang X, Zhong Y, Wang W, Jin X, Bian L, et al. Apolipoprotein B/Apolipoprotein A1 ratio is an independent prognostic factor in pancreatic cancer. Transl Oncol. 2025;51:102208. doi: 10.1016/j.tranon.2024.102208 39591897 PMC11629317

[94] Marrone MT, Prizment AE, Couper D, Butler KR, Astor BC, Joshu CE, et al. Total-, LDL-, and HDL-cholesterol, apolipoproteins, and triglycerides with risk of total and fatal prostate cancer in Black and White men in the ARIC study. Prostate. 2023;83(11):1046–59. doi: 10.1002/pros.24546 37154584 PMC12478469

[95] Baenke F, Peck B, Miess H, Schulze A. Hooked on fat: the role of lipid synthesis in cancer metabolism and tumour development. Dis Model Mech. 2013;6(6):1353–63. doi: 10.1242/dmm.011338 24203995 PMC3820259

[96] He Y, Chen J, Ma Y, Chen H. Apolipoproteins: New players in cancers. Front Pharmacol. 2022;13:1051280. 36506554 10.3389/fphar.2022.1051280 PMC9732396

[97] Tate EW, Soday L, de la Lastra AL, Wang M, Lin H. Protein lipidation in cancer: mechanisms, dysregulation and emerging drug targets. Nat Rev Cancer. 2024;24(4):240–60. doi: 10.1038/s41568-024-00666-x 38424304

[98] Sudlow C, Gallacher J, Allen N, Beral V, Burton P, Danesh J, et al. UK biobank: an open access resource for identifying the causes of a wide range of complex diseases of middle and old age. PLoS Med. 2015;12(3):e1001779. doi: 10.1371/journal.pmed.1001779 25826379 PMC4380465

[99] Lindström J, Tuomilehto J. The diabetes risk score: a practical tool to predict type 2 diabetes risk. Diabetes Care. 2003;26(3):725–31. doi: 10.2337/diacare.26.3.725 12610029

[100] Pesaro AE, Bittencourt MS, Franken M, Carvalho JAM, Bernardes D, Tuomilehto J, et al. The Finnish Diabetes Risk Score (FINDRISC), incident diabetes and low-grade inflammation. Diabetes Res Clin Pract. 2021;171:108558. doi: 10.1016/j.diabres.2020.108558 33242513

[101] Tingley D, Yamamoto T, Hirose K, Keele L, Imai K. mediation:RPackage for Causal Mediation Analysis. J Stat Soft. 2014;59(5). doi: 10.18637/jss.v059.i05

[102] Wood SN. Generalized additive models: an introduction with R. Chapman and Hall/CRC; 2017.

[103] Hastie T, Tibshirani R. Generalized Additive Models. Statist Sci. 1986;1(3). doi: 10.1214/ss/1177013604 8548102

