# Supplementary material for: Variable selection-combined causal mediation analysis for continuous treatments with application to large-dimensional biomedical data
Source: PLoS Comput Biol. 2026 Jun 24;22(6):e1014436. doi: 10.1371/journal.pcbi.1014436 (PMC13327524; doi:10.1371/journal.pcbi.1014436)
Supplement: S1 Appendix — All supplementary tables in the study, including mediation effect estimation across different bandwidth options and complex scenarios, bandwidth diagnostics results, robustness checks for bootstrap inference, model sparsity and dimensionality, as well as real-world data information. (DOCX) [file pcbi.1014436.s001.docx]

# S1 Appendix

**Variable selection-combined causal mediation analysis for continuous treatments with application to large-dimensional biomedical data**

Yajing Zhou^1^, Kecheng Wei^1^, Yahang Liu^1^, Zhaoyang Li^1^, Chen Huang^1^, Guoyou Qin^2*^, Yongfu Yu^1,3*^

^1^ Department of Biostatistics, NHC Key Laboratory for Health Technology Assessment, Key Laboratory of Public Health Safety of Ministry of Education, School of Public Health, Fudan University, Shanghai 200032, China

^2^ Shanghai Institute of Infectious Disease and Biosecurity, Fudan University, Shanghai, China

^3^ Shanghai Key Laboratory of Gene Editing and Cell Therapy for Rare Diseases, Fudan University, Shanghai 200031, China

* [yu@fudan.edu.cn](mailto:yu@fudan.edu.cn); [gyqin@fudan.edu.cn](mailto:gyqin@fudan.edu.cn)

**Contents**

**Table A.** Mediation effects estimated by weighting with a parametric generalized propensity score based on different covariate sets under Scenario 1 (SoSt) using the semiparametric kernel bandwidth.

**Table B.** Mediation effects estimated by weighting with a parametric generalized propensity score based on different covariate sets under Scenario 2 (SoWt) using the semiparametric kernel bandwidth.

**Table C.** Mediation effects estimated by weighting with a parametric generalized propensity score based on different covariate sets under Scenario 3 (WoSt) using the semiparametric kernel bandwidth.

**Table D.** Bandwidth diagnostic results in simulation scenarios.

**Table E**. Stability check of bootstrap-based variance estimates.

**Table F**. Robustness check of interactive and nonlinear outcome models using the GOAL-based method.

**Table G**. Detailed coverage probability performance across the continuous exposure range.

**Table H**. Robustness test regarding the method sparsity of the proposed GOAL-based method.

**Table I.** Investigation of the estimation performance of the GOAL-based method under varying covariate-to-sample size ratios.

**Table J.** Simulation performance of the GOAL-based estimation under normally distributed errors.

**Table K.** Simulation performance of the GOAL-based estimation under binary outcome settings.

**Table L.** Scoring of the Finnish Diabetes Risk Score (FINDRISC).

**Table M.** Data source for the definition of overall cancer in the UK Biobank.

**Table N.** Descriptive information of variables used in the real data application.

**Table O.** Details of variable coding in the real data application.

**Table P.** Bandwidth diagnostic results in the real-data application.

**Table Q**. Bootstrap-based average standard deviations for the GOAL-based estimation.

**Table R**. Sensitivity analysis using varying bandwidths in the real data application.

## Table A. Mediation effects estimated by weighting with a parametric generalized propensity score based on different covariate sets under Scenario 1 (SoSt) using the semiparametric kernel bandwidth.

| **Sample Size** | **Methods** | **Correlation (rho)** | ${\hat{\boldsymbol{\theta}}}_{\boldsymbol{a,a’}}\left( \boldsymbol{a} \right)$ | | | | ${\hat{\boldsymbol{\theta}}}_{\boldsymbol{a,a’}}\left( \boldsymbol{a’} \right)$ | | | | ${\hat{\boldsymbol{\delta}}}_{\boldsymbol{a,a’}}\left( \boldsymbol{a} \right)$ | | | | ${\hat{\boldsymbol{\delta}}}_{\boldsymbol{a,a’}}\left( \boldsymbol{a’} \right)$ | | | |
| --- | --- | --- | --- | --- | --- | --- | --- | --- | --- | --- | --- | --- | --- | --- | --- | --- | --- | --- |
|  |  |  | **Bias (%)** | **SD** | **RMSE** | **CP (%)** | **Bias (%)** | **SD** | **RMSE** | **CP (%)** | **Bias (%)** | **SD** | **RMSE** | **CP (%)** | **Bias (%)** | **SD** | **RMSE** | **CP (%)** |
| ***n*=2000** | GOAL | 0 | 9.08 | 0.046 | 0.070 | 80.0 | 8.97 | 0.046 | 0.070 | 79.3 | 10.51 | 0.009 | 0.009 | 91.7 | 11.75 | 0.008 | 0.009 | 92.4 |
|  |  | 0.3 | 14.77 | 0.053 | 0.098 | 66.7 | 14.94 | 0.053 | 0.099 | 66.9 | 5.41 | 0.009 | 0.009 | 94.5 | 5.10 | 0.009 | 0.009 | 67.6 |
|  | AdaLASSO | 0 | 15.68 | 0.052 | 0.098 | 64.0 | 15.48 | 0.051 | 0.097 | 64.5 | 4.59 | 0.010 | 0.010 | 95.7 | 4.84 | 0.010 | 0.009 | 97.1 |
|  |  | 0.3 | 24.72 | 0.058 | 0.143 | 41.8 | 25.11 | 0.057 | 0.144 | 39.1 | 12.88 | 0.011 | 0.012 | 92.9 | 17.24 | 0.010 | 0.013 | 34.6 |
|  | LASSO | 0 | 15.63 | 0.052 | 0.099 | 89.8 | 15.43 | 0.051 | 0.098 | 89.8 | 4.59 | 0.010 | 0.010 | 96.4 | 5.00 | 0.009 | 0.009 | 97.8 |
|  |  | 0.3 | 25.31 | 0.056 | 0.145 | 42.6 | 25.64 | 0.056 | 0.147 | 41.2 | 12.79 | 0.010 | 0.011 | 91.8 | 16.45 | 0.009 | 0.011 | 37.6 |
|  | Benchmark (True) | 0 | 14.64 | 0.050 | 0.098 | 87.1 | 14.47 | 0.050 | 0.097 | 85.3 | 5.07 | 0.009 | 0.009 | 63.9 | 6.17 | 0.009 | 0.008 | 70.6 |
|  |  | 0.3 | 23.28 | 0.056 | 0.145 | 87.9 | 23.41 | 0.056 | 0.147 | 93.1 | 7.01 | 0.010 | 0.011 | 1.7 | 8.48 | 0.009 | 0.011 | 0.3 |
|  | Benchmark (Outcome) | 0 | 7.74 | 0.046 | 0.066 | 81.6 | 7.63 | 0.046 | 0.066 | 81.7 | 10.70 | 0.008 | 0.009 | 92.2 | 11.92 | 0.008 | 0.009 | 91.8 |
|  |  | 0.3 | 12.04 | 0.052 | 0.088 | 73.5 | 11.98 | 0.052 | 0.087 | 73.1 | 9.11 | 0.009 | 0.010 | 93.4 | 9.81 | 0.009 | 0.009 | 95.7 |
|  | Benchmark (True+Outcome) | 0 | 14.63 | 0.050 | 0.098 | 87.5 | 14.47 | 0.050 | 0.097 | 85.1 | 4.97 | 0.009 | 0.009 | 64.2 | 5.98 | 0.009 | 0.008 | 69.3 |
|  |  | 0.3 | 23.18 | 0.056 | 0.145 | 92.6 | 23.31 | 0.056 | 0.146 | 95.5 | 6.99 | 0.010 | 0.011 | 7.4 | 8.42 | 0.009 | 0.011 | 4.1 |
|  | Benchmark (Full) | 0 | 14.64 | 0.051 | 0.098 | 63.2 | 14.47 | 0.051 | 0.097 | 63.3 | 4.88 | 0.009 | 0.009 | 97.0 | 5.90 | 0.009 | 0.008 | 98.1 |
|  |  | 0.3 | 23.13 | 0.057 | 0.145 | 43.1 | 23.25 | 0.056 | 0.146 | 41.9 | 7.17 | 0.010 | 0.011 | 91.9 | 8.56 | 0.009 | 0.011 | 88.9 |
| ***n*=5000** | GOAL | 0 | 4.11 | 0.037 | 0.044 | 90.8 | 3.97 | 0.037 | 0.044 | 91.1 | 5.76 | 0.006 | 0.007 | 89.9 | 7.32 | 0.006 | 0.007 | 89.4 |
|  |  | 0.3 | 10.92 | 0.042 | 0.072 | 72.1 | 11.08 | 0.042 | 0.073 | 71.6 | 3.55 | 0.007 | 0.007 | 91.7 | 4.55 | 0.006 | 0.007 | 91.1 |
|  | AdaLASSO | 0 | 8.72 | 0.041 | 0.067 | 74.7 | 9.72 | 0.040 | 0.066 | 75.4 | 9.57 | 0.007 | 0.007 | 96.5 | 3.21 | 0.007 | 0.006 | 96.8 |
|  |  | 0.3 | 8.68 | 0.045 | 0.115 | 43.3 | 9.63 | 0.045 | 0.118 | 40.4 | 9.52 | 0.007 | 0.009 | 85.0 | 3.04 | 0.007 | 0.012 | 76.2 |
|  | LASSO | 0 | 18.51 | 0.040 | 0.067 | 75.2 | 18.65 | 0.040 | 0.066 | 76.3 | 19.11 | 0.007 | 0.006 | 95.1 | 11.80 | 0.006 | 0.006 | 95.0 |
|  |  | 0.3 | 18.87 | 0.045 | 0.113 | 39.5 | 19.17 | 0.045 | 0.116 | 36.2 | 19.66 | 0.007 | 0.009 | 86.9 | 10.11 | 0.007 | 0.011 | 76.3 |
|  | Benchmark (True) | 0 | 8.72 | 0.040 | 0.067 | 93.0 | 8.61 | 0.040 | 0.067 | 93.7 | 3.36 | 0.006 | 0.006 | 62.9 | 3.42 | 0.006 | 0.006 | 64.8 |
|  |  | 0.3 | 16.86 | 0.044 | 0.115 | 70.8 | 17.25 | 0.044 | 0.119 | 83.5 | 6.24 | 0.007 | 0.008 | 0.3 | 10.58 | 0.006 | 0.011 | 0.0 |
|  | Benchmark (Outcome) | 0 | 3.22 | 0.036 | 0.043 | 90.3 | 3.08 | 0.036 | 0.043 | 91.0 | 6.29 | 0.006 | 0.007 | 90.1 | 7.79 | 0.006 | 0.007 | 87.5 |
|  |  | 0.3 | 8.16 | 0.041 | 0.067 | 77.9 | 8.23 | 0.041 | 0.068 | 78.0 | 6.20 | 0.006 | 0.007 | 91.7 | 5.56 | 0.006 | 0.007 | 88.6 |
|  | Benchmark (True+Outcome) | 0 | 8.79 | 0.040 | 0.067 | 92.9 | 8.68 | 0.040 | 0.067 | 93.9 | 3.31 | 0.006 | 0.006 | 62.0 | 3.32 | 0.006 | 0.006 | 63.7 |
|  |  | 0.3 | 17.50 | 0.044 | 0.115 | 83.6 | 17.92 | 0.044 | 0.119 | 91.9 | 5.80 | 0.007 | 0.008 | 2.5 | 10.47 | 0.006 | 0.011 | 0.0 |
|  | Benchmark (Full) | 0 | 8.79 | 0.040 | 0.067 | 75.3 | 8.66 | 0.040 | 0.067 | 75.6 | 3.28 | 0.006 | 0.006 | 95.4 | 3.25 | 0.006 | 0.006 | 94.8 |
|  |  | 0.3 | 17.53 | 0.044 | 0.115 | 38.1 | 17.95 | 0.044 | 0.119 | 35.0 | 5.91 | 0.007 | 0.008 | 88.8 | 10.49 | 0.006 | 0.011 | 78.2 |

**Note:** SoSt indicates the scenario with both strong outcome and treatment (Scenario 1). $\hat{\theta}_{a,a’}\left( a \right)$, $\hat{\theta}_{a,a’}\left( a’ \right)$ separately represent direct effects under treatment and non-treatment, and $\hat{\delta}_{a,a’}\left( a \right)$, $\hat{\delta}_{a,a’}\left( a’ \right)$ separately represent indirect effects under treatment and non-treatment. “Bias (%)”, “SD”, “RMSE”, and “CP” respectively report the average relative absolute bias, standard deviation, root mean squared error, and coverage probability of the effects across all treatment values *a* ∈ {-1, -0.9, ..., -0.1} ∪ {0.1, ..., 0.9, 1} and *a’*=0. Results of the GOAL, Adaptive LASSO, and LASSO methods are all based on a gamma convergence of 2. The kernel bandwidth is set to *C*·*n*^-0.25^ with *C*=2.34.

**Abbreviations**: LASSO, the least absolute shrinkage and selection operator; GOAL, generalized outcome-adaptive LASSO; AdaLASSO, adaptive LASSO.

## Table B. Mediation effects estimated by weighting with a parametric generalized propensity score based on different covariate sets under Scenario 2 (SoWt) using the semiparametric kernel bandwidth.

| **Sample Size** | **Methods** | **Correlation (rho)** | ${\hat{\boldsymbol{\theta}}}_{\boldsymbol{a,a’}}\left( \boldsymbol{a} \right)$ | | | | ${\hat{\boldsymbol{\theta}}}_{\boldsymbol{a,a’}}\left( \boldsymbol{a’} \right)$ | | | | ${\hat{\boldsymbol{\delta}}}_{\boldsymbol{a,a’}}\left( \boldsymbol{a} \right)$ | | | | ${\hat{\boldsymbol{\delta}}}_{\boldsymbol{a,a’}}\left( \boldsymbol{a’} \right)$ | | | |
| --- | --- | --- | --- | --- | --- | --- | --- | --- | --- | --- | --- | --- | --- | --- | --- | --- | --- | --- |
|  |  |  | **Bias (%)** | **SD** | **RMSE** | **CP (%)** | **Bias (%)** | **SD** | **RMSE** | **CP (%)** | **Bias (%)** | **SD** | **RMSE** | **CP (%)** | **Bias (%)** | **SD** | **RMSE** | **CP (%)** |
| ***n*=2000** | GOAL | 0 | 7.73 | 0.046 | 0.065 | 82.7 | 7.63 | 0.046 | 0.065 | 83.0 | 10.25 | 0.009 | 0.010 | 92.5 | 11.35 | 0.008 | 0.010 | 93.0 |
|  |  | 0.3 | 14.79 | 0.051 | 0.095 | 66.4 | 14.95 | 0.051 | 0.096 | 66.2 | 6.48 | 0.009 | 0.009 | 94.6 | 5.28 | 0.009 | 0.009 | 94.7 |
|  | AdaLASSO | 0 | 13.56 | 0.051 | 0.089 | 71.7 | 13.30 | 0.051 | 0.088 | 72.0 | 4.52 | 0.011 | 0.011 | 96.2 | 4.42 | 0.010 | 0.010 | 96.6 |
|  |  | 0.3 | 24.25 | 0.056 | 0.141 | 40.2 | 24.56 | 0.056 | 0.142 | 38.6 | 12.15 | 0.011 | 0.012 | 92.5 | 15.66 | 0.011 | 0.013 | 86.2 |
|  | LASSO | 0 | 13.41 | 0.051 | 0.089 | 71.1 | 13.14 | 0.051 | 0.088 | 72.2 | 4.20 | 0.011 | 0.010 | 95.6 | 3.21 | 0.010 | 0.010 | 95.9 |
|  |  | 0.3 | 25.47 | 0.056 | 0.146 | 36.0 | 25.75 | 0.056 | 0.147 | 33.4 | 6.62 | 0.011 | 0.010 | 96.8 | 9.71 | 0.010 | 0.011 | 92.8 |
|  | Benchmark (True) | 0 | 8.48 | 0.050 | 0.090 | 84.9 | 7.43 | 0.050 | 0.089 | 85.0 | 58.29 | 0.009 | 0.009 | 73.9 | 46.67 | 0.009 | 0.009 | 84.4 |
|  |  | 0.3 | 3.04 | 0.055 | 0.143 | 94.4 | 1.57 | 0.055 | 0.144 | 95.8 | 210.87 | 0.010 | 0.010 | 3.4 | 241.39 | 0.009 | 0.010 | 1.6 |
|  | Benchmark (Outcome) | 0 | 7.18 | 0.046 | 0.062 | 85.1 | 7.09 | 0.046 | 0.062 | 85.2 | 10.77 | 0.008 | 0.009 | 91.4 | 11.81 | 0.008 | 0.010 | 89.5 |
|  |  | 0.3 | 12.46 | 0.050 | 0.086 | 71.8 | 12.52 | 0.050 | 0.085 | 73.1 | 9.05 | 0.009 | 0.010 | 93.2 | 8.49 | 0.009 | 0.010 | 95.4 |
|  | Benchmark (True+Outcome) | 0 | 8.48 | 0.050 | 0.090 | 84.7 | 7.44 | 0.050 | 0.089 | 85.0 | 58.68 | 0.009 | 0.009 | 75.2 | 47.11 | 0.009 | 0.009 | 84.0 |
|  |  | 0.3 | 3.57 | 0.055 | 0.142 | 93.2 | 5.55 | 0.055 | 0.143 | 92.2 | 164.45 | 0.010 | 0.010 | 12.3 | 195.03 | 0.009 | 0.010 | 7.5 |
|  | Benchmark (Full) | 0 | 13.79 | 0.050 | 0.090 | 69.3 | 13.61 | 0.050 | 0.089 | 69.9 | 5.18 | 0.009 | 0.009 | 96.2 | 6.57 | 0.009 | 0.009 | 98.0 |
|  |  | 0.3 | 24.10 | 0.055 | 0.142 | 39.7 | 24.40 | 0.055 | 0.143 | 37.7 | 8.44 | 0.010 | 0.010 | 94.3 | 11.73 | 0.009 | 0.010 | 92.5 |
| ***n*=5000** | GOAL | 0 | 3.04 | 0.036 | 0.04 | 92.7 | 2.88 | 0.036 | 0.039 | 92.7 | 5.78 | 0.006 | 0.007 | 89.8 | 7.58 | 0.006 | 0.007 | 87.2 |
|  |  | 0.3 | 11.93 | 0.041 | 0.072 | 67.4 | 12.01 | 0.041 | 0.074 | 67.2 | 4.55 | 0.006 | 0.007 | 92.3 | 4.64 | 0.006 | 0.006 | 91.2 |
|  | AdaLASSO | 0 | 7.62 | 0.040 | 0.058 | 81.9 | 7.41 | 0.040 | 0.057 | 83.2 | 3.19 | 0.007 | 0.007 | 93.9 | 1.95 | 0.007 | 0.007 | 94.8 |
|  |  | 0.3 | 19.12 | 0.044 | 0.113 | 38.1 | 19.57 | 0.044 | 0.116 | 35.0 | 11.26 | 0.007 | 0.009 | 85.6 | 16.34 | 0.007 | 0.011 | 75.8 |
|  | LASSO | 0 | 7.50 | 0.040 | 0.058 | 82.5 | 7.30 | 0.040 | 0.057 | 83.8 | 3.44 | 0.007 | 0.007 | 93.6 | 1.67 | 0.006 | 0.007 | 94.4 |
|  |  | 0.3 | 20.01 | 0.044 | 0.114 | 33.7 | 20.47 | 0.043 | 0.117 | 31.3 | 6.03 | 0.007 | 0.008 | 91.6 | 11.11 | 0.007 | 0.01 | 84.4 |
|  | Benchmark (True) | 0 | 3.69 | 0.039 | 0.059 | 91.7 | 2.85 | 0.039 | 0.058 | 93.7 | 40.08 | 0.006 | 0.006 | 77.7 | 30.83 | 0.006 | 0.006 | 85.4 |
|  |  | 0.3 | 6.72 | 0.043 | 0.115 | 84.1 | 3.04 | 0.043 | 0.119 | 91.7 | 188.38 | 0.007 | 0.007 | 0.7 | 229.31 | 0.006 | 0.009 | 0.0 |
|  | Benchmark (Outcome) | 0 | 2.87 | 0.036 | 0.039 | 92.7 | 2.73 | 0.036 | 0.038 | 92.9 | 6.38 | 0.006 | 0.007 | 89.6 | 8.03 | 0.005 | 0.007 | 88.0 |
|  |  | 0.3 | 10.17 | 0.040 | 0.066 | 75.6 | 10.10 | 0.040 | 0.066 | 75.0 | 6.85 | 0.006 | 0.007 | 90.6 | 7.63 | 0.006 | 0.007 | 88.2 |
|  | Benchmark (True+Outcome) | 0 | 3.68 | 0.039 | 0.06 | 91.9 | 2.85 | 0.039 | 0.058 | 93.8 | 40.74 | 0.006 | 0.006 | 76.2 | 31.51 | 0.006 | 0.006 | 83.4 |
|  |  | 0.3 | 3.67 | 0.043 | 0.115 | 89.8 | 1.22 | 0.043 | 0.119 | 93.2 | 143.28 | 0.007 | 0.007 | 5.0 | 182.43 | 0.006 | 0.009 | 0.1 |
|  | Benchmark (Full) | 0 | 7.98 | 0.040 | 0.059 | 80.6 | 7.82 | 0.040 | 0.058 | 82.1 | 3.57 | 0.006 | 0.006 | 94.9 | 4.09 | 0.006 | 0.006 | 95.0 |
|  |  | 0.3 | 20.20 | 0.043 | 0.115 | 32.8 | 20.58 | 0.043 | 0.119 | 30.4 | 5.70 | 0.007 | 0.007 | 94.1 | 9.92 | 0.006 | 0.009 | 85.2 |

**Note:** SoWt indicates the scenario with strong outcome and weak treatment (Scenario 2). $\hat{\theta}_{a,a’}\left( a \right)$, $\hat{\theta}_{a,a’}\left( a’ \right)$ separately represent direct effects under treatment and non-treatment, and $\hat{\delta}_{a,a’}\left( a \right)$, $\hat{\delta}_{a,a’}\left( a’ \right)$ separately represent indirect effects under treatment and non-treatment. “Bias (%)”, “SD”, “RMSE”, and “CP” respectively report the average relative absolute bias, standard deviation, root mean squared error, and coverage probability of the effects across all treatment values *a* ∈ {-1, -0.9, ..., -0.1} ∪ {0.1, ..., 0.9, 1} and *a’*=0. Results of the GOAL, Adaptive LASSO, and LASSO methods are all based on a gamma convergence of 2. The standard kernel bandwidth is set to *C*·*n*^-0.25^ with *C*=2.34.

**Abbreviations**: LASSO, the least absolute shrinkage and selection operator; GOAL, generalized outcome-adaptive LASSO; AdaLASSO, adaptive LASSO.

## Table C. Mediation effects estimated by weighting with a parametric generalized propensity score based on different covariate sets under Scenario 3 (WoSt) using the semiparametric kernel bandwidth.

| **Sample Size** | **Methods** | **Correlation (rho)** | ${\hat{\boldsymbol{\theta}}}_{\boldsymbol{a,a’}}\left( \boldsymbol{a} \right)$ | | | | ${\hat{\boldsymbol{\theta}}}_{\boldsymbol{a,a’}}\left( \boldsymbol{a’} \right)$ | | | | ${\hat{\boldsymbol{\delta}}}_{\boldsymbol{a,a’}}\left( \boldsymbol{a} \right)$ | | | | ${\hat{\boldsymbol{\delta}}}_{\boldsymbol{a,a’}}\left( \boldsymbol{a’} \right)$ | | | |
| --- | --- | --- | --- | --- | --- | --- | --- | --- | --- | --- | --- | --- | --- | --- | --- | --- | --- | --- |
|  |  |  | **Bias (%)** | **SD** | **RMSE** | **CP (%)** | **Bias (%)** | **SD** | **RMSE** | **CP (%)** | **Bias (%)** | **SD** | **RMSE** | **CP (%)** | **Bias (%)** | **SD** | **RMSE** | **CP (%)** |
| ***n*=2000** | GOAL | 0 | 8.24 | 0.045 | 0.066 | 81.7 | 8.13 | 0.045 | 0.066 | 80.3 | 10.36 | 0.009 | 0.009 | 92.3 | 11.63 | 0.008 | 0.009 | 91.2 |
|  |  | 0.3 | 13.15 | 0.051 | 0.090 | 69.5 | 13.27 | 0.051 | 0.091 | 69.6 | 6.42 | 0.009 | 0.009 | 94.2 | 5.69 | 0.009 | 0.008 | 95.5 |
|  | AdaLASSO | 0 | 14.64 | 0.051 | 0.094 | 67.5 | 14.42 | 0.051 | 0.092 | 68.2 | 4.79 | 0.010 | 0.010 | 95.9 | 5.90 | 0.009 | 0.009 | 96.9 |
|  |  | 0.3 | 22.61 | 0.056 | 0.131 | 45.3 | 22.89 | 0.056 | 0.132 | 44.2 | 10.30 | 0.010 | 0.011 | 94.7 | 13.46 | 0.010 | 0.012 | 90.6 |
|  | LASSO | 0 | 14.60 | 0.051 | 0.094 | 68.0 | 14.38 | 0.050 | 0.092 | 67.8 | 4.79 | 0.010 | 0.010 | 95.9 | 6.01 | 0.009 | 0.009 | 96.4 |
|  |  | 0.3 | 23.23 | 0.055 | 0.136 | 42.3 | 23.45 | 0.055 | 0.138 | 40.9 | 10.40 | 0.010 | 0.010 | 95.6 | 12.92 | 0.010 | 0.011 | 91.5 |
|  | Benchmark (True) | 0 | 8.20 | 0.050 | 0.093 | 85.0 | 7.53 | 0.050 | 0.092 | 84.1 | 63.87 | 0.009 | 0.009 | 71.8 | 56.45 | 0.009 | 0.008 | 79.0 |
|  |  | 0.3 | 5.34 | 0.054 | 0.133 | 91.4 | 2.35 | 0.054 | 0.134 | 95.0 | 201.98 | 0.009 | 0.010 | 4.4 | 235.25 | 0.009 | 0.010 | 3.0 |
|  | Benchmark (Outcome) | 0 | 7.74 | 0.045 | 0.063 | 82.9 | 7.63 | 0.045 | 0.063 | 82.3 | 10.70 | 0.008 | 0.009 | 91.9 | 11.92 | 0.008 | 0.009 | 91.0 |
|  |  | 0.3 | 11.00 | 0.050 | 0.080 | 75.6 | 11.06 | 0.050 | 0.080 | 75.5 | 8.21 | 0.009 | 0.010 | 93.7 | 7.52 | 0.008 | 0.009 | 96.7 |
|  | Benchmark (True+Outcome) | 0 | 8.20 | 0.049 | 0.093 | 85.5 | 7.53 | 0.050 | 0.092 | 84.2 | 64.23 | 0.009 | 0.009 | 72.2 | 56.83 | 0.009 | 0.008 | 79.1 |
|  |  | 0.3 | 1.45 | 0.054 | 0.133 | 94.3 | 1.94 | 0.054 | 0.133 | 95.3 | 156.34 | 0.009 | 0.010 | 15.6 | 189.03 | 0.009 | 0.010 | 8.2 |
|  | Benchmark (Full) | 0 | 14.69 | 0.050 | 0.093 | 66.3 | 14.51 | 0.050 | 0.092 | 67.0 | 4.97 | 0.009 | 0.009 | 96.7 | 5.80 | 0.009 | 0.008 | 97.5 |
|  |  | 0.3 | 21.83 | 0.055 | 0.133 | 46.6 | 22.09 | 0.055 | 0.133 | 45.7 | 10.36 | 0.010 | 0.010 | 93.5 | 13.29 | 0.009 | 0.010 | 92.5 |
| ***n*=5000** | GOAL | 0 | 3.37 | 0.036 | 0.042 | 91.1 | 3.24 | 0.036 | 0.041 | 91.8 | 5.13 | 0.006 | 0.007 | 89.4 | 6.66 | 0.006 | 0.007 | 88.7 |
|  |  | 0.3 | 9.59 | 0.041 | 0.065 | 75.6 | 9.74 | 0.040 | 0.066 | 73.8 | 3.39 | 0.006 | 0.007 | 92.3 | 4.00 | 0.006 | 0.006 | 92.0 |
|  | AdaLASSO | 0 | 8.70 | 0.040 | 0.063 | 78.0 | 8.51 | 0.040 | 0.061 | 79.3 | 3.23 | 0.007 | 0.006 | 96.3 | 3.40 | 0.006 | 0.006 | 96.3 |
|  |  | 0.3 | 16.82 | 0.043 | 0.105 | 48.6 | 17.16 | 0.043 | 0.107 | 46.5 | 9.12 | 0.007 | 0.008 | 88.0 | 12.89 | 0.007 | 0.01 | 82.9 |
|  | LASSO | 0 | 8.62 | 0.040 | 0.062 | 78.4 | 8.46 | 0.039 | 0.061 | 79.2 | 3.04 | 0.007 | 0.006 | 94.5 | 2.94 | 0.006 | 0.006 | 94.0 |
|  |  | 0.3 | 17.34 | 0.043 | 0.103 | 46.5 | 17.71 | 0.043 | 0.105 | 43.0 | 7.83 | 0.007 | 0.008 | 89.5 | 11.90 | 0.007 | 0.009 | 83.9 |
|  | Benchmark (True) | 0 | 2.98 | 0.039 | 0.063 | 91.3 | 2.84 | 0.039 | 0.062 | 92.6 | 44.94 | 0.006 | 0.006 | 74.2 | 43.40 | 0.006 | 0.006 | 77.1 |
|  |  | 0.3 | 9.74 | 0.043 | 0.1 | 80.5 | 6.63 | 0.043 | 0.103 | 88.8 | 175.19 | 0.006 | 0.008 | 0.6 | 209.74 | 0.006 | 0.009 | 0.0 |
|  | Benchmark (Outcome) | 0 | 3.22 | 0.036 | 0.041 | 90.9 | 3.08 | 0.036 | 0.041 | 91.7 | 6.29 | 0.006 | 0.007 | 89.8 | 7.79 | 0.005 | 0.007 | 88.1 |
|  |  | 0.3 | 8.42 | 0.039 | 0.061 | 81.3 | 8.39 | 0.040 | 0.061 | 80.8 | 6.25 | 0.006 | 0.007 | 91.1 | 6.51 | 0.006 | 0.006 | 87.6 |
|  | Benchmark (True+Outcome) | 0 | 2.97 | 0.039 | 0.063 | 91.1 | 2.83 | 0.039 | 0.062 | 92.5 | 45.62 | 0.006 | 0.006 | 73.6 | 44.12 | 0.006 | 0.006 | 76.4 |
|  |  | 0.3 | 5.31 | 0.043 | 0.105 | 89.7 | 2.34 | 0.043 | 0.108 | 93.7 | 131.24 | 0.006 | 0.008 | 7.0 | 164.27 | 0.006 | 0.009 | 0.2 |
|  | Benchmark (Full) | 0 | 8.77 | 0.039 | 0.063 | 78.0 | 8.65 | 0.039 | 0.062 | 78.7 | 3.25 | 0.006 | 0.006 | 95.4 | 3.04 | 0.006 | 0.006 | 94.3 |
|  |  | 0.3 | 17.55 | 0.043 | 0.105 | 43.8 | 17.85 | 0.043 | 0.108 | 41.9 | 6.88 | 0.006 | 0.008 | 92.4 | 10.24 | 0.006 | 0.009 | 84.1 |

**Note:** WoSt indicates the scenario with weak outcome and strong treatment (Scenario 3). $\hat{\theta}_{a,a’}\left( a \right)$, $\hat{\theta}_{a,a’}\left( a’ \right)$ separately represent direct effects under treatment and non-treatment, and $\hat{\delta}_{a,a’}\left( a \right)$, $\hat{\delta}_{a,a’}\left( a’ \right)$ separately represent indirect effects under treatment and non-treatment. “Bias (%)”, “SD”, “RMSE”, and “CP” respectively report the average relative absolute bias, standard deviation, root mean squared error, and coverage probability of the effects across all treatment values *a* ∈ {-1, -0.9, ..., -0.1} ∪ {0.1, ..., 0.9, 1} and *a’*=0. Results of the GOAL, Adaptive LASSO, and LASSO methods are all based on a gamma convergence of 2. The standard kernel bandwidth is set to *C*·*n*^-0.25^ with *C*=2.34.

**Abbreviations**: LASSO, the least absolute shrinkage and selection operator; GOAL, generalized outcome-adaptive LASSO; AdaLASSO, adaptive LASSO.

## Table D. Bandwidth diagnostic results in simulation scenarios.

| **Bandwidth** | **Scenarios** | **Eff. window half-width** | **ESS (Weight)** | **ESS (Kernel)** | **ESS ratio** | **ESS-weighted mean SMD** | **SMD** | **CV (active)** |
| --- | --- | --- | --- | --- | --- | --- | --- | --- |
| *h_wp_* | S1 (SoSt) | 0.78 | 561.9 | 593.6 | 0.958 | 0.06 | 0.08 | 0.51 |
|  | S2 (SoWt) | 0.78 | 569.6 | 597.5 | 0.964 | 0.06 | 0.08 | 0.50 |
|  | S3 (WoSt) | 0.78 | 561.9 | 593.6 | 0.958 | 0.07 | 0.09 | 0.47 |
| *h_wp_* _us_ | S1 (SoSt) | 0.39 | 284.5 | 300.5 | 0.960 | 0.09 | 0.11 | 0.52 |
|  | S2 (SoWt) | 0.39 | 288.0 | 302.3 | 0.965 | 0.08 | 0.11 | 0.51 |
|  | S3 (WoSt) | 0.39 | 284.4 | 300.5 | 0.959 | 0.09 | 0.12 | 0.52 |
| *h_ROT_* | S1 (SoSt) | 1.65 | 1094.9 | 1156.0 | 0.958 | 0.04 | 0.07 | 0.48 |
|  | S2 (SoWt) | 1.63 | 1099.3 | 1152.8 | 0.964 | 0.04 | 0.06 | 0.47 |
|  | S3 (WoSt) | 1.65 | 1094.7 | 1156.1 | 0.958 | 0.04 | 0.07 | 0.48 |

**Note:** The *h_wp_* corresponds to the kernel bandwidth that is set to *C*·*n*^-0.25^ with *C*=2.34 in the weighted semiparametric estimator. The *h_wp_* _us_ corresponds to the undersmoothing version of the *h_wp_* kernel bandwidth, specified by halving it as *C*·*n*^-0.25^/2. *h_ROT_* corresponds to the rule-of-thumb (ROT) kernel bandwidth as *C* · sd(*A*)·*n*^-1/5^ with *C* =2.34. Eff. window half-width represents the effective kernel window half-width, which is defined as *h*·$\sqrt{5}$, where $\sqrt{5}$ reflects the relationship between the bandwidth parameter and the support half-width of the second-order Epanechnikov kernel. The minimum values of ESS (weight), ESS (kernel), and the ESS ratio, and the maximum values of SMD-related metrics and CV across the exposure range are reported. S1 (SoSt) indicates the Scenario 1 with both strong outcome and treatment, S2 (SoWt) indicates the Scenario 2 with strong outcome and weak treatment, and S3 (WoSt) indicates the Scenario 3 with weak outcome and strong treatment.

**Abbreviations**: ESS, effective sample size; SMD, standardized mean difference; CV, coefficient of variation.

## Table E. Stability check of bootstrap-based variance estimates.

| **Methods** | **Bootstrap Replicates** | **Average Standard Deviation** | | | |
| --- | --- | --- | --- | --- | --- |
|  |  | **NDE (treatment)** | **NDE (non-treatment)** | **NIE (treatment)** | **NIE (non-treatment)** |
| **GOAL** | 200 | 0.085 | 0.085 | 0.011 | 0.011 |
|  | 500 | 0.085 | 0.085 | 0.011 | 0.011 |
|  | 1000 | 0.085 | 0.085 | 0.011 | 0.011 |
| **AdaLASSO** | 200 | 0.094 | 0.093 | 0.013 | 0.012 |
|  | 500 | 0.094 | 0.093 | 0.013 | 0.012 |
|  | 1000 | 0.094 | 0.093 | 0.013 | 0.012 |
| **LASSO** | 200 | 0.094 | 0.093 | 0.013 | 0.012 |
|  | 500 | 0.094 | 0.093 | 0.013 | 0.012 |
|  | 1000 | 0.094 | 0.093 | 0.013 | 0.012 |
| **Benchmark (Full)** | 200 | 0.093 | 0.093 | 0.013 | 0.013 |
|  | 500 | 0.093 | 0.093 | 0.012 | 0.011 |
|  | 1000 | 0.093 | 0.093 | 0.013 | 0.013 |

**Notes:** The sensitivity analysis is conducted using the simulation data of (*n*, *p*) = (2000, 100) with covariate correlation of 0 under Scenario 1. The estimate procedure is based on the undersmoothing bandwidth *h* of (*C*·*n*^-0.25^)/2, where *C*=2.34.

**Abbreviations**: LASSO, the least absolute shrinkage and selection operator; GOAL, generalized outcome-adaptive LASSO; AdaLASSO, adaptive LASSO; NDE, natural direct effect; NIE, natural indirect effect.

## Table F. Robustness check of interactive and nonlinear outcome models using the GOAL-based method.

| **Scenarios** | **Model form** | ${\hat{\boldsymbol{\theta}}}_{\boldsymbol{a,a’}}\left( \boldsymbol{a} \right)$ | | |  | ${\hat{\boldsymbol{\theta}}}_{\boldsymbol{a,a’}}\left( \boldsymbol{a’} \right)$ | | |  | ${\hat{\boldsymbol{\delta}}}_{\boldsymbol{a,a’}}\left( \boldsymbol{a} \right)$ | | |  | ${\hat{\boldsymbol{\delta}}}_{\boldsymbol{a,a’}}\left( \boldsymbol{a’} \right)$ | | |
| --- | --- | --- | --- | --- | --- | --- | --- | --- | --- | --- | --- | --- | --- | --- | --- | --- |
|  |  | **Bias (%)** | **SD** | **RMSE** |  | **Bias (%)** | **SD** | **RMSE** |  | **Bias (%)** | **SD** | **RMSE** |  | **Bias (%)** | **SD** | **RMSE** |
| **Scenario 1 (SoSt)** | original (reference) | 9.08 | 0.046 | 0.070 |  | 8.97 | 0.046 | 0.070 |  | 10.51 | 0.009 | 0.009 |  | 11.75 | 0.008 | 0.009 |
|  | interactive | 9.23 | 0.048 | 0.071 |  | 9.10 | 0.048 | 0.071 |  | 9.85 | 0.009 | 0.010 |  | 11.39 | 0.008 | 0.009 |
|  | nonlinear | 9.84 | 0.049 | 0.075 |  | 9.50 | 0.049 | 0.074 |  | 11.17 | 0.010 | 0.010 |  | 11.12 | 0.009 | 0.010 |
| **Scenario 2 (SoWt)** | original (reference) | 7.73 | 0.046 | 0.065 |  | 7.63 | 0.046 | 0.065 |  | 10.25 | 0.009 | 0.010 |  | 11.35 | 0.008 | 0.010 |
|  | interactive | 7.82 | 0.047 | 0.066 |  | 7.69 | 0.047 | 0.066 |  | 9.70 | 0.009 | 0.010 |  | 10.91 | 0.008 | 0.010 |
|  | nonlinear | 8.42 | 0.048 | 0.069 |  | 8.07 | 0.048 | 0.069 |  | 8.99 | 0.010 | 0.010 |  | 11.08 | 0.009 | 0.010 |
| **Scenario 3 (WoSt)** | original (reference) | 8.24 | 0.045 | 0.066 |  | 8.13 | 0.045 | 0.066 |  | 10.36 | 0.009 | 0.009 |  | 11.63 | 0.008 | 0.009 |
|  | interaction | 8.29 | 0.047 | 0.067 |  | 8.14 | 0.047 | 0.067 |  | 10.16 | 0.009 | 0.011 |  | 11.98 | 0.009 | 0.010 |
|  | non-linearity | 8.73 | 0.048 | 0.069 |  | 8.38 | 0.048 | 0.069 |  | 9.46 | 0.010 | 0.011 |  | 11.90 | 0.009 | 0.011 |

**Notes:** $\hat{\theta}_{a,a’}\left( a \right)$, $\hat{\theta}_{a,a’}\left( a’ \right)$ separately represent direct effects under treatment and non-treatment, and $\hat{\delta}_{a,a’}\left( a \right)$, $\hat{\delta}_{a,a’}\left( a’ \right)$ separately represent indirect effects under treatment and non-treatment. “Bias (%)”, “SD”, and “RMSE” respectively report the average relative bias, standard deviation, and root mean squared error of the effects across all treatment values *a* ∈ {-1, -0.9, ..., -0.1} ∪ {0.1, ..., 0.9, 1} and *a’*=0. The sensitivity analysis is performed using the kernel bandwidth *h*=*C* ·*n*^-0.25^ under sample size and covariate dimension (*n*, *p*) of (2000, 100) and covariate correlation *ρ*=0.

**Abbreviations:** GOAL, generalized outcome-adaptive LASSO; SoSt, the scenario with both strong outcome and treatment; SoWt, the scenario with strong outcome and weak treatment; WoSt, the scenario with weak outcome and strong treatment; SD, standard deviation; RMSE, root mean squared error.

## Table G. Detailed coverage probability performance across the continuous exposure range.

| **Scenarios** | **Correlation (rho)** | ${\hat{\boldsymbol{\theta}}}_{\boldsymbol{a,a’}}\left( \boldsymbol{a} \right)$ | | ${\hat{\boldsymbol{\theta}}}_{\boldsymbol{a,a’}}\left( \boldsymbol{a’} \right)$ | | ${\hat{\boldsymbol{\delta}}}_{\boldsymbol{a,a’}}\left( \boldsymbol{a} \right)$ | | ${\hat{\boldsymbol{\delta}}}_{\boldsymbol{a,a’}}\left( \boldsymbol{a’} \right)$ | |
| --- | --- | --- | --- | --- | --- | --- | --- | --- | --- |
|  |  | **Minimal CP** | **Average CP (95%CI)** | **Minimal CP** | **Average CP (95%CI)** | **Minimal CP** | **Average CP (95%CI)** | **Minimal CP** | **Average CP (95%CI)** |
| **Scenario 1 (SoSt)** | 0 | 87.0 | 90.3 (87.0, 93.6) | 88.0 | 90.6 (87.2, 94.0) | 92.2 | 96.1 (93.4, 98.8) | 96.1 | 98.0 (96.9, 99.1) |
|  | 0.3 | 90.3 | 93.2 (90.3, 96.1) | 89.3 | 93.1 (89.9, 96.3) | 92.0 | 95.3 (92.2, 98.4) | 90.0 | 92.8 (89.9, 95.7) |
| **Scenario 2 (SoWt)** | 0 | 89.0 | 91.8 (89.3, 94.3) | 89.5 | 92.1 (89.7, 94.5) | 93.5 | 96.3 (93.1, 99.5) | 92.2 | 96.9 (94.5, 99.3) |
|  | 0.3 | 88.0 | 91.8 (88.2, 95.4) | 88.5 | 91.5 (87.8, 95.2) | 93.0 | 96.0 (92.1, 99.9) | 90.9 | 95.0 (91.6, 98.4) |
| **Scenario 3 (WoSt)** | 0 | 86.0 | 90.9 (86.0, 95.8) | 89.5 | 91.2 (88.0, 94.4) | 92.5 | 95.8 (92.9, 98.7) | 93.5 | 96.1 (94.3, 97.9) |
|  | 0.3 | 89.6 | 93.5 (90.2, 96.8) | 89.3 | 93.5 (89.5, 97.5) | 92.0 | 95.8 (92.7, 98.9) | 91.0 | 94.1 (91.4, 96.8) |

**Notes:** $\hat{\theta}_{a,a’}\left( a \right)$, $\hat{\theta}_{a,a’}\left( a’ \right)$ separately represent direct effects under treatment and non-treatment, and $\hat{\delta}_{a,a’}\left( a \right)$, $\hat{\delta}_{a,a’}\left( a’ \right)$ separately represent indirect effects under treatment and non-treatment. The sensitivity analysis is conducted using the proposed GOAL-based method based on the undersmoothing bandwidth *h*=(*C*·*n*^-0.25^)/2, under sample size and covariate dimension (*n*, *p*) of (2000, 100). The involved CP metrics are all measured across the full exposure evaluation range.

**Abbreviations**: GOAL, generalized outcome-adaptive LASSO; CP, coverage probability; SoSt, the scenario with both strong outcome and treatment; SoWt, the scenario with strong outcome and weak treatment; WoSt, the scenario with weak outcome and strong treatment.

## Table H. Robustness test regarding the method sparsity of the proposed GOAL-based method.

| **Effects** | **Sparsity** | **Bias (%)** | **SD** | **RMSE** | **CP (%)** |
| --- | --- | --- | --- | --- | --- |
| **NDE (treatment)** | s = 2 (original) | 6.33 | 0.085 | 0.098 | 90.3 |
|  | s = 5 | 8.10 | 0.093 | 0.102 | 91.8 |
|  | s = 10 | 9.89 | 0.107 | 0.117 | 92.2 |
| **NDE (non-treatment)** | s = 2 (original) | 6.09 | 0.085 | 0.098 | 90.6 |
|  | s = 5 | 8.01 | 0.093 | 0.103 | 92.0 |
|  | s = 10 | 10.07 | 0.107 | 0.118 | 92.3 |
| **NIE (treatment)** | s = 2 (original) | 3.15 | 0.011 | 0.011 | 96.1 |
|  | s = 5 | 1.76 | 0.012 | 0.012 | 95.2 |
|  | s = 10 | 1.98 | 0.014 | 0.014 | 94.1 |
| **NIE (non-treatment)** | s = 2 (original) | 5.46 | 0.011 | 0.009 | 98.0 |
|  | s = 5 | 2.49 | 0.011 | 0.011 | 95.8 |
|  | s = 10 | 2.52 | 0.013 | 0.011 | 98.5 |

**Notes:** The sensitivity analysis is conducted using the simulation data of (*n*, *p*) = (2000, 100) with covariate correlation of 0 under Scenario 1 using the proposed GOAL-based method. The estimate procedure is based on the undersmoothing bandwidth. “s” indicates the variable numbers of confounders or prognostic variables, with a smaller number indicating a strong sparsity of the model.

**Abbreviations**: GOAL, generalized outcome-adaptive LASSO; SD, standard deviation; RMSE, root mean squared error; CP, coverage probability; NDE, natural direct effect; NIE, natural indirect effect.

## Table I. Investigation of estimation performance of the GOAL-based method under varying covariate-to-sample size ratios.

| $\boldsymbol{p/n}$ **ratios** | ${\hat{\boldsymbol{\theta}}}_{\boldsymbol{a,a’}}\left( \boldsymbol{a} \right)$ | | | | ${\hat{\boldsymbol{\theta}}}_{\boldsymbol{a,a’}}\left( \boldsymbol{a’} \right)$ | | | | ${\hat{\boldsymbol{\delta}}}_{\boldsymbol{a,a’}}\left( \boldsymbol{a} \right)$ | | | | ${\hat{\boldsymbol{\delta}}}_{\boldsymbol{a,a’}}\left( \boldsymbol{a’} \right)$ | | | |
| --- | --- | --- | --- | --- | --- | --- | --- | --- | --- | --- | --- | --- | --- | --- | --- | --- |
|  | **Bias (%)** | **SD** | **RMSE** | **CP (%)** | **Bias (%)** | **SD** | **RMSE** | **CP (%)** | **Bias (%)** | **SD** | **RMSE** | **CP (%)** | **Bias (%)** | **SD** | **RMSE** | **CP (%)** |
| **ratio=0.1** | 2.96 | 0.085 | 0.088 | 93.0 | 3.04 | 0.085 | 0.089 | 93.2 | 1.86 | 0.011 | 0.011 | 95.0 | 0.67 | 0.011 | 0.011 | 95.3 |
| **ratio=0.2** | 4.53 | 0.085 | 0.094 | 93.0 | 4.53 | 0.086 | 0.094 | 93.3 | 2.98 | 0.012 | 0.012 | 94.4 | 2.35 | 0.011 | 0.011 | 95.8 |
| **ratio=0.3** | 2.98 | 0.086 | 0.086 | 94.3 | 2.82 | 0.086 | 0.085 | 94.0 | 1.76 | 0.011 | 0.012 | 94.2 | 2.56 | 0.011 | 0.011 | 96.6 |
| **ratio=0.4** | 3.95 | 0.085 | 0.095 | 92.1 | 4.14 | 0.085 | 0.096 | 92.1 | 1.28 | 0.011 | 0.011 | 95.8 | 1.87 | 0.011 | 0.011 | 95.0 |
| **ratio=0.5** | 3.74 | 0.087 | 0.089 | 94.1 | 3.63 | 0.087 | 0.089 | 94.1 | 1.98 | 0.012 | 0.012 | 94.3 | 0.90 | 0.011 | 0.011 | 93.8 |
| **ratio=0.6** | 2.45 | 0.088 | 0.083 | 96.7 | 2.65 | 0.087 | 0.083 | 96.8 | 3.40 | 0.013 | 0.012 | 95.0 | 5.72 | 0.013 | 0.012 | 97.3 |
| **ratio=0.7** | 1.78 | 0.093 | 0.086 | 96.6 | 2.09 | 0.090 | 0.088 | 96.4 | 3.34 | 0.018 | 0.017 | 94.3 | 6.81 | 0.017 | 0.016 | 95.8 |
| **ratio=0.8** | 2.32 | 0.102 | 0.104 | 95.2 | 1.99 | 0.098 | 0.093 | 95.0 | 2.76 | 0.023 | 0.020 | 93.7 | 4.24 | 0.023 | 0.031 | 96.0 |
| **ratio=0.9** | 3.11 | 0.092 | 0.098 | 93.0 | 3.09 | 0.090 | 0.098 | 93.1 | 6.06 | 0.016 | 0.016 | 92.3 | 1.01 | 0.016 | 0.021 | 93.8 |

**Notes:** $\hat{\theta}_{a,a’}\left( a \right)$, $\hat{\theta}_{a,a’}\left( a’ \right)$ separately represent direct effects under treatment and non-treatment, and $\hat{\delta}_{a,a’}\left( a \right)$, $\hat{\delta}_{a,a’}\left( a’ \right)$ separately represent indirect effects under treatment and non-treatment. “Bias (%)”, “SD”, “RMSE”, and “CP” respectively report the average relative bias, standard deviation, root mean squared error, and coverage probability of the effects across all treatment values *a* ∈ {-1, -0.9, ..., -0.1} ∪ {0.1, ..., 0.9, 1} and *a’*=0. The analysis is performed using the undersmoothing kernel bandwidth *h* = (*C ·n*^-0.25^)/2 under sample size and covariate dimension (*n*, *p*) of (2000, 100) with covariate correlation of 0 under Scenario 1.

**Abbreviations**: GOAL, generalized outcome-adaptive LASSO.

## Table J. Simulation performance of the GOAL-based estimation under normally distributed errors.

| **Scenarios** | **Bandwidth** | ${\hat{\boldsymbol{\theta}}}_{\boldsymbol{a,a’}}\left( \boldsymbol{a} \right)$ | | | | ${\hat{\boldsymbol{\theta}}}_{\boldsymbol{a,a’}}\left( \boldsymbol{a’} \right)$ | | | | ${\hat{\boldsymbol{\delta}}}_{\boldsymbol{a,a’}}\left( \boldsymbol{a} \right)$ | | | | ${\hat{\boldsymbol{\delta}}}_{\boldsymbol{a,a’}}\left( \boldsymbol{a’} \right)$ | | | |
| --- | --- | --- | --- | --- | --- | --- | --- | --- | --- | --- | --- | --- | --- | --- | --- | --- | --- |
|  |  | **Bias (%)** | **SD** | **RMSE** | **CP (%)** | **Bias (%)** | **SD** | **RMSE** | **CP (%)** | **Bias (%)** | **SD** | **RMSE** | **CP (%)** | **Bias (%)** | **SD** | **RMSE** | **CP (%)** |
| **Scenario 1 (SoSt)** | *h_wp_* | 6.09 | 0.047 | 0.061 | 90.2 | 6.21 | 0.047 | 0.060 | 90.2 | 7.88 | 0.010 | 0.012 | 93.0 | 6.52 | 0.010 | 0.012 | 92.5 |
|  | *h_wp us_* | 1.92 | 0.087 | 0.093 | 94.9 | 2.05 | 0.086 | 0.093 | 94.9 | 2.63 | 0.013 | 0.015 | 94.1 | 4.37 | 0.013 | 0.014 | 92.5 |
| **Scenario 2 (SoWt)** | *h_wp_* | 5.47 | 0.046 | 0.058 | 91.9 | 5.56 | 0.046 | 0.058 | 91.9 | 10.53 | 0.009 | 0.012 | 90.6 | 9.52 | 0.009 | 0.011 | 91.5 |
|  | *h_wp us_* | 1.75 | 0.084 | 0.088 | 95.5 | 1.95 | 0.084 | 0.088 | 95.6 | 1.90 | 0.013 | 0.014 | 94.1 | 3.78 | 0.012 | 0.014 | 93.5 |
| **Scenario 3 (WoSt)** | *h_wp_* | 5.97 | 0.045 | 0.058 | 90.6 | 6.06 | 0.044 | 0.058 | 90.0 | 7.04 | 0.010 | 0.012 | 91.5 | 5.97 | 0.009 | 0.012 | 91.6 |
|  | *h_wp us_* | 1.89 | 0.082 | 0.087 | 94.7 | 2.05 | 0.081 | 0.088 | 94.1 | 2.66 | 0.012 | 0.014 | 93.7 | 5.42 | 0.012 | 0.015 | 92.4 |

**Notes:** $\hat{\theta}_{a,a’}\left( a \right)$, $\hat{\theta}_{a,a’}\left( a’ \right)$ separately represent direct effects under treatment and non-treatment, and $\hat{\delta}_{a,a’}\left( a \right)$, $\hat{\delta}_{a,a’}\left( a’ \right)$ separately represent indirect effects under treatment and non-treatment. “Bias (%)”, “SD”, “RMSE”, and “CP” respectively report the average relative bias, standard deviation, root mean squared error, and coverage probability of the effects across all treatment values *a* ∈ {-1, -0.9, ..., -0.1} ∪ {0.1, ..., 0.9, 1} and *a’*=0. *h_wp_* represents the kernel bandwidth *h* = *C ·n*^-0.25^ and *h_wp us_* represents the undersmoothing bandwidth *h* = (*C ·n*^-0.25^)/2. The analysis is performed under sample size and covariate dimension (*n*, *p*) of (2000, 100) with covariate correlation of 0.

**Abbreviations**: GOAL, generalized outcome-adaptive LASSO; SoSt, the scenario with both strong outcome and treatment; SoWt, the scenario with strong outcome and weak treatment; WoSt, the scenario with weak outcome and strong treatment.

## Table K. Simulation performance of the GOAL-based estimation under binary outcome settings.

| **Scenarios** | **Bandwidth** | ${\hat{\boldsymbol{\theta}}}_{\boldsymbol{a,a’}}\left( \boldsymbol{a} \right)$ | | | | ${\hat{\boldsymbol{\theta}}}_{\boldsymbol{a,a’}}\left( \boldsymbol{a’} \right)$ | | | | ${\hat{\boldsymbol{\delta}}}_{\boldsymbol{a,a’}}\left( \boldsymbol{a} \right)$ | | | | ${\hat{\boldsymbol{\delta}}}_{\boldsymbol{a,a’}}\left( \boldsymbol{a’} \right)$ | | | |
| --- | --- | --- | --- | --- | --- | --- | --- | --- | --- | --- | --- | --- | --- | --- | --- | --- | --- |
|  |  | **Bias (%)** | **SD** | **RMSE** | **CP (%)** | **Bias (%)** | **SD** | **RMSE** | **CP (%)** | **Bias (%)** | **SD** | **RMSE** | **CP (%)** | **Bias (%)** | **SD** | **RMSE** | **CP (%)** |
| **Scenario 1 (SoSt)** | *h_wp_* | 13.23 | 0.017 | 0.025 | 80.3 | 13.15 | 0.018 | 0.025 | 80.8 | 2.95 | 0.003 | 0.003 | 96.2 | 2.55 | 0.003 | 0.003 | 97.5 |
|  | *h_wp_us_* | 6.72 | 0.033 | 0.037 | 91.1 | 6.49 | 0.034 | 0.037 | 92.0 | 3.02 | 0.004 | 0.004 | 95.3 | 1.04 | 0.004 | 0.004 | 95.4 |
| **Scenario 2 (SoWt)** | *h_wp_* | 11.51 | 0.017 | 0.024 | 85.0 | 11.46 | 0.017 | 0.024 | 84.8 | 5.04 | 0.003 | 0.003 | 95.7 | 4.23 | 0.003 | 0.003 | 98.8 |
|  | *h_wp us_* | 4.79 | 0.033 | 0.037 | 92.6 | 4.63 | 0.033 | 0.037 | 92.8 | 2.27 | 0.004 | 0.004 | 96.0 | 1.26 | 0.004 | 0.004 | 98.5 |
| **Scenario 3 (WoSt)** | *h_wp_* | 11.86 | 0.017 | 0.025 | 85.5 | 11.82 | 0.018 | 0.025 | 85.0 | 5.00 | 0.003 | 0.003 | 95.0 | 4.07 | 0.003 | 0.003 | 95.9 |
|  | *h_wp us_* | 4.72 | 0.033 | 0.037 | 92.2 | 4.54 | 0.034 | 0.037 | 92.2 | 2.50 | 0.004 | 0.004 | 96.0 | 2.14 | 0.004 | 0.004 | 94.9 |

**Notes:** $\hat{\theta}_{a,a’}\left( a \right)$, $\hat{\theta}_{a,a’}\left( a’ \right)$ separately represent direct effects under treatment and non-treatment, and $\hat{\delta}_{a,a’}\left( a \right)$, $\hat{\delta}_{a,a’}\left( a’ \right)$ separately represent indirect effects under treatment and non-treatment. “Bias (%)”, “SD”, “RMSE”, and “CP” respectively report the average relative bias, standard deviation, root mean squared error, and coverage probability of the effects across all treatment values *a* ∈ {-1, -0.9, ..., -0.1} ∪ {0.1, ..., 0.9, 1} and *a’*=0. *h_wp_* represents the kernel bandwidth *h* = *C ·n*^-0.25^ and *h_wp us_* represents the undersmoothing bandwidth *h* = (*C ·n*^-0.25^)/2. The analysis is performed under sample size and covariate dimension (*n*, *p*) of (2000, 100) with covariate correlation of 0.

**Abbreviations**: GOAL, generalized outcome-adaptive LASSO; SoSt, the scenario with both strong outcome and treatment; SoWt, the scenario with strong outcome and weak treatment; WoSt, the scenario with weak outcome and strong treatment.

## Table L. Scoring of the Finnish Diabetes Risk Score (FINDRISC).

| **Points** | **Age (years)** | **Family history of diabetes** | **Daily fruit/veg.** | **Physical activity** | **Blood Pressure medication** | **History of High blood glucose** | **BMI (kg/m²)** | **Waist circumference (cm)** | |
| --- | --- | --- | --- | --- | --- | --- | --- | --- | --- |
|  |  |  |  |  |  |  |  | **Women** | **Men** |
| **0** | <45 | No | Yes | Yes | No | No | ≤25 | <80 | <94 |
| **1** |  |  | No |  |  |  | 25-30 |  |  |
| **2** | 45-54 |  |  | No | Yes |  |  |  |  |
| **3** | 55-64 |  |  |  |  |  | >30 | 80-88 | 94-102 |
| **4** | ≥65 |  |  |  |  |  |  | ≥88 | ≥102 |
| **5** |  | Yes |  |  |  | Yes |  |  |  |

**Abbreviations:** BMI, body mass index.

**Notes:** The history of hyperglycemia is defined by whether an individual has been told by a health-care professional that he/she have diabetes or latent diabetes, which covers a history of transient or borderline elevated blood glucose, or gestational diabetes. The total FINDRISC ranges from 0 to 25, with a higher score representing a higher risk of diabetes.

## Table M. Data source for the definition of overall cancer in the UK Biobank.

| **Sources** | **Related field id** | **Specific code** |
| --- | --- | --- |
| ICD-9 | 40001, 40002, 40013, 41271 | 140-208 (except 173) |
| ICD-10 | 41270, 40006 | C00-C97 (except C44) |
| Self-reported | 20001 | 1001-1088 (except 1060-1062, 1073) |

**Abbreviations:** ICD, International Classification of Diseases.

## Table N. Descriptive information of variables used in the real data application.

| **Variable** | **Type** | **Mean** | **SD** | **Min** | **Max** |
| --- | --- | --- | --- | --- | --- |
| Sex | dummy (1, 0) | 0.39 | 0.49 | 0 | 1 |
| Part of a multiple birth | dummy (1, 0) | 0.02 | 0.15 | 0 | 1 |
| Qualifications | categorical | 1.91 | 1.40 | 0 | 6 |
| Townsend deprivation index at recruitment | numeric | -1.93 | 2.66 | -6.26 | 8.94 |
| Income | categorical | 2.20 | 0.82 | 1 | 3 |
| Occupation | dummy (1, 0) | 0.95 | 0.22 | 0 | 1 |
| Water intake | numeric | 2.84 | 2.23 | 0 | 25 |
| Alcohol intake frequency | categorical | 2.57 | 1.34 | 1 | 6 |
| Smoking status | categorical | 0.45 | 0.61 | 0 | 2 |
| Time spent watching television | numeric | 2.15 | 1.33 | 0 | 13 |
| Time spent using computer | numeric | 1.24 | 1.37 | 0 | 15 |
| Time spent driving | numeric | 0.91 | 0.92 | 0 | 14 |
| Length of mobile phone use | categorical | 2.89 | 1.26 | 0 | 4 |
| Sleep duration | numeric | 7.20 | 0.87 | 3 | 14 |
| Tea intake | numeric | 3.29 | 2.60 | 0 | 50 |
| Coffee intake | numeric | 1.95 | 1.92 | 0 | 30 |
| Breastfed as a baby | dummy (1, 0) | 0.75 | 0.44 | 0 | 1 |
| Maternal smoking around birth | dummy (1, 0) | 0.26 | 0.44 | 0 | 1 |
| Use of sun/UV protection | categorical | 2.78 | 0.84 | 1 | 5 |
| Standard PRS for age-related macular degeneration | numeric | 0.11 | 1.02 | -3.51 | 3.39 |
| Standard PRS for Alzheimer's disease | numeric | 0.01 | 0.98 | -2.76 | 4.14 |
| Standard PRS for COPD | numeric | 0.19 | 0.97 | -3.35 | 3.78 |
| Standard PRS for atrial fibrillation | numeric | 0.10 | 0.93 | -2.96 | 3.86 |
| Standard PRS for bipolar disorder | numeric | -0.24 | 1.03 | -4.04 | 4.10 |
| Standard PRS for bowel cancer | numeric | 0.20 | 1.06 | -3.85 | 4.26 |
| Standard PRS for breast cancer | numeric | -0.18 | 1.00 | -4.17 | 3.28 |
| Standard PRS for cardiovascular disease | numeric | -0.20 | 0.98 | -3.98 | 3.30 |
| Standard PRS for coeliac disease | numeric | 0.02 | 1.20 | -2.78 | 5.96 |
| Standard PRS for coronary artery disease | numeric | -0.24 | 0.97 | -3.58 | 3.16 |
| Standard PRS for Crohn's disease | numeric | -0.12 | 0.90 | -4.35 | 3.04 |
| Standard PRS for estimated bone mineral density t-score | numeric | -0.02 | 0.98 | -3.97 | 3.33 |
| Standard PRS for glycated haemoglobin | numeric | 0.05 | 1.04 | -3.83 | 3.65 |
| Standard PRS for height | numeric | 1.07 | 0.71 | -2.54 | 3.40 |
| Standard PRS for high density lipoprotein cholesterol | numeric | 0.04 | 1.00 | -4.55 | 3.81 |
| Standard PRS for hypertension | numeric | -0.11 | 0.96 | -3.35 | 3.64 |
| Standard PRS for intraocular pressure | numeric | -0.03 | 1.01 | -3.63 | 4.36 |
| Standard PRS for ischaemic stroke | numeric | -0.11 | 0.94 | -4.07 | 3.61 |
| Standard PRS for low density lipoprotein cholesterol | numeric | -0.15 | 0.99 | -4.43 | 3.20 |
| Standard PRS for melanoma | numeric | 0.49 | 1.04 | -3.00 | 5.16 |
| Standard PRS for multiple sclerosis | numeric | -0.06 | 1.02 | -3.49 | 4.58 |
| Standard PRS for osteoporosis | numeric | -0.05 | 0.93 | -3.37 | 3.24 |
| Standard PRS for Parkinson's disease | numeric | -0.13 | 1.03 | -4.29 | 3.92 |
| Standard PRS for primary open angle glaucoma | numeric | 0.04 | 1.01 | -4.21 | 3.62 |
| Standard PRS for psoriasis | numeric | -0.25 | 0.99 | -2.93 | 4.96 |
| Standard PRS for rheumatoid arthritis | numeric | 0.12 | 0.98 | -3.13 | 4.88 |
| Standard PRS for schizophrenia | numeric | -0.42 | 0.99 | -3.97 | 3.44 |
| Standard PRS for systemic lupus erythematosus | numeric | 0.11 | 0.97 | -3.32 | 3.76 |
| Standard PRS for type 1 diabetes | numeric | 0.07 | 1.10 | -3.05 | 4.98 |
| Standard PRS for type 2 diabetes | numeric | -0.21 | 0.96 | -3.86 | 3.34 |
| Standard PRS for ulcerative colitis | numeric | 0.10 | 0.99 | -3.56 | 3.67 |
| Standard PRS for venous thromboembolic disease | numeric | 0.02 | 1.02 | -3.31 | 5.44 |
| Diastolic blood pressure | numeric | 80.50 | 10.39 | 41 | 137 |
| Systolic blood pressure | numeric | 134.49 | 18.32 | 78 | 227 |
| Birth weight | numeric | 3.37 | 0.57 | 0.68 | 6.35 |
| Number of treatments/medications taken | numeric | 1.64 | 1.95 | 0 | 14 |
| Hip circumference | numeric | 101.45 | 7.97 | 79 | 150 |
| Felt loved as a child | dummy (1, 0) | 3.44 | 0.90 | 0 | 4 |
| Physically abused by family as a child | dummy (1, 0) | 0.27 | 0.65 | 0 | 4 |
| Felt hated by family member as a child | dummy (1, 0) | 0.28 | 0.75 | 0 | 4 |
| Sexually molested as a child | dummy (1, 0) | 0.13 | 0.50 | 0 | 4 |
| Someone to take to doctor when needed as a child | dummy (1, 0) | 3.84 | 0.59 | 0 | 4 |
| Stopped from seeing friends or family by partner or ex-partner as an adult | dummy (1, 0) | 0.08 | 0.29 | 0 | 2 |
| Belittlement by partner or ex-partner as an adult | categorical | 0.14 | 0.39 | 0 | 2 |
| Physical violence by partner or ex-partner as an adult | categorical | 0.11 | 0.32 | 0 | 2 |
| Sexual interference by partner or ex-partner without consent as an adult | categorical | 0.05 | 0.23 | 0 | 2 |
| Sexual intercourse by partner or ex-partner without consent as an adult | categorical | 0.05 | 0.23 | 0 | 2 |
| Experienced a violent or sexual assault | dummy (1, 0) | 0.08 | 0.28 | 0 | 2 |
| Experienced a marital separation/divorce | dummy (1, 0) | 0.33 | 0.48 | 0 | 2 |
| Experienced the death of a spouse or partner | dummy (1, 0) | 0.13 | 0.36 | 0 | 2 |
| Frequency of seeing friends and family in person | categorical | 3.79 | 1.08 | 0 | 5 |
| Frequency of feeling that lacks companionship | categorical | 0.34 | 0.57 | 0 | 2 |
| Tendency to bounce back quickly after hard times | categorical | 2.76 | 0.95 | 0 | 4 |
| Quick recovery from stressful events | categorical | 2.59 | 0.92 | 0 | 4 |
| Hard to snap back when something bad happens | categorical | 1.29 | 0.94 | 0 | 4 |
| Creatinine (enzymatic) in urine | numeric | 7992.20 | 5410.80 | 613 | 40546 |
| Potassium in urine | numeric | 61.51 | 34.05 | 4.20 | 198.40 |
| Sodium in urine | numeric | 67.34 | 39.81 | 10.00 | 248.00 |
| Alkaline phosphatase | numeric | 76.88 | 22.68 | 17.90 | 476.90 |
| Alanine aminotransferase | numeric | 21.06 | 11.66 | 3.89 | 191.44 |
| Apolipoprotein A | numeric | 1.59 | 0.27 | 0.68 | 2.50 |
| Aspartate aminotransferase | numeric | 24.91 | 9.17 | 10.10 | 389.10 |
| Direct bilirubin | numeric | 1.85 | 0.83 | 1.00 | 9.14 |
| Urea | numeric | 5.17 | 1.18 | 1.63 | 12.81 |
| Calcium | numeric | 2.38 | 0.09 | 1.49 | 2.99 |
| Cholesterol | numeric | 5.59 | 0.93 | 2.21 | 7.90 |
| Creatinine | numeric | 70.90 | 13.08 | 34.40 | 192.20 |
| C-reactive protein | numeric | 1.73 | 3.00 | 0.08 | 77.64 |
| Cystatin C | numeric | 0.85 | 0.13 | 0.42 | 4.14 |
| Gamma glutamyltransferase | numeric | 30.32 | 30.76 | 6.70 | 1160.80 |
| Glycated haemoglobin (HbA1c) | numeric | 34.25 | 4.56 | 15.60 | 97.00 |
| High density lipoprotein cholesterol | numeric | 1.54 | 0.37 | 0.45 | 3.22 |
| Insulin-Like Growth Factor-1 | numeric | 22.63 | 5.49 | 5.23 | 60.89 |
| Low density lipoprotein direct | numeric | 3.45 | 0.72 | 1.19 | 5.78 |
| Lipoprotein A | numeric | 43.59 | 49.25 | 3.80 | 189.00 |
| Total protein | numeric | 72.17 | 3.87 | 55.73 | 94.33 |
| Triglycerides | numeric | 1.44 | 0.82 | 0.32 | 8.34 |
| White blood cell (leukocyte) count | numeric | 6.50 | 1.66 | 1.62 | 28.87 |
| Neutrophil-lymphocyte ratio | numeric | 2.31 | 1.12 | 0.04 | 35.64 |

**Abbreviations:** SD, standard deviation; PRS, polygenic risk score.

**Notes:** The description of the coding information for some of the covariates is presented in Table S8.

## Table O. Details of variable coding in the real data application.

| **Variable** | **Coding description** |
| --- | --- |
| sex | 1, male; 0, female |
| qualification | 6, other professional qualifications, e.g., nursing, teaching; 5, NVQ or HND or HNC or equivalent; 4, CSEs or equivalent (CSEs, National Vocational Qualifications); 3, ordinary level (O-levels) or general certificate of secondary education (GCSEs); 2, A levels/AS levels or equivalent; 1, college or University degree; 0, none of the above |
| Townsend deprivation index at recruitment | Townsend deprivation index is calculated immediately prior to participant joining UK Biobank based on the preceding national census output areas. Each participant is assigned a score corresponding to the output area in which their postcode is located. A positive value indicates worse socioeconomic status (SES), a negative one indicates better SES, and zero representing an average state. |
| occupation | 1, employed (in paid employment or self-employed, retired, doing unpaid or voluntary work, and being full or part-time students); 0, unemployed (looking after home / family, unable to work because of sickness or disability, and unemployed) |
| income | 3, greater than 52,000 £; 2, 31,000 to 51,999 £; 1, less than 18,000 £ |
| length of mobile phone use | 4, more than eight years; 3, five to eight years; 2, two to four years; 1, one year or less; 0, never used mobile phone at least once per week |
| alcohol intake frequency | 6, never; 5, special occasions only; 4, one to three times a month; 3, once or twice a week; 2, three or four times a week; 1, daily or almost daily |
| smoking status | 2, current; 1, previous; 0, never |
| use of sun/UV protection | 5, do not go out in sunshine; 4, always; 3, most of the time; 2, sometimes; 1, never / rarely |
| felt loved as a child / physically abused by family as a child / felt hated by family members as a child / sexually molested as a child / someone to take to the doctor when needed as a child | 4, very often true; 3, often true; 2, sometimes true; 1, rarely true; 0, never true |
| stopped from seeing friends or family by partner or ex-partner as an adult / belittlement by partner or ex-partner as an adult / physical violence by partner or ex-partner as an adult / sexual interference by partner or ex-partner without consent as an adult / sexual intercourse by partner or ex-partner without consent as an adult / experienced a violent or sexual assault / experienced a marital separation/divorce / experienced the death of a spouse or partner | 2, yes, within the last 12 months; 1, yes, but not in the last 12 months; 0, no, never |
| frequency of seeing friends and family in person | 5, daily or almost daily; 4, 2-4 times a week; 3, about once a week; 2, about once a month; 1, once every few months; 0, never or almost never |
| frequency of feeling that lacks companionship | 2, often; 1, some of the time; 0, hardly ever |
| tendency to bounce back quickly after hard times / quick recovery from stressful events / hard to snap back when something bad happens | 4, strongly agree; 3, agree; 2, neutral; 1, disagree; 0, strongly disagree |
| neutrophil-lymphocyte ratio | Neutrophil-lymphocyte ratio is calculated by dividing the neutrophil count by the lymphocyte count. |

## Table P. Bandwidth diagnostic results in the real-data application

| **Bandwidth** | **Eff. window half-width** | **ESS (Weight)** | **ESS (Kernel)** | **ESS ratio** | **ESS-weighted mean SMD** | **SMD** | **CV (active)** |
| --- | --- | --- | --- | --- | --- | --- | --- |
| *h_wp_* | 0.57 **(<1)** | - | - | - | - | - | - |
| *h_wp_* _us_ | 0.31 **(<1)** | - | - | - | - | - | - |
| 0.75 *h_ROT_* | 2.61 | 134.6 | 141.8 | 0.73 | 0.15 | 0.31 | 0.73 |
| *h_ROT_* | 3.48 | 249.1 | 258.8 | 0.73 | 0.10 | 0.24 | 0.73 |
| 1.25 *h_ROT_* | 4.35 | 346.6 | 360.6 | 0.76 | 0.10 | 0.17 | 0.79 |
| 1.5 *h_ROT_* | 5.22 | 427.6 | 446.1 | 0.83 | 0.11 | 0.20 | 0.80 |
| 2 *h_ROT_* | 6.96 | 579.2 | 605.6 | 0.82 | 0.15 | 0.26 | 0.62 |

**Note:** The *h_wp_* corresponds to the kernel bandwidth that is set to *C*·*n*^-0.25^ with *C*=2.34 in the standard weighted semiparametric estimator. The *h_wp_* _us_ corresponds to the undersmoothing version of the *h_wp_* kernel bandwidth, specified by halving it as *C*·*n*^-0.25^/2. *h_ROT_* corresponds to the rule-of-thumb (ROT) kernel bandwidth as *C* · sd(*A*)·*n*^-1/5^ with *C* =2.34. Eff. window half-width represents the effective kernel window half-width, which is defined as *h*·$\sqrt{5}$, where $\sqrt{5}$ reflects the relationship between the bandwidth parameter and the support half-width of the second-order Epanechnikov kernel. The minimum values of ESS (weight), ESS (kernel), and the ESS ratio, and the maximum values of SMD-related metrics and CV across the exposure range are reported. In the empirical application settings, bandwidth *h_wp_* and *h_wp us_* are considered structurally invalid because their effective window half-widths are both smaller than the minimum spacing of the exposure variable, e.g., 1 unit for the integer-valued exposure score.

**Abbreviations**: ESS, effective sample size; SMD, standardized mean difference; CV, coefficient of variation.

## Table Q. Bootstrap-based average standard deviations for the GOAL-based estimation.

| **Data sample** | **Average Standard Deviation** | | | |
| --- | --- | --- | --- | --- |
|  | **NDE (treatment)** | **NDE (non-treatment)** | **NIE (treatment)** | **NIE (non-treatment)** |
| original empirical data | 0.013 | 0.013 | 0.013 | 2.33E-03 |
| bootstrap samples | 0.015 | 0.016 | 0.015 | 2.34E-03 |

**Note**: The average standard deviations are calculated by averaging the standard deviation of the effects across the treatment values *a* ∈ {3, 4, ..., 13} versus *a’*=2. NDE (treatment or non-treatment) (same as that for NIE) separately indicates the natural direct effect estimated under *A=a* or the natural direct effect estimated under *A=a’*. The average standard deviations for bootstrap samples are calculated across 100 bootstrap resamples from the original empirical data.

**Abbreviations**: GOAL, general outcome-adaptive LASSO; NDE, natural direct effect; NIE, natural indirect effect.

## Table R. Sensitivity analysis using varying bandwidths in the real data application.

| **Scenarios and Methods** | **Average Standard Deviation** | | | |
| --- | --- | --- | --- | --- |
|  | **NDE  (treatment)** | **NDE  (non-treatment)** | **NIE  (treatment)** | **NIE  (non-treatment)** |
| Bandwidth with *C*=1.7 |  |  |  |  |
| GOAL | 0.018 | 0.017 | 2.73E-03 | 2.23E-03 |
| AdaLASSO | 0.023 | 0.022 | 3.19E-03 | 2.58E-03 |
| LASSO | 0.023 | 0.023 | 3.05E-03 | 2.58E-03 |
| Full | 0.023 | 0.023 | 2.50E-03 | 2.33E-03 |
| Bandwidth with *C*=2.0 |  |  |  |  |
| GOAL | 0.017 | 0.017 | 2.61E-03 | 2.23E-03 |
| AdaLASSO | 0.022 | 0.021 | 3.09E-03 | 2.52E-03 |
| LASSO | 0.023 | 0.023 | 2.98E-03 | 2.57E-03 |
| Full | 0.023 | 0.022 | 2.50E-03 | 2.27E-03 |
| Bandwidth with *C*=2.5 |  |  |  |  |
| GOAL | 0.013 | 0.012 | 2.21E-03 | 1.77E-03 |
| AdaLASSO | 0.016 | 0.015 | 2.58E-03 | 1.95E-03 |
| LASSO | 0.017 | 0.017 | 2.46E-03 | 1.96E-03 |
| Full | 0.018 | 0.018 | 2.14E-03 | 1.81E-03 |

**Note:** The bandwidth settings are set to *C*=1.7, 2.0, and 2.5 for *C* · sd(*A*)·*n*^-1/5^.

**Abbreviations**: LASSO, the least absolute shrinkage and selection operator; GOAL, generalized outcome-adaptive LASSO; AdaLASSO, adaptive LASSO; NDE, natural direct effect; NIE, natural indirect effect.
