# Supplementary material for: Variable selection-combined causal mediation analysis for continuous treatments with application to large-dimensional biomedical data
Source: PLoS Comput Biol. 2026 Jun 24;22(6):e1014436. doi: 10.1371/journal.pcbi.1014436 (PMC13327524; doi:10.1371/journal.pcbi.1014436)
Supplement: S2 Appendix — All supplementary figures in the study, including variable selection results for different sample sizes and model sparsity, effect estimation trajectories under different bandwidth options, and variable selection practices in real-world applications. (DOCX) [file pcbi.1014436.s002.docx]

# S2 Appendix

**Variable selection-combined causal mediation analysis for continuous treatments with application to large-dimensional biomedical data**

Yajing Zhou^1^, Kecheng Wei^1^, Yahang Liu^1^, Zhaoyang Li^1^, Chen Huang^1^, Guoyou Qin^2*^, Yongfu Yu^1,3*^

^1^ Department of Biostatistics, NHC Key Laboratory for Health Technology Assessment, Key Laboratory of Public Health Safety of Ministry of Education, School of Public Health, Fudan University, Shanghai 200032, China

^2^ Shanghai Institute of Infectious Disease and Biosecurity, Fudan University, Shanghai, China

^3^ Shanghai Key Laboratory of Gene Editing and Cell Therapy for Rare Diseases, Fudan University, Shanghai 200031, China

* [yu@fudan.edu.cn](mailto:yu@fudan.edu.cn); [gyqin@fudan.edu.cn](mailto:gyqin@fudan.edu.cn)

**Contents**

**Fig A.** Proportion of the top 30 covariates selected using GOAL, AdaLASSO, and LASSO-based methods in the treatment generalized propensity score model given covariates under Scenarios 1–3 with (*n*, *p*) = (5000, 200).

**Fig B.** Proportion of the top 30 covariates selected using GOAL, AdaLASSO, and LASSO-based methods in the treatment generalized propensity score model given both covariates and the mediator under Scenario 1-3 with (*n*, *p*) = (5000, 200).

**Fig C**. Proportion of the top 30 covariates selected by the GOAL-based method in the treatment GPS models given covariates and given both covariates and mediator under different model sparsity.

**Fig D.** Direct effects ${\hat{\boldsymbol{\theta}}}_{\boldsymbol{a}\mathbf{,2}}\left( \boldsymbol{a} \right)$ and ${\hat{\boldsymbol{\theta}}}_{\boldsymbol{a}\mathbf{,2}}\left( \mathbf{2} \right)$ estimation using the GOAL-based method under diagnostic optimal bandwidths.

**Fig E.** Indirect effects ${\hat{\boldsymbol{\delta}}}_{\boldsymbol{a}\mathbf{,2}}\left( \boldsymbol{a} \right)$ and ${\hat{\boldsymbol{\delta}}}_{\boldsymbol{a}\mathbf{,2}}\left( \mathbf{2} \right)$ estimation using the GOAL-based method under diagnostic optimal bandwidths.

**Fig F.** The variability plot of variable selection results for the generalized propensity score model given covariates based on the GOAL-based method.

**Fig G.** The variability plot of variable selection results for the generalized propensity score model given covariates and the mediator based on the GOAL-based method.

**Fig H.** Direct effects $\hat{\theta}_{a,2}\left( a \right)$ and $\hat{\theta}_{a,2}\left( 2 \right)$ estimation using the GOAL-based method under different bandwidth choices.

**Fig I.** Indirect effects $\hat{\delta}_{a,2}\left( a \right)$ and $\hat{\delta}_{a,2}\left( 2 \right)$ estimation using the GOAL-based method under different bandwidth choices.

**Fig J**. Density histogram of Apolipoprotein B (mediator).

## Fig A. Proportion of the top 30 covariates selected using GOAL, AdaLASSO, and LASSO-based methods in the treatment generalized propensity score model given covariates under Scenarios 1–3 with (*n*, *p*) = (5000, 200).

**Notes:** The horizontal axis represents the index of the simulated covariates, and the vertical axis denotes the proportion of covariates repeatedly selected across 100 simulations.

**Abbreviations**: LASSO, the least absolute shrinkage and selection operator; GOAL, generalized outcome-adaptive LASSO; AdaLASSO, adaptive LASSO.

## Fig B. Proportion of the top 30 covariates selected using GOAL, AdaLASSO, and LASSO-based methods in the treatment generalized propensity score model given both covariates and the mediator under Scenario 1-3 with (*n*, *p*) = (5000, 200).

**Notes:** The horizontal axis represents the index of the simulated covariates, and the vertical axis denotes the proportion of covariates repeatedly selected across 100 simulations.

**Abbreviations**: LASSO, the least absolute shrinkage and selection operator; GOAL, generalized outcome-adaptive LASSO; AdaLASSO, adaptive LASSO.

## Fig C. Proportion of the top 30 covariates selected by the GOAL-based method in the treatment GPS models given covariates and given both covariates and mediator under different model sparsity.

**Notes**: The horizontal axis represents the index of the simulated covariates, and the vertical axis denotes the proportion of covariates repeatedly selected across 100 simulations. The sensitivity analysis is conducted using the simulation data of (*n*, *p*) = (2000, 100) with covariate correlation of 0 under Scenario 1. “s” indicates the variable numbers of confounders or prognostic variables, with a smaller number indicating a strong sparsity of the model.

**Abbreviations**: GOAL, generalized outcome-adaptive LASSO; GPS, generalized propensity score.

## Fig D. Direct effects ${\hat{\boldsymbol{\theta}}}_{\boldsymbol{a}\mathbf{,2}}\left( \boldsymbol{a} \right)$ and ${\hat{\boldsymbol{\theta}}}_{\boldsymbol{a}\mathbf{,2}}\left( \mathbf{2} \right)$ estimation using the GOAL-based method under diagnostic optimal bandwidths.

**Notes**: The treatment value *a* ∈ {3, 4, ..., 13}**.** The comparison bandwidths involve the rule-of-thumb bandwidth (*h_ROT_*), which is defined as *C*·sd(*A*)·*n*^-1/5^, and its multiples, 0.75*h_ROT_* and 1.25*h_ROT_*. The horizontal axis represents the continuous treatment value in increments of 2, and the vertical axis denotes the magnitude of the natural direct effect (NDE) under treatment or non-treatment. The solid black line with hollow dots depicts the point estimates of the NDE at varying treatment levels, and the grey dashed lines surrounding the solid line represent the 95% pointwise confidence intervals derived from 500 bootstrap resampling. The horizontal dashed line at zero represents no causal effect observed on the risk difference scale through the direct pathway between the treatment and the outcome. Estimated values above zero suggest a risk effect, while values below zero indicate a protective effect.

**Abbreviations**: GOAL, generalized outcome-adaptive LASSO; FINDRISC, Finnish Diabetes Risk Score.

## Fig E. Indirect effects ${\hat{\boldsymbol{\delta}}}_{\boldsymbol{a}\mathbf{,2}}\left( \boldsymbol{a} \right)$ and ${\hat{\boldsymbol{\delta}}}_{\boldsymbol{a}\mathbf{,2}}\left( \mathbf{2} \right)$ estimation using the GOAL-based method under diagnostic optimal bandwidths.

**Notes**: The treatment value *a* ∈ {3, 4, ..., 13}**.** The comparison bandwidths involve the rule-of-thumb bandwidth (*h_ROT_*), which is defined as *C*·sd(*A*)·*n*^-1/5^, and its multiples, 0.75*h_ROT_* and 1.25*h_ROT_*. The horizontal axis represents the continuous treatment value in increments of 2, and the vertical axis denotes the magnitude of the natural indirect effect (NIE) under treatment or non-treatment. The solid black line with hollow dots depicts the point estimates of the NIE at varying treatment levels, and the grey dashed lines surrounding the solid line represent the 95% pointwise confidence intervals derived from 500 bootstrap resampling. The horizontal dashed line at zero represents no causal effect observed on the risk difference scale through the indirect pathway between the treatment and the outcome. Estimated values above zero suggest a risk effect, while values below zero indicate a protective effect.

**Abbreviations**: GOAL, generalized outcome-adaptive LASSO; FINDRISC, Finnish Diabetes Risk Score.

## Fig F. The variability plot of variable selection results for the generalized propensity score model given covariates based on the GOAL-based method.


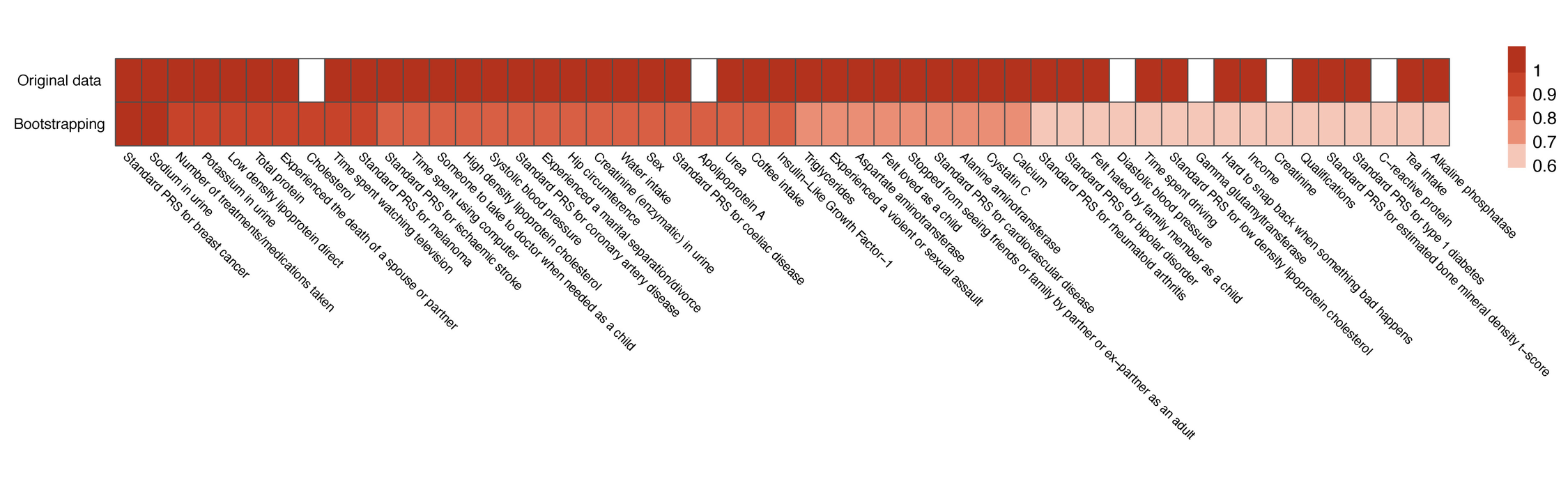


**Notes**: The variables listed in the heatmap are calculated with a selection frequency of no less than 60% using the GOAL-based method from 100 bootstrap resampling based on the original empirical data, sorted in descending order in terms of selection proportion. The legend on the right uses varying shades of red to indicate the different selection proportions ranging from 0.6 to 1. The colors corresponding to the ‘Original data’ row only represent whether the GOAL-based method selects the variable in our single trial of real data application (red indicates selected), which does not reflect the frequency.

**Abbreviations**: GOAL, generalized outcome-adaptive LASSO.

## Fig G. The variability plot of variable selection results for the generalized propensity score model given covariates and the mediator based on the GOAL-based method.


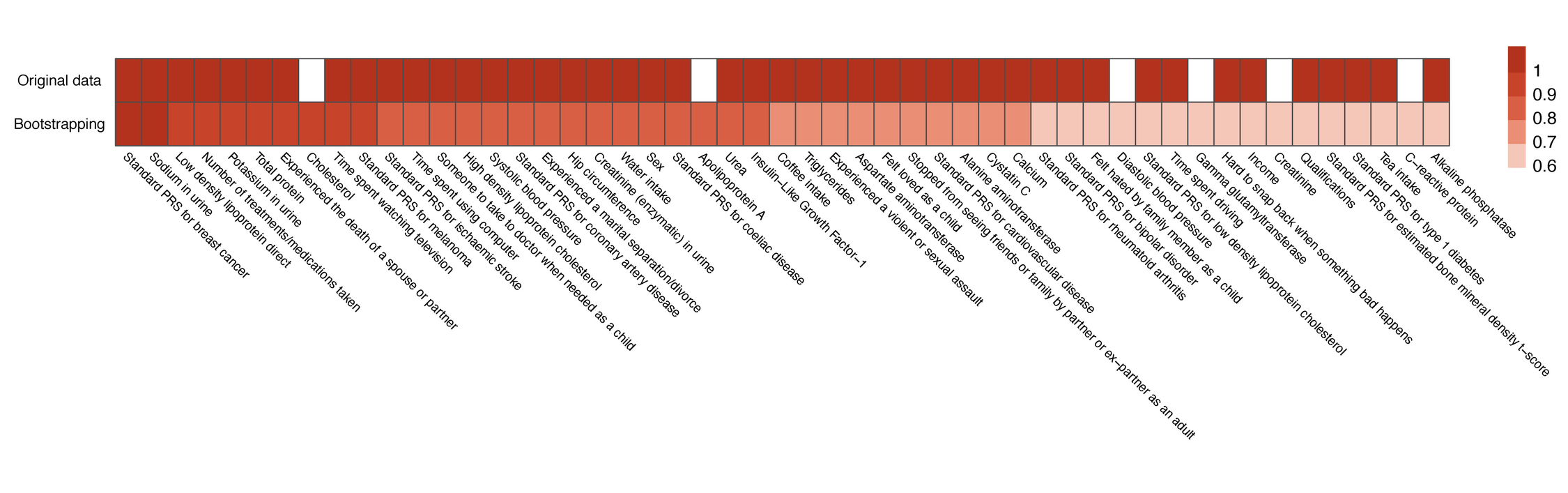


**Notes**: The variables listed in the heatmap are calculated with a selection frequency of no less than 60% using the GOAL-based method from 100 bootstrap resampling based on the original empirical data, sorted in descending order in terms of selection proportion. The legend on the right uses varying shades of red to indicate the different selection proportions ranging from 0.6 to 1. The colors corresponding to the ‘Original data’ row only represent whether the GOAL-based method selects the variable in our single trial of real data application (red indicates selected), which does not reflect the frequency.

**Abbreviations**: GOAL, generalized outcome-adaptive LASSO.

## Fig H. Direct effects ${\hat{\boldsymbol{\theta}}}_{\boldsymbol{a}\mathbf{,2}}\left( \boldsymbol{a} \right)$ and ${\hat{\boldsymbol{\theta}}}_{\boldsymbol{a}\mathbf{,2}}\left( \mathbf{2} \right)$ estimation using the GOAL-based method under different bandwidth choices.

**Notes**: The treatment value *a* ∈ {3, 4, ..., 13}**.** The rule-of-thumb bandwidth is *C*·sd(*A*)·*n* ^-1/5^, where *C* is 2.34. Other bandwidth settings are set to C=1.7, 2.0, and 2.5 for *C*·sd(*A*)·*n* ^-1/5^. The horizontal axis represents the continuous treatment value in increments of 2, and the vertical axis denotes the magnitude of the natural direct effect (NDE) under treatment or non-treatment. The solid black line with hollow dots depicts the point estimates of the NDE at varying treatment levels, and the grey dashed lines surrounding the solid line represent the 95% pointwise confidence intervals derived from 500 bootstrap resampling. The horizontal dashed line at zero represents no causal effect observed on the risk difference scale through the direct pathway between the treatment and the outcome. Estimated values above zero suggest a risk effect, while values below zero indicate a protective effect.

**Abbreviations**: GOAL, generalized outcome-adaptive LASSO; FINDRISC, Finnish Diabetes Risk Score.

## Fig I. Indirect effects ${\hat{\boldsymbol{\delta}}}_{\boldsymbol{a}\mathbf{,2}}\left( \boldsymbol{a} \right)$ and ${\hat{\boldsymbol{\delta}}}_{\boldsymbol{a}\mathbf{,2}}\left( \mathbf{2} \right)$ estimation using the GOAL-based method under different bandwidth choices.

**Notes:** The treatment value *a* ∈ {3, 4, ..., 13}**.** The rule-of-thumb bandwidth is *C* · sd(*A*)·*n* ^-1/5^, where *C* is 2.34. Other bandwidth settings are set to C=1.7, 2.0, and 2.5 for *C* · sd(*A*)·*n* ^-1/5^. The horizontal axis represents the continuous treatment value in increments of 2, and the vertical axis denotes the magnitude of the natural indirect effect (NIE) under treatment or non-treatment. The solid black line with hollow dots depicts the point estimates of the NIE at varying treatment levels, and the grey dashed lines surrounding the solid line represent the 95% pointwise confidence intervals derived from 500 bootstrap resampling. The horizontal dashed line at zero represents no causal effect observed on the risk difference scale through the indirect pathway between the treatment and the outcome. Estimated values above zero suggest a risk effect, while values below zero indicate a protective effect.

**Abbreviations**: GOAL, generalized outcome-adaptive LASSO; FINDRISC, Finnish Diabetes Risk Score.

## Fig J. Density histogram of Apolipoprotein B (mediator).

**Notes**: The measurement unit of apolipoprotein B is g/L. The horizontal axis displays the apolipoprotein B levels, in increments of 0.2, and the vertical axis indicates the number of participants within each interval of apolipoprotein B.
