# Supplementary material for: Variable selection-combined causal mediation analysis for continuous treatments with application to large-dimensional biomedical data
Source: PLoS Comput Biol. 2026 Jun 24;22(6):e1014436. doi: 10.1371/journal.pcbi.1014436 (PMC13327524; doi:10.1371/journal.pcbi.1014436)
Supplement: S3 Appendix — All supplementary text in the study, including key assumptions of the method and their validation, simulation setups and algorithms for additional or complex scenarios, and theories and diagnostics related to bandwidth specification. (DOCX) [file pcbi.1014436.s003.docx]

# S3 Appendix

**Variable selection-combined causal mediation analysis for continuous treatments with application to large-dimensional biomedical data**

Yajing Zhou^1^, Kecheng Wei^1^, Yahang Liu^1^, Zhaoyang Li^1^, Chen Huang^1^, Guoyou Qin^2*^, Yongfu Yu^1,3*^

^1^ Department of Biostatistics, NHC Key Laboratory for Health Technology Assessment, Key Laboratory of Public Health Safety of Ministry of Education, School of Public Health, Fudan University, Shanghai 200032, China

^2^ Shanghai Institute of Infectious Disease and Biosecurity, Fudan University, Shanghai, China

^3^ Shanghai Key Laboratory of Gene Editing and Cell Therapy for Rare Diseases, Fudan University, Shanghai 200031, China

* [yu@fudan.edu.cn](mailto:yu@fudan.edu.cn); [gyqin@fudan.edu.cn](mailto:gyqin@fudan.edu.cn)

**Contents**

**Text A.** Assumption 1-3, 5.

**Text B.** Algorithm and Simulation for Binary Outcome Extension.

**Text C.** Theoretical and Diagnostic Bases for Bandwidth Specification in Simulations and Real-Data Applications.

**Text D.** Simulation under Normally Distributed Errors.

**Text E.** Robustness Check of Interaction and Nonlinear Settings.

**Text F.** Sparsity Tests for Different Numbers of True Active Signals.

**Text G.** Sensitivity Analysis under Varying Covariate-to-Sample Size Ratios.

**Text H.** LASSO and Adaptive LASSO estimates.

**Text I.** Assessment of Key Assumptions in the Real Data Analysis.

**Reference**

## Text A. Assumption 1-3, 5

**Assumption 1** (Consistency assumption). *M = M*(*a*) and *Y* = *Y* (*a, M* (*a'*)) under the observed treatment level *a*, *a'* ∈ $\mathcal{A}$.

**Assumption 2** (Positivity assumption).

(i) *f_A_* (*a |* *X* = *x*) > 0 for all (*a, x*) ∈ $\mathcal{A\times X}$.

(ii) *f_A_* (*a |* *M* = *m*, *X* = *x*) > 0 for all (*a, m, x*) ∈ $\mathcal{A\times M\times X}$.

This assumption indicates a common support restriction, where *f_A_* (*a | B = b*) denotes the conditional density of variable *A* taking a specific value *a* given that variable *B* equals b. Also, *f_M_* (m | *A = a, X = x*) > 0 can be sequentially drawn by Bayes' theorem based on Assumption 2.

**Assumption 3** (Conditional independence assumption).

(i) Conditional independence of the treatment. {*Y* (*a′, m*)*, M* (*a*)} ⟂ *A* | *X* = *x* for all (*a, a′, m, x*) ∈ $\mathcal{A}^{2}\mathcal{\times M \times X}$.

(ii) Conditional independence of the mediator. *Y* (*a′, m*) ⟂ *M* (*a*) | *A* = *a, X* = *x* for all (*a, a′, m, x*) ∈ $\mathcal{A}^{2}\mathcal{\times M\times X}$.

Assumption 3, also named the sequential ignorability assumption as described in the literature,[1, 2] excludes unobserved confounders that: i) jointly affect the conditional association of treatment with the mediator, and/or with the outcome given *X*; ii) jointly affect the association between the mediator and the outcome given *A* and *X*.

Compared to the binary treatment scenarios, the positivity and conditional independence assumptions for continuous treatments and mediators must hold for their entire support, which makes the assumptions much stronger.

**Assumption 4** (Parametric generalized propensity scores). $f_{A}\left( a | x \right)$ and $f_{A}\left( a | m,x \right)$ are uniformly bounded above and bounded away from zero on $\mathcal{A\times M\times X}$.

**Assumption 5** (Regularity conditions).

(i) The data {$A_{i}, M_{i}, Y_{i}, X_{i}$}, *i*=1, ..., *n*, are independent and identically distributed.

(ii) The probability density function $f_{AMX}(a,m,x)$ is bounded away from zero and is at least r-order continuously differentiable with respect to (*a*, *m*, *x*), with uniformly bounded derivatives on $\mathcal{A\times M \times X}$, a compact and convex subset of $\mathcal{R}^{1+s_{m}+s_{x}}$, where $s_{m}$ and $s_{x}$ are the dimensions of M and X, respectively.

(iii) $E [Y|A=a, M=m, X=x]$ is at least r-order continuously differentiable with respect to (*a*, *m*, *x*) on $\mathcal{A\times M \times X}$ and has uniformly bounded derivatives.

(iv) The symmetric kernels *k* is bounded differentiable, have convex bounded supports, and have order r ≥ 2. A kernel *k* is of order *r* if $\int k\left( u \right)du=1$, $\int u^{l}k\left( u \right)du=0$ for 0<*l*<*r*, and $\int\left| u^{r}k\left( u \right) \right|du<\infty$.

## Text B. Algorithm and Simulation for Binary Outcome Extension.

**B.1 GOAL algorithm.**

Contrary to continuous outcomes, the unpenalized coefficient $\tilde{\beta}_{j}$ of the jth covariate in the ‘full’, outcome regression model for binary outcomes is calculated by maximum likelihood estimation under a logistic model:

$$\left( \tilde{\alpha},\tilde{\beta}, \tilde{\eta} \right)=arg\min_{\alpha, \beta,\eta} \sum log(1+exp(\alpha A+\eta M+\sum_{j=1}^{p} X_{j}\beta_{j}))-Y(\alpha A+\eta M+\sum_{j=1}^{p} X_{j}\beta_{j}),$$

**B.2 Simulations under binary outcomes.**

To evaluate the performance of the proposed GOAL method for binary outcomes, we conduct a robustness analysis in the simulations with a binary outcome under a sample size of 2000. The outcome generation process is as follows, with other variables remaining unchanged:

$$logit\left\{ P\left( Y_{i}=1 \right) \right\}=\alpha A+\eta M+\sum_{j=1}^{p} \beta_{j}X_{j}$$

where $\alpha=1, \eta=0.3$, and the $\beta$ values remain consistent with the simulation under continuous outcomes, varying across the three scenarios based on the differing strengths of association between covariates and outcomes or treatments.

In the simulation settings under binary outcomes, the true values of causal effects do not have closed- form analytical solutions due to the nonlinear nature of the logistic data-generating process. Therefore, Monte Carlo numerical integration was employed to approximate the true values. Specifically, we generate *N*=100,000 observations of covariates $\boldsymbol{X}$ from the same uniform distribution *U* (-1, 1), and the

expected potential outcomes for given (𝑎, $a’$) were computed as follows:

$$E\left[ Y\left( a,M\left( a^{'} \right) \right) \right]=\frac{1}{N}\sum_{k=1}^{N} \mathrm{logit}^{-1}(\boldsymbol{X}_{k}\boldsymbol{\beta}_{YX}+\alpha_{A}\cdot A+\alpha_{M}\cdot(\boldsymbol{X}_{k}\boldsymbol{\beta}_{MX}+\boldsymbol{\beta}_{AM}\cdot a’))$$

where $\mathrm{logit}^{-1}=e^{(\cdot)}/(1+e^{(\cdot)})$ denotes the sigmoid function, $\boldsymbol{\beta}_{YX}$ and $\boldsymbol{\beta}_{MX}$ are the coefficient vectors representing the effects of covariates on the outcome and the mediator, respectively, and $\alpha_{A}$, $\alpha_{M}$, and $\boldsymbol{\beta}_{AM}$ denote the effects of treatment on outcome, mediator on outcome, and treatment on mediator, respectively. The true values of NDE and NIE are then obtained as differences between the corresponding expected potential outcomes in Equations 2 and 3.

## Text C. Theoretical and Diagnostic Bases for Bandwidth Specification in Simulations and Real-Data Applications.

**C.1 Theoretical basis for bandwidth specification**

**C.1.1 Bandwidth specification in the simulation study**

The proposed estimator uses kernel weighting for continuous exposure and generalized propensity score (GPS) weighting for confounding adjustment. Bandwidth selection therefore, affects both the locality of the exposure-specific estimands and the stochastic variability of the weighted estimator. The bandwidths used in the simulation study are specified following the semiparametric GPS weighting framework of Huber et al. (2020),[3] upon which our method is based, and the general theory of kernel-based semiparametric inference.[3-5]

Specifically, we consider two small-bandwidth specifications: 1) *h_wp_*=*C⋅n*^−0.25^ with *C*=2.34; 2) a further undersmoothing bandwidth *h_wp us_*= *h_wp_*/2. For one-dimensional kernel density or local regression estimation, the conventional MSE-optimal bandwidth is typically of order *n*^−1/5^, reflecting the usual balance between squared bias and variance.[6-9] However, for kernel-based inference, the MSE-optimal bandwidth may leave non-negligible smoothing bias in the studentized statistic. Therefore, smaller bandwidths are commonly considered, especially when conventional bootstrap or Wald-type confidence intervals are used, because undersmoothing can reduce the contribution of smoothing bias to the sampling distribution, albeit at the cost of increased variance. [4, 5, 10]

In the semiparametric case proposed by Huber et al. (2020),[3] asymptotic normality is based on small-bandwidth conditions: $h\to0, nh\to\infty, nh^{5}\to0$, with convergence rate $\sqrt{nh}$ for the kernel-smoothed pointwise estimand. The choice *h*=*C⋅n*^−0.25^ satisfies these conditions because $nh=C\cdot n^{0.75}\to\infty$ and $nh^{5}=C^{5}\cdot n^{-0.25}\to0$. This bandwidth shrinks faster than the conventional *n*^−1/5^ rate and is therefore compatible with the small-bandwidth logic required to make smoothing bias asymptotically negligible relative to stochastic variation in kernel-based inference.

The undersmoothing bandwidth *h_wp us_*= *h_wp_*/2 represents an additional finite-sample undersmoothing specification. Reducing the bandwidth further decreases smoothing bias while increasing sampling variability, which constitutes the classic bias-variance trade-off in kernel smoothing.[7-9] This specification is included to evaluate whether additional bias reduction improves finite-sample confidence interval coverage. Our simulation results also show that the bandwidth *h_wp us_* produces lower estimation bias and better confidence interval coverage compared to *h_wp_*, consistent with this theoretical motivation.

**C.1.2 Bandwidth specification in the real-data application**

The rule-of-thumb (ROT) bandwidth, *h_ROT_*=*C*⋅sd(*A*)⋅*n*^−1/5^ with *C*=2.34, is considered as the empirical reference bandwidth for kernel density estimation.[6] This choice is also consistent with the practical implementation in the prior work of Huber et al. (2020),[3] and with the conventional normal-reference logic for one-dimensional kernel smoothing, where the *n*^−1/5^ rate reflects the MSE-optimal order under standard smoothness assumptions. Its data-adaptive scaling by sd(*A*) accounts for the empirical dispersion of the exposure distribution, making it more appropriate for real-data applications than a fixed bandwidth constant independent of exposure scale.[7]

**C.2 Diagnostic procedure for bandwidth evaluation**

To preliminarily assess the local empirical support, weight stability, and covariate balance of the bandwidth specification, candidate bandwidths are evaluated using a series of diagnostic metrics before reviewing outcome-based estimation performance. The interpretation of these diagnostic metrics can be made directly based on the presence of strict thresholds, or may depend on a comprehensive comparison across different bandwidths. The diagnostic metrics adopted are as follows:

(1) Effective window half-width

To diagnose the local empirical support induced directly by the kernel bandwidth, we calculate the effective kernel window half-width for each candidate bandwidth. For each candidate bandwidth *h*, the effective kernel window half-width is calculated as $H\left( h \right)= h\sqrt{5}$, which describes the degree of local smoothing. This follows from the compact support of the second-order Epanechnikov kernel, under which observations receive a non-zero kernel weight only if $\mid A_{i}-a_{1}\mid<h\sqrt{5}$, where $A_{i}$ denotes the observed exposure and $a_{1}$ denotes the specific evaluation point.[7] For an integer-valued exposure (such as FINDRISC in our empirical analysis), it also provides a structural support diagnostic: if $H\left( h \right)$ is smaller than the minimum spacing between adjacent observed exposure levels, neighboring exposure values cannot contribute to the local estimate.[11] Therefore, this can be used to assess whether a candidate bandwidth provided sufficient local support across the exposure range.[8]

A relevant locality diagnostic is also considered by comparing the full effective window width $2H\left( h \right)$ with the total exposure range. If the full effective window approaches or exceeds the exposure range, the estimator may behave more like a global smoother than a local estimator, increasing the risk of oversmoothing bias. [8]

(2) Effective sample size (ESS) decomposition

To separate information loss due to local kernel smoothing from additional weight concentration induced by GPS weighting, ESS is decomposed into kernel ESS and final weighted ESS. The kernel ESS at exposure level *a* is defined as

$${ESS}_{K}(a)=1/\sum_{i} {\tilde{K}_{i}(a)}^{2},$$

where $K_{i}(a)$ represents the kernel weight, and $\tilde{K}_{i}(a)=K_{i}(a)/\sum_{i} K_{i}(a)$. It reflects the amount of local empirical support supplied by the kernel window alone. The final weighted ESS is computed as

$${ESS}_{W}(a)=1/\sum_{i} {W_{i}(a)}^{2},$$

where $W_{i}(a)$ denotes the final normalized kernel inverse-GPS weight used in the estimator that sums to one. Following the standard design-effect interpretation, ESS captures the number of equally weighted observations that would yield the same precision as the weighted sample.[12, 13] It is suggested that extremely low ESS values (e.g., approaching single digits) may indicate severe local sample insufficiency and unreliable estimates. Furthermore, we calculate their ratio as

$$R_{ESS}\left( a \right)=\frac{ESS_{W}\left( a \right)}{ESS_{K}\left( a \right)},$$

to quantify additional information loss caused by GPS weighting beyond that induced by kernel localization. The $R_{ESS}\left( a \right)$ value closer to one suggests that GPS weighting adds little additional concentration, whereas smaller values indicate greater influence of extreme GPS-based weights. For bandwidth-level summaries, we report the minimum values of ${ESS}_{K}(a)$, $ESS_{W}\left( a \right)$ and $R_{ESS}\left( a \right)$ across the exposure range and across the four potential-outcome weighting components.

(3) Active-sample weight coefficient of variation (CV_active_)

To evaluate the dispersion and stability of the final GPS-based weights among observations that contributed to the local estimate, the active-sample CV is calculated as

$$CV_{\mathrm{act}ive}(a)=\frac{sd\{W_{i}(a):W_{i}(a)>0\}}{\text{mean}\{W_{i}(a):W_{i}(a)>0\}}.$$

This diagnostic focuses on weight variability within the active kernel window. Inspection of the distribution, variability, and extremity of inverse probability weights is commonly recommended in weighting analyses.[14, 15] A higher CV value indicates greater concentration of influence among a smaller number of observations and therefore greater potential instability. Specifically, a CV value lower than 1 corresponds to the retention of more than 50% of the effective information, suggesting a relatively uniform weight distribution, and a CV higher than 3 may warn that less than 10% of the effective information remains. For bandwidth-level summaries, we report the maximum $CV_{\mathrm{act}ive}(a)$ across the exposure evaluation range and across the four potential-outcome weighting components.

(4) Standardized mean difference (SMD)

To assess covariate balance after GPS weighting, we compute SMD separately for each potential-outcome weighting component and the covariate set involved in the corresponding GPS model. For the factual components $\hat{\mu}\left( a_{1},a_{1} \right)$ and $\hat{\mu}\left( a_{0},a_{0} \right)$, the weights depend on the GPS model $f(A|X)$, so balance is evaluated for $X_{ax}$. Conversely, for the counterfactual components $\hat{\mu}\left( a_{1},a_{0} \right)$ and $\hat{\mu}\left( a_{0},a_{1} \right)$, the weights incorporate both $f(A|X)$ and $f(A|M,X)$, so balance is checked for $X_{ax}\cup X_{amx}$. For a given exposure level *a*, weighting components *s* ∈ {1,2,3,4} corresponding to the four mean potential outcomes, and covariate $X_{j} (j=1, ..., p_{s}$, where $p_{s}$ is the dimension of the relevant covariate set), the SMD is defined as:

$${SMD}_{j,s}\left( a \right)=\frac{\left| \sum_{i\mathcal{\in I}} \tilde{W}_{i,s}\left( a \right)X_{ij}-\bar{X}_{j} \right|}{S_{j}}$$

where $\tilde{W}_{i,s}\left( a \right)$ are the normalized weights under scheme *s*, $\mathcal{I}$ denotes the trimmed analytic sample, $\bar{X}_{j}=n^{-1}\sum_{i=1}^{n} X_{ij}$ is the unweighted mean of covariate $j$, and $S_{j}=\sqrt{\left( n-1 \right)^{-1}\sum_{i=1}^{n} \left( X_{ij}-\bar{X}_{j} \right)^{2}}$ denotes its pre-weighting standard deviation.[16] For each exposure level and weighting component, the maximum absolute SMD across covariates is calculated as

$$SMD_{s}^{max}\left( a \right)=\max_{1\leq j\leq p_{s}} \left| SMD_{j,s}\left( a \right) \right|.$$

The overall maximum imbalance at exposure level *a* is then defined as

$${SMD}^{max}\left( a \right)=\max_{s\in\{1,2,3,4\}} \left| SMD_{s}^{max}\left( a \right) \right|.$$

To summarize covariate balance across exposure evaluation points, we calculate an ESS-weighted average of the component-specific maximum SMD as

$$\bar{SMD}_{max}^{ESS}=\frac{\sum_{a\in\mathcal{A}} \sum_{s=1}^{4} {ESS}_{W,s}\left( a \right)\cdot SMD_{s}^{max}\left( a \right)}{\sum_{a\in\mathcal{A}} \sum_{s=1}^{4} {ESS}_{W,s}\left( a \right)}.$$

SMD values below 0.1 are generally interpreted as indicating negligible imbalance, whereas values below approximately 0.25 have been used as a broader practical bound in propensity score diagnostics.[16-18]

**C.3 Bandwidth candidate sets and interpretation**

For the simulated scenarios, we primarily examine the diagnostic metrics for the three bandwidths: *h_wp_*, *h_wp us_*, and the rule-of-thumb bandwidth (*h_ROT_ =C*·sd(*A*)·*n*^-1/5^). This set of candidate bandwidths is determined by referencing the original literature for this estimator (Huber et al., 2020)[3] and fundamental kernel estimation theory[6, 7]. For each bandwidth, we assess all exposure level points $\{-1.0, -0.9, \ldots, -0.1, 0.1, \ldots, 0.9, 1.0\}$ and yield a comprehensive evaluation.

For the empirical application, the candidate set is considered as:

$$\{h_{wp}, h_{wp us}, 0.75h_{ROT}, h_{ROT}, 1.25h_{ROT},1.5h_{ROT}, 2h_{ROT}\}.$$

This range incorporate the simulation bandwidths and a set of ROT-based candidates spanning the conventional sensitivity range around a reference bandwidth $\left[ 0.5h_{ref}, 2h_{ref} \right]$.[19, 20] For each candidate bandwidth, we examine the range of exposure values from 3 to 3, with an increment of 1, to determine the final metrics.

## Text D. Simulation under Normally Distributed Errors.

To provide a reference scenario consistent with the Gaussian quasi-likelihood specification adopted in the proposed algorithm, we introduce additional simulation scenarios under normally distributed error terms. We generate the pre-treatment covariates ***X*** are from a multivariate normal distribution (0, $\sum$), where the covariance matrix $\sum$ is set to $\sum_{ij}=\rho(i\neq j)$ or $\sum_{ij}=1 (i=j)$. Consistent with the definition of the main simulation analysis, treatment *A* is defined as a linear function of the observed variables *X* and an unobserved *W*, mediator *M* is a linear function of the *A*, *X* and an unobserved *V*, and outcome *Y* is a linear function of *A*, *M*, *X*, and an unobserved *U*. The unobserved variables *U*, *W*, *V* are independent of each other and follow standard normal distributions with a mean of zero and variances of 1. Specifically, the data for treatment, outcome, and mediators are regenerated as follows:

$$A\boldsymbol{=}\sum_{j=1}^{p} \gamma_{j}X_{j}+W, W \sim N(0, 1)$$

$M=0.3A+0.3(X_{1}+X_{6})+V, V \sim N(0, 1)$

$Y=A+0.3M+\sum_{j=1}^{p} \beta_{j}X_{j}+U, U \sim N(0, 1)$.

Except for the distribution of error terms, other settings and calculations remain unchanged.

## Text E. Robustness Check of Interaction and Nonlinear Settings.

To examine the robustness of the proposed GOAL-based method under moderate violations of no- interaction and linearity assumptions, we conduct a sensitivity analysis using interactive and nonlinear outcome models to evaluate the estimation performance.

Firstly, we incorporate a treatment–mediator interaction term into the outcome generation model as follows:

$Y=A+0.3M+\sum_{j=1}^{p} \beta_{j}X_{j}+0.4AM+U, U\sim uniform\left( -2,2 \right);$

In this case, the direct effects are defined as $\theta_{a, a^{'}}\left( a \right)=(a-a^{'})+0.12{(a-a^{'})}^{2}$ and $\theta_{a, a^{'}}\left( a^{'} \right)=a-a^{'}$, and the indirect effects are defined as $\delta_{a, a^{'}}\left( a \right)=0.09(a-a^{'})+0.12{(a-a^{'})}^{2}$ and $\delta_{a, a^{'}}\left( a^{'} \right)=0.09(a-a^{'})$.

Secondly, we fit a nonlinear outcome model with the treatment-mediator interaction term and the quadratic terms in both the treatment and the mediator as follows:

$$Y=A+0.3M+\sum_{j=1}^{p} \beta_{j}X_{j}+0.3AM+0.2A^{2}+0.2M^{2}+U, U \sim uniform (-2, 2);$$

in this case, the direct effects are defined as $\theta_{a, a^{'}}\left( a \right)=(a-a^{'})+0.45{(a-a^{'})}^{2}$ and $\theta_{a, a^{'}}\left( a^{'} \right)=(a-a^{'})+0.3{(a-a^{'})}^{2}$, and the indirect effects are defined as $\delta_{a, a^{'}}\left( a \right)=0.09(a-a^{'})+0.177{(a-a^{'})}^{2}$ and $\delta_{a, a^{'}}\left( a^{'} \right)=0.09(a-a^{'})+0.027{(a-a^{'})}^{2}$. Except for the addition of interaction and non-linearity to the outcome model, the other steps remain unchanged. The sensitivity analysis is conducted across Scenarios 1-3 based on sample size and covariate dimension (*n*, *p*) of (2000, 1000) and covariate correlation *ρ*=0, with kernel bandwidth *h* set to *C*·*n*^-0.25^, where *C*=2.34.

## Text F. Sparsity Tests for Different Numbers of True Active Signals.

To evaluate the robustness of our proposed GOAL-based method under different sparsity structures, we incrementally increase the number of true active signals in the data generation process.

Specifically, we add two scenarios with the proportion of true signals (i.e., confounders and prognostic variables) separately increased to 10% and 20%: 1) define $X_{j} (j=1, ..., 5)$ as true confounders ($X_{C}$), $X_{j} (j=6, ..., 10)$ as prognostic variables ($X_{P}$), and $X_{11}$ and $X_{15}$ as instrumental variables ($X_{I}$); 2) define $X_{j} (j=1, ..., 10)$ as true confounders ($X_{C}$), $X_{j} (j=11, ..., 20)$ as prognostic variables ($X_{P}$), and $X_{21}$ and $X_{25}$ as instrumental variables ($X_{I}$). All other variables not mentioned are deemed as spurious covariates ($X_{S}$). The analysis is based on the sample of (*n*, *p*) = (2000, 1000) and covariate correlation *ρ*=0, with undersmoothing kernel bandwidth under Scenario 1.

## Text G. Sensitivity Analysis under Varying Covariate-to-Sample Size Ratios.

To investigate the performance of our proposed method under increasing covariate numbers, we conduct an exploratory analysis by fixing the sample size *n* at 2000, and incrementally increasing the number of covariates to target covariate-to-sample size (*p*/*n*) ratios. Due to the incompatibility of the GOAL variable selection method for settings where the number of covariates exceeds the sample size (*p* > *n*), and the substantial computational cost of our estimation procedure, we restrict the analysis to a sequence of *p*/*n* ratios where *p*<*n*: {0.1, 0.2, 0.3, 0.4, 0.5, 0.6, 0.7, 0.8, and 0.9}. The analysis is performed using kernel bandwidth *h* set to (*C*·*n*^-0.25^)/2, where *C*=2.34, under Scenarios 1 and covariate correlation *ρ*=0.

## Text H. LASSO and Adaptive LASSO Estimates.

**H.1 LASSO estimates.**

Suppose the GPS models for *i*th observation are given by $f_{A_{i}}\left( X_{i}, \gamma_{x} \right)=f\left( A_{i}=a | X_{i},\gamma_{x} \right)=\sum_{j=1}^{p} {\gamma_{x}}_{j}X_{ij}$ and $f_{A_{i}}\left( X_{i}, \gamma_{mx} \right)=f\left( A_{i}=a | {M_{i}, X}_{i},\gamma_{mx} \right)=\delta M_{i}+\sum_{j=1}^{p} {\gamma_{mx}}_{j}X_{ij}$. The LASSO estimates $\hat{\gamma}_{x}$ and $\hat{\gamma}_{mx}$ are defined as

$\hat{\gamma}_{x}(LASSO)=arg\min_{\gamma_{x}} \left. ||A-\sum_{j=1}^{p} X_{j} \right.{{\gamma_{x}}_{j}||}^{2}+\lambda_{x}\sum_{j=1}^{p} \left| {\gamma_{x}}_{j} \right|$ (A1)

and

$\hat{\gamma}_{mx}(LASSO)=arg\min_{\gamma_{mx}} \left. ||A-\sum_{j=1}^{p} X_{j}{\gamma_{mx}}_{j}-\zeta M \right.{||}^{2}+\lambda_{mx}\sum_{j=1}^{p} \left| {\gamma_{mx}}_{j} \right|$ (A2)

where $\lambda_{x}$ and $\lambda_{mx}$ are nonnegative regularization parameters respectively for two GPS models.

**H.2 Adaptive LASSO estimates.**

Adaptive LASSO (AdaLASSO) is one variant of the conventional LASSO and the estimates $\hat{\gamma}_{x}$ and $\hat{\gamma}_{mx}$ for the aforementioned GPS models are defined as

$\hat{\gamma}_{x}(AdaLASSO)=arg\min_{\gamma_{x}} \left. ||A-\sum_{j=1}^{p} X_{j} \right.{{\gamma_{x}}_{j}||}^{2}+\lambda_{x}\sum_{j=1}^{p} \hat{\omega}_{j}\left| {\gamma_{x}}_{j} \right|$ (A3)

and

$\hat{\gamma}_{mx}(AdaLASSO)=arg\min_{\gamma_{mx}} \left. ||A-\sum_{j=1}^{p} X_{j}{\gamma_{mx}}_{j}-\zeta M \right.{||}^{2}+\lambda_{mx}\sum_{j=1}^{p} \hat{\omega}_{j}\left| {\gamma_{mx}}_{j} \right|$ (A4)

where $\lambda_{x}$ and $\lambda_{mx}$ are nonnegative regularization parameters respectively for two GPS models, $\hat{\omega}_{j}=\left| \hat{\beta}_{j} \right|^{-\tau}$ (τ > 1), where $\hat{\beta}_{j}$ is the unpenalized maximum likelihood estimate of the *j*th covariate and can be obtained by fitting the linear regression of *A* on *X* for $\hat{\gamma}_{x}(AdaLASSO)$ and by fitting the linear regression of *A* on *X* and *M* for $\hat{\gamma}_{mx}(AdaLASSO)$, respectively.

## Text I. Assessment of Key Assumptions in the Real Data Analysis.

Based on the covariates selected in the real-data application, the sequential conditional independence of the treatment and the mediator (corresponding to Assumption 3) is invoked to identify mediation effects. Given that the regularization methods embedded in the GPS models in the semi-parametric estimation are designed to identify relevant covariates associated with the outcome, mediator, and treatment, they may help capture important confounding structures within the high-dimensional covariate space. Consequently, this strategy can help reduce potential confounding bias and improve the plausibility of the conditional independence assumption in practice.

To inspect whether the use of GPS $f_{A}\left( a | X \right)$ and $f_{A}\left( a | M,X \right)$ achieves the balance between the distributions of covariates and mediator across the common support of treatment, we conduct a linear regression of the selected set of covariates by different regularization methods as well as the mediator *M* on the treatment *A*, $f_{A}\left( a | M,X \right)$ or $f_{A}\left( a | X \right)$ estimated at sample values of *A* and its square, following Smith and Todd.[21] Non-significant association between (*X*, *M*) and *A* given GPS suggests an achievement of the balancing property. The examination results show that the coefficients of all the 45 variables selected by the GOAL method and *M* on the treatment conditional on GPSs are statistically insignificant, indicating no signs of violating the balancing property. A similar result is also observed for AdaLASSO or LASSO-based GPS models, and the full GPS model that includes all the covariates.

We also examine the common support across the GPSs at different treatment values to verify whether excessively large weights are obtained by calculations based on the GPSs close to zero (Assumption 2). Observations that receive extremely large weights can entail larger impacts on the estimation of the mean potential outcome, introducing large variance and thus reducing estimation efficiency. For instance, the relative weight for each observation for $\hat{\mu}\left( a,a \right)$ is $\frac{K_{h}(A_{i}-a)}{\hat{f_{A}}(a|X_{i}; \hat{\gamma}_{x})}/\sum_{i=1}^{n} \frac{K_{h}(A_{i}-a)}{\hat{f_{A}}(a|X_{i}; \hat{\gamma}_{x})}$ (Equation 7). For the four mean potential outcomes ($\hat{\theta}_{a, a^{'}}\left( a \right)$, $\hat{\theta}_{a, a^{'}}\left( a^{'} \right)$, $\hat{\delta}_{a, a^{'}}\left( a \right)$ and $\hat{\delta}_{a, a^{'}}\left( a^{'} \right)$) across the treatment range of {3, 4, ..., 13}, the relative weight of each observation obtained by the GPSs (based on all the regularization methods and the full covariate set) is below 1%. These examination results show evidence of the existence of common support.

**Reference:**

1. Imai K, Keele LJ, Yamamoto T. Identification, Inference and Sensitivity Analysis for Causal Mediation Effects. Statistical Science. 2010;25:51-71.

2. Tchetgen EJ, Shpitser I. Semiparametric Theory for Causal Mediation Analysis: efficiency bounds, multiple robustness, and sensitivity analysis. Ann Stat. 2012;40(3):1816-45.

3. Huber M, Hsu YC, Lee YY, Lettry L. Direct and indirect effects of continuous treatments based on generalized propensity score weighting. Journal of Applied Econometrics. 2020;35(7):814-40.

4. Cattaneo MD, Jansson M. Kernel‐based semiparametric estimators: Small bandwidth asymptotics and bootstrap consistency. Econometrica. 2018;86(3):955-95.

5. Armstrong TB, Kolesár M. Simple and honest confidence intervals in nonparametric regression. Quantitative Economics. 2020;11(1):1-39.

6. Silverman BW. Density Estimation for Statistics and Data Analysis: Density Estimation For Statistics And Data Analysis; 1986.

7. Wand MP, Jones MC. Kernel smoothing: CRC press; 1994.

8. Gijbels I. Local polynomial modelling and its applications: Chapman & Hall; 1996.

9. Ullah A, Pagan A. Nonparametric econometrics: Cambridge university press Cambridge; 1999.

10. Calonico S, Cattaneo MD, Farrell MH. On the effect of bias estimation on coverage accuracy in nonparametric inference. Journal of the American Statistical Association. 2018;113(522):767-79.

11. Racine J, Li Q. Nonparametric estimation of regression functions with both categorical and continuous data. Journal of Econometrics. 2004;119(1):99-130.

12. Kish L. Survey sampling. new york: John wesley & sons. Am Polit Sci Rev. 1965;59(4):1025.

13. McCaffrey DF, Ridgeway G, Morral AR. Propensity score estimation with boosted regression for evaluating causal effects in observational studies. Psychological methods. 2004;9(4):403.

14. Cole SR, Hernán MA. Constructing inverse probability weights for marginal structural models. American journal of epidemiology. 2008;168(6):656-64.

15. Austin PC, Stuart EA. Moving towards best practice when using inverse probability of treatment weighting (IPTW) using the propensity score to estimate causal treatment effects in observational studies. Statistics in medicine. 2015;34(28):3661-79.

16. Austin PC. Balance diagnostics for comparing the distribution of baseline covariates between treatment groups in propensity‐score matched samples. Statistics in medicine. 2009;28(25):3083-107.

17. Austin PC. Assessing covariate balance when using the generalized propensity score with quantitative or continuous exposures. Statistical methods in medical research. 2019;28(5):1365-77.

18. Stuart EA. Matching methods for causal inference: A review and a look forward. Statistical science: a review journal of the Institute of Mathematical Statistics. 2010;25(1):1.

19. Imbens GW, Lemieux T. Regression discontinuity designs: A guide to practice. Journal of econometrics. 2008;142(2):615-35.

20. Bueno N, Tuñón G. Graphical presentation of regression discontinuity results. The Political Methodologist. 2015;22(2):4-8.

21. Smith J, Todd P. Rejoinder. Journal of Econometrics. 2005;125(1-2):365-75.
